# Development of a specific live-cell assay for native autophagic flux

**DOI:** 10.1016/j.jbc.2021.101003

**Published:** 2021-07-23

**Authors:** Nathaniel Safren, Elizabeth M. Tank, Ahmed M. Malik, Jason P. Chua, Nicholas Santoro, Sami J. Barmada

**Affiliations:** 1 Department of Neurology, University of Michigan, Ann Arbor Michigan, USA; 2 Center for Chemical Genomics, Life Sciences Institute, University of Michigan, Ann Arbor Michigan, USA

**Keywords:** ALS, autophagy, FTD, high-content, high-throughput, neurodegeneration, photoconvertible, repurposing, screen, stem cell, ALS, amyotrophic lateral sclerosis, AM, automated microscopy, ATG, autophagy-related gene, CNS, central nervous system, CRISPR, clustered regularly interspaced short palindromic repeats, DMEM, Dulbecco’s modified Eagle medium, DMSO, dimethyl sulfoxide, EC50, half-maximal effective concentration, FTD, frontotemporal dementia, GFP, green fluorescent protein, HDR, homology directed repair, HEK, human embryonic kidney, IC50, half-maximal inhibitory concentration, iMN, induced motor neuron, iNeuron, induced neuron, iRFP, near-infrared fluorescent protein, mTOR, mammalian target of rapamycin, OPL, optical pulse labeling, ORF, open reading frame, PI3K, phosphoinositide 3-kinase, PKC, protein kinase C, RFP, red fluorescent protein, sgRNA, single-guide ribonucleic acid, TALEN, transcription activator-like effector nuclease

## Abstract

Autophagy is an evolutionarily conserved pathway mediating the breakdown of cellular proteins and organelles. Emphasizing its pivotal nature, autophagy dysfunction contributes to many diseases; nevertheless, development of effective autophagy modulating drugs is hampered by fundamental deficiencies in available methods for measuring autophagic activity or flux. To overcome these limitations, we introduced the photoconvertible protein Dendra2 into the *MAP1LC3B* locus of human cells *via* CRISPR/Cas9 genome editing, enabling accurate and sensitive assessments of autophagy in living cells by optical pulse labeling. We used this assay to perform high-throughput drug screens of four chemical libraries comprising over 30,000 diverse compounds, identifying several clinically relevant drugs and novel autophagy modulators. A select series of candidate compounds also modulated autophagy flux in human motor neurons modified by CRISPR/Cas9 to express GFP-labeled LC3. Using automated microscopy, we tested the therapeutic potential of autophagy induction in several distinct neuronal models of amyotrophic lateral sclerosis (ALS) and frontotemporal dementia (FTD). In doing so, we found that autophagy induction exhibited discordant effects, improving survival in disease models involving the RNA binding protein TDP-43, while exacerbating toxicity in neurons expressing mutant forms of UBQLN2 and C9ORF72 associated with familial ALS/FTD. These studies confirm the utility of the Dendra2-LC3 assay, while illustrating the contradictory effects of autophagy induction in different ALS/FTD subtypes.

Macroautophagy (hereafter referred to as autophagy) is an essential pathway for protein homeostasis whereby cytoplasmic proteins and organelles are delivered to lysosomes for degradation (1). Through the coordinated action of a series of autophagy-related (ATG) proteins and cargo receptors including p62/SQSTM1, NBR1, and optineurin (2), substrates are sequestered within double-membrane vesicles called autophagosomes. Autophagosomes mature as they traffic along microtubules and eventually fuse with lysosomes to form autolysosomes, wherein hydrolases degrade autophagic cargo. The protein LC3 (ATG8) is an obligate component of autophagosome membranes and is itself degraded within autolysosomes. For these reasons, it often serves as both a marker of autophagosomes and a representative autophagy substrate (3).

Underscoring the critical requirement of autophagy in cellular homeostasis, deletion of core autophagy genes in mice results in embryonic lethality (4, 5, 6). Accordingly, dysfunctional autophagy is linked to a wide spectrum of human diseases including neurodegeneration, cancer, metabolic disorders, infectious and cardiovascular diseases (7). Often these conditions involve deficiencies in one or more steps of autophagy, resulting in impaired clearance of potentially toxic cellular components and/or a failure to replenish amino acids required for anabolic processes. In these instances, enhancing the rate of autophagic cargo clearance, commonly referred to as flux, would be beneficial. Conversely, autophagy can promote tumor progression and resistance to chemotherapy for some cancers (8, 9, 10, 11). Here, autophagy inhibition may represent a more apt therapeutic strategy (7).

Autophagy is of particular importance in the central nervous system (CNS). Deletion of essential autophagy genes within the CNS of mice leads to progressive neurodegeneration marked by accumulation of protein aggregates (12, 13, 14, 15, 16). Defective autophagy is a common feature of many neurodegenerative diseases including Alzheimer’s disease (17, 18), Parkinson’s disease (19, 20, 21, 22, 23), polyglutamine disorders (24, 25, 26), amyotrophic lateral sclerosis (ALS) (27), and frontotemporal dementia (FTD) (28, 29, 30, 31). Moreover, mutations in several autophagy-related proteins including p62/SQSTM1 (32), optineurin (33), C9ORF72 (34, 35) TBK1 (36), and UBQLN2 (37) result in familial ALS and FTD.

Due to its broad therapeutic potential, autophagy modulation has received considerable attention as a target for drug development (7). Nevertheless, these efforts have thus far failed to translate into effective therapies for patients. This is in part due to the intrinsic difficulties in measuring autophagic flux and consequent inability of many conventional and widely used autophagy assays to accurately estimate flux (3). One prominent limitation of these assays is an implicit reliance on the steady-state abundance of pathway intermediates such as LC3-II, the lipidated isoform of LC3. Due to the dynamic nature of autophagy, changes in such intermediates may equally reflect increased autophagy induction or late-stage inhibition of autophagsome clearance; although discriminating among these mechanisms is crucial for drug development, many assays are effectively unable to do so. While lysosomal inhibitors such as bafilomycin-A1 can be used to isolate autophagy induction from inhibition, this approach obscures estimates of substrate clearance, perhaps the most relevant measure of autophagic flux. Bafilomycin-A1 and related compounds are also inherently toxic, further confounding flux measurements (38, 39). Yet another common shortcoming is an inherent reliance on static “snapshots” of separate cellular populations that cannot be followed prospectively or longitudinally due to the need for cell lysis and measurement of pathway intermediates.

We previously developed a technique called optical pulse labeling (OPL), enabling noninvasive measurements of autophagic flux in living cells without the need for lysosomal inhibition (40). In this technique, LC3 is labeled with the photoconvertible protein Dendra2 (41). Upon exposure to blue (405 nm) light, Dendra2 fluorescence irreversibly shifts from green to red. Since the generation of red-fluorescent Dendra2-LC3 is limited by blue light, LC3 turnover can be determined independent of new protein synthesis by tracking the time-dependent reduction in red fluorescence following a brief pulse of blue light (Fig. 1*A*). LC3 is an autophagy substrate, and therefore its degradation kinetics serves as a faithful proxy for estimates of autophagic flux. While OPL offered several advantages over conventional assays, it was nonetheless limited by its reliance on protein overexpression; in effect, Dendra2-LC3 overexpression floods the pathway under investigation with an obligate substrate. Burdening the cell with nonphysiological concentrations of substrate might artificially prolong flux estimates or conversely enhance flux *via* regulatory feedback mechanisms. Moreover, because autophagy regulation is intricately tied to amino acid availability (42, 43) and the ubiquitin proteasome system (44), any perturbations to these pathways brought on by protein overexpression may further confound measurements of flux.

**Figure 1:**
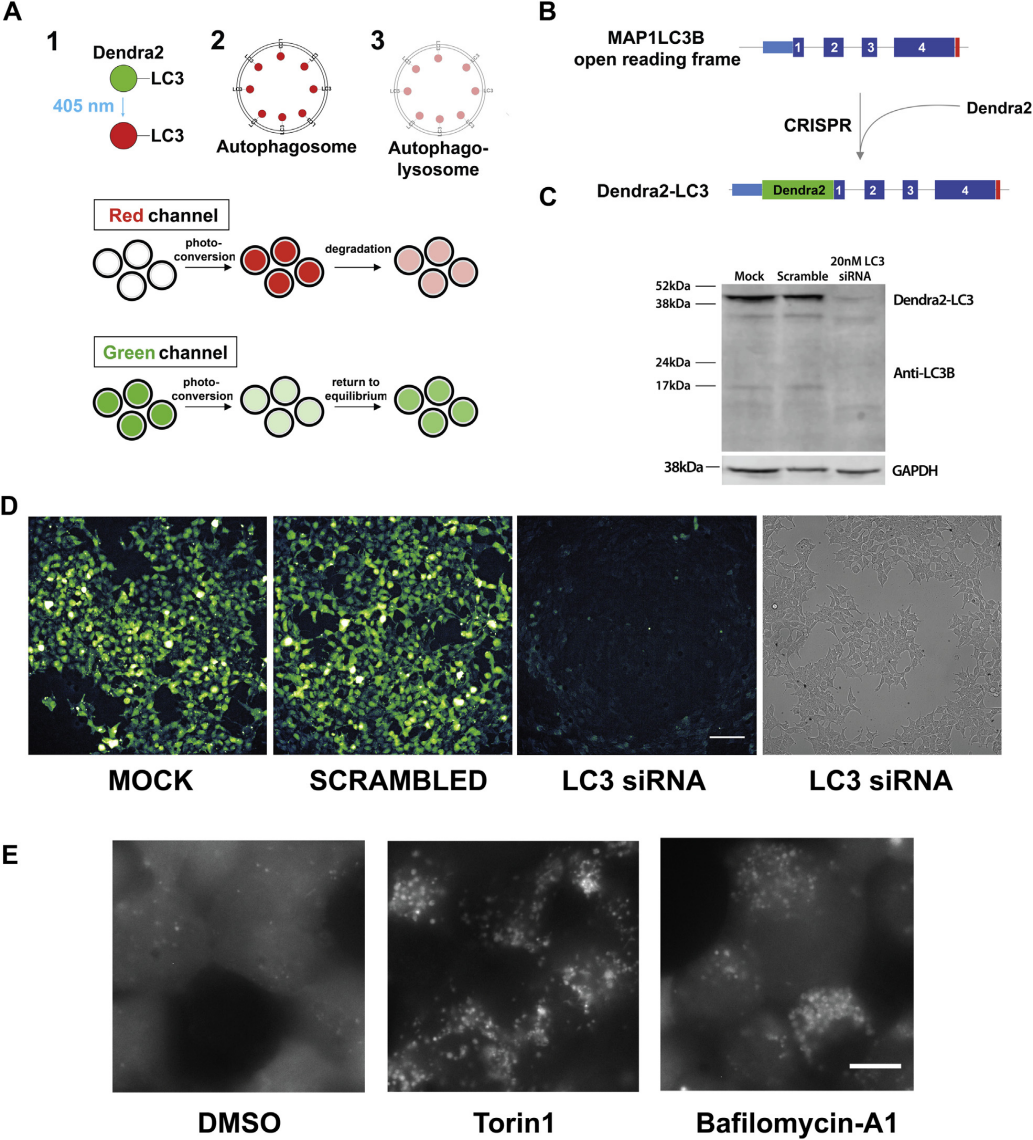
**Creation of a stable cell line serving as a reporter for autophagic flux.***A*, illustration depicting the use of optical pulse labeling (OPL) to measure autophagic flux. 1, Dendra2-LC3 is a photoconvertible fusion protein that irreversibly shifts its fluorescence from green to red upon exposure to 405 nm light. 2, Dendra2-LC3 is an autophagy substrate that is incorporated into autophagosomes. Prior to degradation, red fluorescence is high. 3, autophagosomes mature into autophagolysosomes, where photoconverted Dendra2-LC3 is degraded over time, resulting in a drop in red fluorescence intensity. The time-dependent decay of red signal serves as an estimate of autophagic flux, independent of new (*green*) LC3-Dendra2 synthesis. *B*, schematic for tagging native LC3 using CRISPR/Cas9 genome editing. In HEK293T cells, the Dendra2 ORF was introduced into the *MAP1LC3B* locus upstream of exon 1 creating an N-terminal fusion protein upon translation. *C*, western blot confirming the successful labeling of LC3 with Dendra2. Dendra2-LC3 HEK293T cells were treated with 20 nM siRNA targeting LC3 or scrambled siRNA. Lysates were collected after 48h and immunoblotted with an LC3 antibody, demonstrating the Dendra2-LC3 fusion protein running at the expected MW of 43 kDa that disappears upon siRNA-mediated knockdown of LC3. GAPDH serves as a loading control. *D*, Dendra2-LC3 reporter line imaged in the GFP and bright-field channels 48h after application of siRNA. Scale bar = 100 μm. *E*, Dendra2-LC3 cells imaged 6h after treatment with vehicle, 1 μM Torin1, and 20 nM Bafilomycin-A1. Scale bar = 10 μm.

To overcome these drawbacks, we labeled endogenous LC3 with Dendra2 using CRISPR/Cas9 editing, producing a novel cell line capable of assaying autophagic flux in living cells without the need for drug treatment, protein overexpression, or measurements of pathway intermediates, thus establishing a faithful reporter of native autophagy activity unadulterated by exogenous manipulations. Leveraging this cell line for its unique perspective on autophagy and the opportunities it presents, we adapted Dendra2-LC3 cells for conducting high-content screens and identified several new and active autophagy modulators with promising therapeutic properties. Many of these compounds also modulate autophagy in human motor neurons, an ALS/FTD relevant cell-type, and impact their survival in disease models. Surprisingly, however, we found that autophagy induction failed to consistently improve neuronal survival, instead displaying distinct effects that varied depending on the underlying pathogenic mutation.

## Results

### Creation of a novel reporter of autophagic flux

We employed CRISPR/Cas9 genome editing to label native LC3 by introducing Dendra2 into the *MAP1LC3B* locus (encoding LC3) of human embryonic kidney (HEK) cells (45) (Fig. 1*B*). To minimize the risk of undesired insertions/deletions *via* nonhomologous end joining, we used a dual-nickase strategy (46), in which Cas9(D10A) was expressed along with two single-guide RNAs (sgRNAs) targeting sequences immediately upstream and downstream of the *MAP1LC3B* start codon. Unlike wild-type Cas9, Cas9(D10A) induces single-stranded nicks rather than double-stranded breaks in the DNA, limiting recombination to the region marked by the sgRNAs. In addition, a vector containing the Dendra2 open reading frame (ORF) flanked by 400 bp of homologous sequence 5′ and 3′ to the *MAP1LC3B* start codon was introduced to facilitate homology directed repair (HDR), thereby creating a sequence encoding Dendra2 fused to the N-terminus of LC3. Positive cells were selected based on Dendra2 fluorescence and enriched by sequential passaging until a homogeneous population was achieved. Western blotting (Fig. 1*C*) and Sanger sequence (Fig. S1*A*) confirmed the successful insertion of the Dendra2 ORF into the desired locus. Transfection with siRNA targeting LC3 substantially reduced both Dendra-LC3 protein levels and native GFP fluorescence, providing further validation of successful on-target CRISPR editing (Fig. 1, *C* and *D*).

In untreated cells, Dendra2-LC3 fluorescence was diffusely distributed, matching the predicted localization of the nonlipidated, cytosolic LC3-I isoform (Fig. 1*E*, Supplemental Movie 1). Treatment with the potent autophagy inducer Torin1 (47) elicited the *de novo* formation of visible fluorescent puncta and reduced the intensity of diffuse Dendra2-LC3 (Fig. 1*E*, Supplemental Movie 2), reflecting the incorporation of Dendra2-LC3 into autophagosome membranes. In agreement with previous studies of autophagosome dynamics (26, 48), live cell imaging revealed that a subset of Dendra2-LC3 puncta were highly mobile (Supplemental Movie 2). As expected, inhibiting the clearance of autophagosomes *via* treatment with the lysosomal V-ATPase inhibitor bafilomycin-A1 leads to the accumulation of large bright puncta without an accompanying decrease in diffuse Dendra2-LC3 fluorescence (Supplemental Movie 3). Together these data confirm that Dendra2-tagged version of LC3 behaves as expected in modified HEK293T cells (40) and that these cells can be used to visualize autophagy modulation by a variety of stimuli.

### Development and validation of an autophagic flux assay

**Figure 2:**
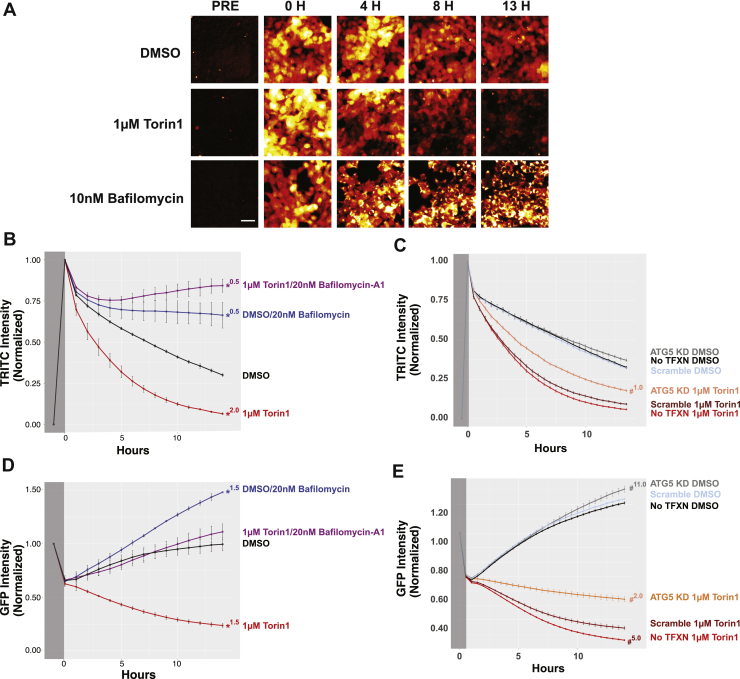
**Time-dependent decay of Dendra2-LC3 serves as an accurate measure of autophagic flux.***A*, Dendra2-LC3 HEK293T cells were imaged prior to photoconversion to measure background RFP intensity. Immediately following photoconversion, cells were treated with DMSO, 1 μM Torin1, or 10 nM Bafilomycin-A1 and imaged at the indicated times. Images are pseudocolored to better highlight intensity differences. Scale bar = 50 μm. *B*–*E*, time-dependent changes in photoconverted Dendra2-LC3 fluorescence in the RFP (*B* and *C*) and GFP (*D* and *E*) channels. Intensity measurements were obtained prior to (*dark gray*) and following photoconversion (*light gray*) and normalized. For RFP measurements, the background intensity prior to conversion was set to 0 and the postconversion value to 1. GFP values are scaled to the preconversion intensity. Error bars represent SEM from three replicate experiments. *B*, treatment with 1 μM Torin1 accelerates Dendra2-LC3 decay, reflecting enhanced autophagic degradation of the reporter, while treatment with bafilomycin-A1 stabilizes reporter half-life. *D*, photoconversion results in a 40% drop in GFP intensity. As new Dendra2-LC3 is synthesized, GFP levels return to prephotoconversion levels over 13.5 h. Torin1 blocks the observed return in GFP fluorescence by accelerating flux. Genetic inhibition of autophagy *via* siRNA-mediated knockdown of ATG5 2 days prior attenuates Torin1’s effects in both the RFP (*C*) and GFP (*E*) channels. For (*B*–*D*), Two-Way ANOVA was performed, indicating significant effects of both time and treatment, as well as a significant interaction between time and treatment. These values are summarized in Table S1. ∗ denotes *p* < 0.05 using DMSO as reference group with Tukey’s multiple comparisons test; # indicates *p* < 0.05 with the scramble control for each drug treatment as the reference group (*i.e.*, scramble siRNA 1 μM Torin1 *versus* ATG5 siRNA 1 μM Torin1). *Superscript number* indicates the first time point when significance was achieved.

In these cells, endogenous Dendra2-LC3 could be efficiently photoconverted with minimal toxicity using 4s pulses of 405 nm light, producing a strong red signal (Fig. 2*A*) concurrent with a substantial reduction in green fluorescence. Using time-lapsed microscopy, we measured fluorescence intensity in both the red (TRITC) and green (GFP) channels at regular intervals over the span of 13.5 h. In vehicle-treated cells red fluorescence decayed with a half-life of approximately 7.5 h. Treatment with Torin1 significantly accelerated this decay, reducing Dendra2-LC3 half-life ∼3-fold to 2.5 h. In contrast, bafilomycin-A1 completely stabilized Dendra2-LC3 and blocked the Torin1-induced reduction in Dendra2-LC3 half-life (Fig. 2, *A* and *B*). Thus, endogenous Dendra2-LC3 flux measured by OPL responds appropriately to bidirectional modulation of autophagy.

To confirm autophagy-dependent degradation of Dendra2-LC3 in modified HEK293T cells, we asked whether genetic inhibition of autophagy extended Dendra-LC3 half-life. HEK293T cells were transfected with siRNA targeting the autophagy gene ATG5 (49), achieving a marked but incomplete reduction in ATG5 levels (Fig. S1*B*). Partial ATG5 knockdown attenuated Torin1’s effects on Dendra2-LC3 half-life but had no discernible impact on Dendra2-LC3 turnover in vehicle-treated cells (Fig. 2*C*). These data show that Dendra2-LC3 clearance in response to Torin1 is mediated by autophagy and also suggest that a low level of ATG5 is sufficient for basal autophagy and only becomes rate-limiting upon autophagy induction.

Consistent with effective photoconversion of Dendra2-LC3, GFP intensity dropped by approximately 40% in pulsed cells, returning to preconversion levels within 13h (Fig. 2*D*). This return to steady-state GFP intensity likely reflects an equilibrium point at which new Dendra-LC3 production is balanced with its turnover. Treatment with Torin1 shifted this balance, not only preventing the return in GFP signal, but also further reducing GFP intensity over time. Application of bafilomycin-A1 (Fig. 2*D*) or ATG5 knockdown (Fig. 2*E*) both prevented Torin1-induced reductions in Dendra2-LC3 GFP intensity. Conversely, bafilmoycin-A1 treatment led to a supraphysiological increase in GFP signal (Fig. 2*D*). Thus, while the decay of photoconverted (red) Dendra2-LC3 can be used to accurately measure autophagic flux because it decouples protein turnover and synthesis, time-dependent changes in native (green) Dendra2-LC3 fluorescence mirror those observed in the red channel and provide complementary estimates of flux.

**Figure 3:**
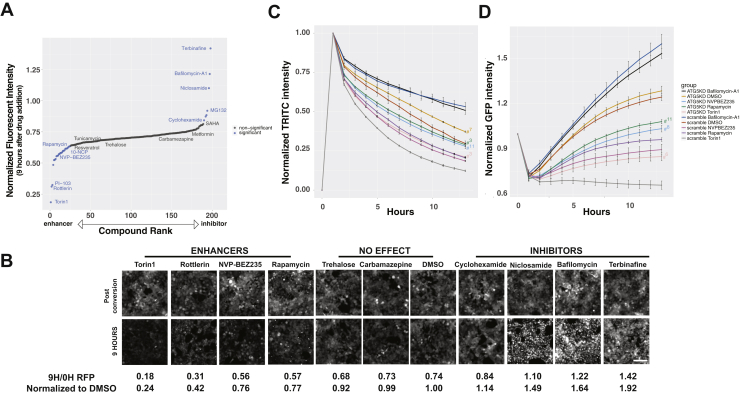
**Drug library screens in Dendra2-LC3 HEK293T cells confirm assay validity and identify new autophagy modulators.***A*, an unbiased screen of the Enzo autophagy compound library identified several known autophagy-modulating compounds, including enhancers (rapamycin, NVP-BEZ235, AKT inhibitor X) and inhibitors (bafilomycin-A1). All drugs were added at a final concentration of 10 μM *via* liquid handler, and autophagic flux estimated by their effects upon clearance of photoconverted (*red*) Dendra2-LC3 9 h after drug addition. *B*, representative images of red Dendra2-LC3 immediately postconversion and 9 h after drug addition, demonstrating relatively rapid clearance with application of enhancers and marked accumulation of Dendra2-LC3 with inhibitors. Both the 9 h/0 h RFP ratio and this value normalized to DMSO 9 h/0 h RFP intensity are listed below each set of images. Torin1 equivalents listed in Fig. 3-source data = [1 − (9 h/0 h RFP ratio)_drug_]/[1 − (9 h/0 h RFP ratio)_Torin1_]. Scale bar = 100 μm. *C*, the effect of autophagy enhancers on Dendra2-LC3 half-life is attenuated by siRNA-mediated knockdown of ATG5 2 days prior to drug application. Two-Way ANOVA indicated a significant interaction between time and treatment. F(126,300) = 15.22, *p* < 0.0001 and significant effects for treatment F(9,300) = 3427, *p* < 0.0001 and time F(14,300) = 957.3, *p* < 0.0001. *D*, similar effects are observed in the green channel. Two-Way ANOVA found a significant interaction between time and treatment F(126,300) = 20.71, *p* < 0.0001 as well as significant main effects for treatment F(9,300) = 721, *p* < 0.0001 and time F(14,300) = 320.8, *p* < 0.0001. In (*C* and *D*), error bars represent SEM from three biological replicates, # indicates *p* < 0.05 using Tukey’s multiple comparisons test with the scramble control for each drug treatment as the reference group (*i.e.*, Scramble siRNA Torin1 *versus* ATG5 siRNA Torin1). *Superscript number* indicates the first time point when significance was achieved.

To confirm that the metabolism of endogenous Dendra2-LC3 reflects autophagic flux, while simultaneously validating the use Dendra2-LC3 cells for identifying new autophagy-modulating strategies, we used the cells to screen an Enzo tool compound library that includes several drugs with purported effects on autophagy (Fig. 3*A*, Fig. 3-source data). These experiments helped gauge the generalizability of the assay beyond the effects of strong autophagy modulators such as Torin1 and bafilomycin-A1 and also helped determine its ability to identify drugs that impact autophagy through a variety of mechanisms.

Many canonical autophagy-modulating drugs demonstrated clear effects on autophagic flux (Fig. 3, *A* and *B*), establishing construct validity for the assay. Among compounds that significantly enhanced autophagic flux were the allosteric mammalian target of rapamycin (mTOR) inhibitor rapamycin and the dual phosphoinositide 3-kinase (PI3K)/mTOR inhibitors NVP-BEZ235 and PI-103. Torin1 and PI-103 exerted particularly strong effects in line with their action as ATP competitive antagonists (47, 50). 10-NCP, an AKT inhibitor that we previously identified as a neuronal autophagy-inducing compound (40, 51), also increased autophagic flux in Dendra2-LC3 HEK293T cells. Rottlerin, a compound that demonstrated autophagy-enhancing effects *via* inhibition of mTOR as well as protein kinase C (PKC) delta, likewise accelerated Dendra2-LC3 clearance (52, 53).

Bafilomycin-A1, also present in the compound library, registered as a strong inhibitor of Dendra2-LC3 flux (Fig. 3, *A* and *B*), as expected based on our initial investigations. Rather than decrease over time, the intensity of photoconverted (red) Dendra2-LC3 in cells treated with bafilomycin-A1 progressively increased, eventually exceeding levels immediately following photoconversion (Fig. 3*B*). This phenomenon reflects a peculiar imaging property unique to Dendra2-linked proteins that accumulate within large puncta, in the process sequestering diffuse, low-intensity signal within relatively small regions (Fig. S2). Thus, the time-dependent degradation of Dendra2-LC3 is inhibited by bafilomycin-A1, and the observed increase in red fluorescence intensity is due to the accumulation of Dendra2-LC3 within large clusters of perinuclear autophagosomes.

The protein translation inhibitor cyclohexamide stabilized Dendra2-LC3 turnover (Fig. 3, *A* and *B*), in keeping with autophagy inhibition downstream of amino acid accumulation and mTORC1 activation (54). This is in contrast to what is observed in the green channel, where inhibiting the synthesis of Dendra2-LC3 results in a decrease in GFP fluorescence as expected (Fig. 5-source data). These results highlight the pivotal ability of the assay to decouple autophagy inhibition from new protein synthesis.

Dendra2-LC3 RFP fluorescence was also stabilized by the proteasome inhibitors MG132, Bortezomib, and ALLN (Fig. 3*A*; Fig. S3*A*, Fig. 3-source data), albeit to a far lesser degree than with bafilomycin-A1 or other strong inhibitors. Conversely, the calpain inhibitor MDL-28170 had no discernible effect on Dendra2-LC3 degradation (Fig. S3*A*). Together these data suggest that while Dendra2-LC3 serves as valid reporter of autophagic flux, it is not degraded exclusively *via* autophagy. Therefore, to confirm that hits arising from this assay were indeed capable of affecting Dendra2-LC3 turnover *via* their actions on autophagy, we tested their effects in ATG5-deficient cells. As seen with Torin1 (Fig. 2), ATG5 knockdown attenuated the autophagy-inducing effects of NVP-BEZ235 and rapamycin (Fig. 3, *C* and *D*), verifying that these drugs stimulate Dendra2-LC3 clearance by enhancing autophagic flux.

Because of the nature of the screen, compounds exhibiting intrinsic fluorescence could result in an artificially high RFP signal, leading to their subsequent misclassification as autophagy inhibitors. To address this possibility, we rescreened all hits in unmodified HEK293T cells that do not express Dendra2. We found that four out of the 35 tested drugs, including three out of the ten drugs that were identified as inhibitors, exhibited intrinsic fluorescence in the RFP channel (Fig. 3-source data, Fig. S3*B*). For instance, curcumin, a purported autophagy modulator (55) with known autofluorescent properties, produced a substantial increase in background RFP signal that precluded any estimations of its effects on autophagy in this assay. In contrast, the PKC inhibitor bim-1 had little effect on background fluorescence, but instead accumulated within perinuclear autofluorescent puncta that resembled those observed in cells treated with bafilomycin-A1. These results underscore the importance of counterscreening to exclude intrinsically fluorescent drug properties that can confound or obscure results in this and other fluorescence-based assays.

Using Torin1 and bafilomycin-A1, we next evaluated the sensitivity of Dendra2-LC3 cells for detecting small changes in autophagic flux in a dose-dependent manner. We tested the effects of ten serial dilutions of each drug in both the GFP and RFP channels. We observed a tunable and proportional response to increasing drug concentrations for both Torin1 and bafilomycin-A1 (Fig. S4*A*). This was perhaps most evident for bafilomycin-A1, where the assay had sufficient resolution to discriminate 2 nM changes in concentration (Fig. S4, *C* and *E*). Notably, the GFP channel was nearly as sensitive as the RFP channel for detecting differences in autophagic flux (Fig. S4, *D* and *E*). While the GFP fluorescence gradually returned to equilibrium in vehicle-treated cells over a 12h span, it continued to drop with Torin1 treatment in a dose-dependent manner (Fig. S4*D*). Conversely, the GFP intensity quickly surpassed prephotoconversion levels in cells treated with bafilomycin-A1, and the rate of increase was proportional to the drug dose (Fig. S4*E*). For Torin1, bafilomycin-A1, rapamycin, and NVP-BEZ235, the dose–response relationships for each drug were strikingly similar between channels, producing nearly identical half maximal effective concentration (EC50) and half maximal inhibitory concentration (IC50) values for each compound (Fig. S4*F*).

### Establishing a high-content screening platform for autophagy modulators

**Figure 4:**
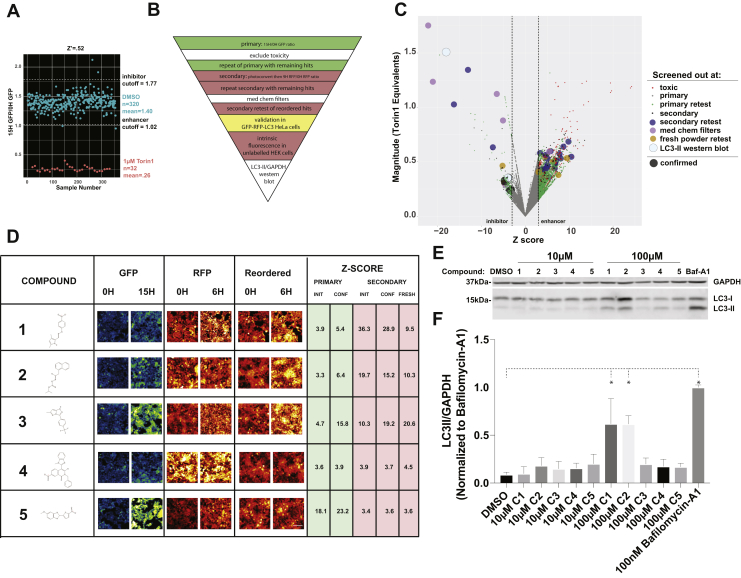
**High-throughput screening identifies novel autophagy inhibitors.***A*, “90/10” experiment validating Dendra-LC3 intensity in the GFP channel as an indicator of autophagy flux. 320 wells of a 384w plate were treated with DMSO and 32 were treated with 1 μM Torin1. Plates were imaged immediately before, and 15 h after drug treatment. Z’=0.52 ± 0.04 in three replicates. *B*, schematic depicting the screening hierarchy used. *C*, the primary screen utilized the Maybridge 24K library, consisting of 24,000 chemically diverse compounds. For enhancers, changes in Dendra2-LC3 GFP intensity were normalized to Torin1’s effects using the formula [1 − (9 h/0 h RFP ratio)_drug_]/[1 − (9 h/0 h RFP ratio)_Torin1_]. Nontoxic compounds that passed the primary screen were filtered by retesting in the GFP channel, then evaluated in a secondary screen involving calculation of Dendra2-LC3 half-life in the RFP channel. Hits were retested in the secondary screen, followed by repeat evaluation in the RFP channel using fresh compound from a different distributor. The color and size of each *dot* denote the stage at which individual compounds were eliminated, in accordance with the key. Candidates that passed all filters are shown in *black*. *D*, representative images of the primary screen, secondary screen, and repeat secondary screen with fresh drug for five putative autophagy inhibitors, ranked based on the magnitude of inhibition measured in the secondary screen. Z-scores are reported for each screening phase. Scale bar = 100 μm. *E*, unmodified HEK293T cells were treated with vehicle or each compound at either 10 μM or 100 μM. Lysates were collected 9h after treatment and immunoblotted with an LC3 antibody. *F*, quantification of three biological replicates demonstrating significant inhibition autophagic flux by compounds 1 and 2 at 100 μM. For each group LC3-II was normalized to the loading control (GAPDH) and scaled to 100 nM bafilomycin-A1. Error bars represent standard error of the mean. One-way ANOVA showed significant differences between groups (F = 24.28, *p* < 0.0001), ∗*p* < 0.01 compared with DMSO, Dunnet’s multiple comparison test.

These data indicate that both the GFP and RFP channels provide accurate information regarding changes in autophagic flux upon drug addition. Since imaging in the GFP channel does not require photoconversion, experiments take only a fraction of the time that would otherwise be needed to track Dendra-LC3 turnover in the RFP channel. We took advantage of this fact in developing a high-throughput and high-content screening platform in Dendra2-LC3 HEK293T cells (Fig. 4*A*, Table 1). Using time-dependent reductions in GFP intensity as a readout, the assay displayed a Z’=0.52, validating this approach as a reliable primary screening modality. We then devised a layered screening scheme where hits from the primary assay were filtered based on toxicity, then subjected to a counterscreen where Dendra2-LC3 half-life is determined following photoconversion and imaging in the red channel. This organization combines the added throughput of imaging in the green channel with the ability to selectively monitor Dendra2-LC3 degradation in the red channel. Custom scripts were used to exclude toxic compounds based on their effects on cell number—this was particularly important since drugs that cause cells to die might significantly lower GFP intensity and could therefore be misconstrued as false-positives.

**Table 1:** 90/10 experiments evaluating primary high-throughput screening assay

Metric	Experiment 1 (n = 96)	Experiment 2	Experiment 3
Z'	0.51	0.49	0.56
False-positives	1	3	4
False-negatives	0	0	0
Mean DMSO-mean Torin1	0.75	1.46	1.14
DMSO SD/DMSO mean	6.40%	11%	9%

We applied this screening strategy to not only identify novel autophagy modulators but also test the ability of clinically relevant and FDA-approved drugs to affect autophagic flux. While the latter approach is unlikely to yield new chemical matter, it may nonetheless significantly accelerate translation of promising candidates into clinical trials by repurposing compounds with extensive safety testing that have been optimized for use in humans.

### Identification of novel autophagy inhibitors

To identify new autophagy modulating drugs, we performed successive screens (Fig. 4*B*) on a library of 24,000 drugs spanning considerable chemical diversity curated from the Maybridge library. We identified 2160 compounds (9%) as potential autophagy modulators from the primary screen. Of these, 1958 significantly reduced the GFP_15H_/GFP_0H_ ratio, registering as autophagy enhancers, and 202 elevated this ratio and were deemed inhibitors. Upon excluding toxic compounds and repeating the screen, 232 candidates (1%) remained as hits. Retesting reduced the number of enhancers more than tenfold while decreasing the number of inhibitors by a factor of 2.4, demonstrating a predilection toward false-enhancers in the primary screen. Following the secondary screen and retest, 23 compounds remained. Notably, all enhancers identified in the primary screen failed to pass the secondary screens (Fig. 4*B*, Table 2).

**Table 2:** Summary of Maybridge screen and orthogonal autophagic flux assays

Phase	# compounds	%	Enhancer	Inhibitor
Maybridge Library	24,000	100	NA	NA
Primary	2160	9	1958	202
Primary confirmation	232	0.97	148	84
Secondary	41	0.17	3	39
Secondary confirmation	23	0.1	0	23
Passed med chem filters	16	0.07	0	16
Retest reordered drugs	5	0.02	0	5
GFP-RFP-LC3 HeLa	5	0.02	0	5
LC3II western blot	2	0.01	0	2

Of the 23 candidate autophagy inhibitors, seven were excluded based on limited solubility and permeability, and 11 more were discarded upon acquisition of fresh powder from commercial sources. Among the five remaining autophagy inhibitors (Fig. 4*D*, Table 2), the top three candidates consistently tested among the most potent inhibitors in all assays. Additionally, each compound elicited a dramatic perinuclear accumulation of Dendra2-LC3 in HEK293T cells (Fig. 4*D*), suggestive of a late-stage block in autophagosome maturation.

We also validated the ability of these compounds to inhibit autophagy using an alternative flux assay involving HeLa cells stably overexpressing the tandem RFP-GFP-LC3 reporter (56). In this system, LC3 is fused to an acid-sensitive GFP and an acid-insensitive RFP. Upon progression from autophagosome to autolysosome, GFP fluorescence is quenched as the sensor enters an acidic environment. Application of autophagy inhibitors such as bafilomycin-A1 that inhibit lysosomal acidification results in the appearance of GFP(+)/RFP(+) (yellow) autophagosomes (Fig. S5*A*), as expected. All five of the newly identified compounds significantly increased yellow puncta accumulation in RFP-GFP-LC3 HeLa cells, indicative of effective autophagy inhibition. To assess this effect in an automated and unbiased manner, we developed an image analysis pipeline that identifies and reports the fraction of cytoplasmic GFP(+)/RFP(+) puncta (Fig. S5*B*). Using this pipeline we observed dose-dependent effects for each compound across similar concentration ranges as those observed in Dendra2-LC3 HEK293T cells (Figs. S5*C* and S6). After 12 h, all compounds except #2 had reached their maximal response (Fig. S5*D*). Compounds 1, 4, and 5 exerted more than half their maximum effect immediately after drug addition. Quinacrine, a previously reported autophagy inhibitor (57), showed similar kinetics (Fig. S5*E*), while the response of compound 3 more closely matched the kinetics of bafilomycin-A1.

We next asked whether these compounds exhibited intrinsic fluorescence that might interfere with our assessment of autophagic flux. We observed dose-dependent red fluorescence for all drugs except compound 4 (Fig. S6*A*). Even so, each compound inhibited autophagy in both flux assays at concentrations where intrinsic fluorescence is minimal (Fig. S6*B*). Similarly, all drugs exhibited green fluorescence with the exception of compound 4. Application of compound 3 also resulted in the appearance of bright perinuclear puncta that could confound estimates of GFP(+)/RFP(+) puncta in HeLa cells. Indeed, in this assay a lower concentration of compound 3 was needed to exert an effect than in the Dendra2-LC3 flux assay, which should be unaffected by changes in green fluorescence (Fig. S6*B*).

To exclude any possible contribution of autofluorescence to the measurement of flux, we assessed autophagy inhibition using a complementary, nonfluorescent assay. Treatment with compounds 1 and 2 resulted in a significant and reproducible accumulation of LC3-II by western blotting, indicative of impaired autophagosome clearance (Fig. 4, *D* and *E*). These data confirm both compounds as autophagy inhibitors; however, the intrinsic fluorescence of compound 2, and to a lesser extent compound 1, can complicate readouts of flux in fluorescence-based autophagy assays.

### Measuring the autophagy modulating effects of clinically relevant drugs

**Figure 5:**
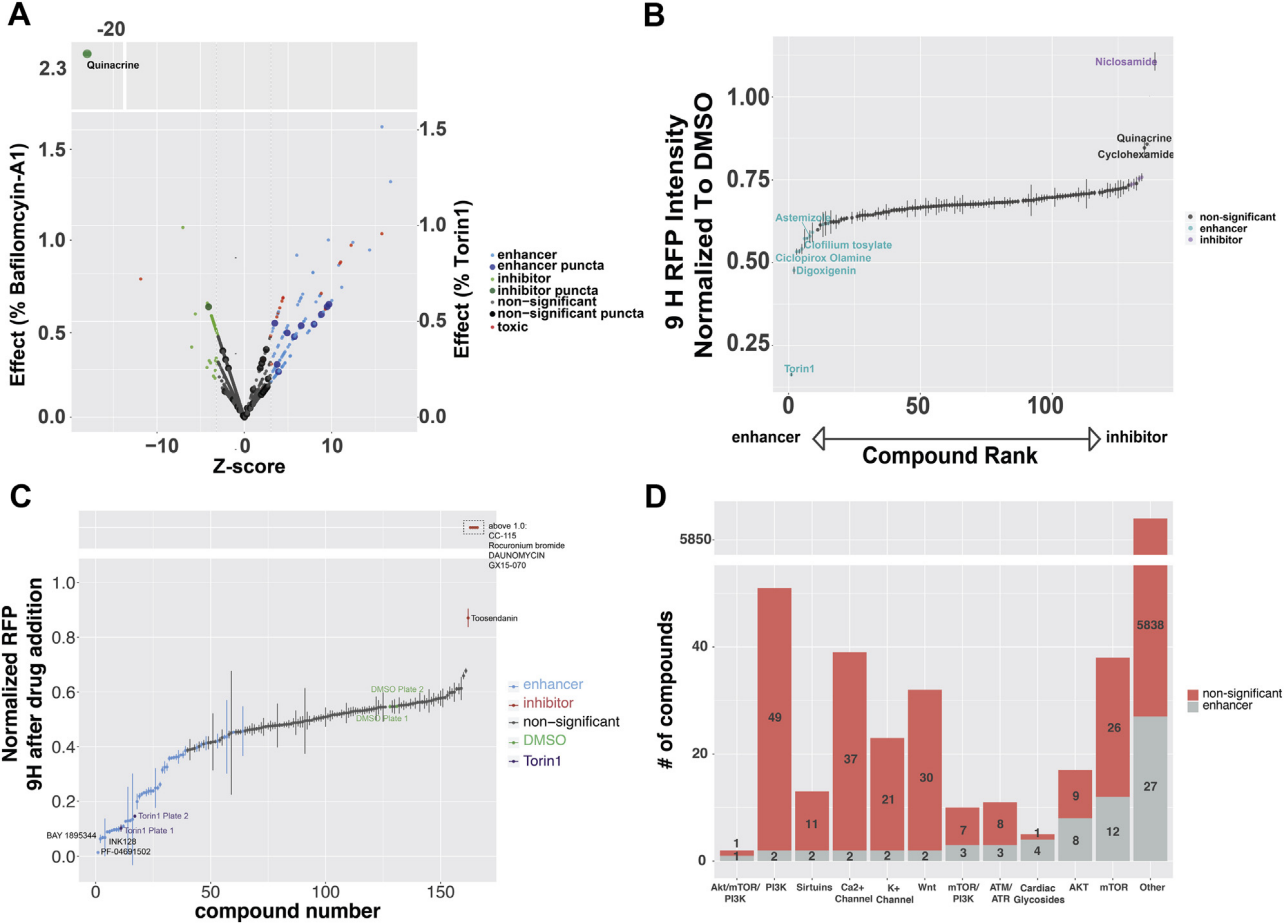
**Evaluating the autophagy modulating effects of clinically relevant drugs.***A*, primary screen of the Prestwick drug library. Z-score is calculated as fold SD_DMSO_ greater or less than mean DMSO_15H/0H_. Significant changes in flux, toxicity, or puncta for each drug are indicated by the color and size of representative *dots*, according to the key. The magnitude of effect is represented as % bafilomycin-A1 (*left y-axis*, for inhibitors) or % Torin1 (*right y-axis*, for enhancers). *B*, time-dependent decay in red (photoconverted) Dendra2-LC3 was used in a secondary screen of nontoxic candidates emerging from the primary screen. *C*, secondary screen of the SelleckChem Bioactive Compound-I library. Drugs with a normalized fluorescence intensity >1.0 were grouped together above the broken y-axis. *D*, stacked bar plot depicting enhancers (*gray*) identified from the Enzo, Prestwick, and SelleckChem drug libraries grouped by mechanism of action. *Grey bars* represent the compounds within each class with autophagy-enhancing activity. *Red bars* show all other compounds in the same class that had no observable effects. Drugs with distinct mechanisms of action, or those with incomplete or missing annotation, were labeled as “other.” Error bars in (*B* and *C*) represent SEM from six images (two images/well of three replicate wells).

To identify drugs with repurposing potential, we first screened the Prestwick drug library, a collection of 1280 off-patent small molecules, 95% of which have gained regulatory approval by the FDA, EMA, and other agencies. Eighteen compounds were filtered out due to toxicity; 17 of which would have otherwise been identified as autophagy enhancers due to their ability to significantly reduce GFP intensity. Among the remaining compounds, 129 exhibited significant effects on Dendra2-LC3 fluorescence, with 88 significantly reducing the GFP_15H_/GFP_0H_ ratio (*i.e.*, enhancing autophagy) and 41 increasing the ratio (*i.e.*, inhibiting autophagy) (Fig. 5*A*, Fig. 5-source data 1).

We also identified and counted Dendra2-LC3 puncta—corresponding to autophagosomes—in treated cells, since a change in the number or size of puncta could indicate either autophagy induction or late-stage autophagy inhibition (3). In fact, two previous high-throughput screens utilized changes in LC3 puncta number to identify autophagy modulators (58, 59). In native Dendra2-LC3 cells, we observed an increase in Dendra2-LC3 puncta number in response to 38 drugs, but only 13 of these compounds significantly affected autophagic flux in the primary screen. Among these 13, 11 reduced the GFP_15H_/GFP_0H_ ratio and were counted as enhancers, while two compounds acted as inhibitors and increased the GFP_15H_/GFP_0H_ ratio (Fig. 5*A*, Fig. 5-source data 1).

All 129 hits, along with two drugs that narrowly missed significance in the primary assay, were then evaluated for their ability to affect the degradation of photoconverted Dendra2-LC3 (Fig. 5*B*, Fig. 5-source data 1). Seventeen compounds (13%) significantly modulated autophagic flux; of these, 11 enhanced flux and six inhibited it. Two drugs, diacerin and mitoxanthrone, displayed intrinsic red fluorescence in unmodified HEK293T cells independent of their effects on autophagy (Fig. S3*B*, Fig. 5-source data 1) and were excluded from further analyses.

Among notable enhancers were the antiarrhythmic drugs digoxigenin, clofilium tosylate, and ciclopirox olamine, an off-patent antifungal agent found in both the Prestwick and Enzo libraries. Similarly, the anthelmintic niclosamide inhibited autophagy to a comparable extent as bafilomycin-A1 in the Enzo compound screen and was the strongest inhibitor in the Prestwick library, highlighting assay consistency across different libraries. As seen previously, cyclohexamide modestly stabilized photoconverted (red) Dendra2-LC3, despite lowering green Dendra2-LC3 levels in the primary screen. In keeping with the high false-positive rate observed in the Maybridge library screen, 87% of primary screen “enhancers” failed to stimulate autophagy in counterscreening, suggesting that the primary screen is sensitive but not specific for autophagy enhancers; in this context, the added selectively of the secondary screen is essential for filtering out false-positives.

We next screened 4577 compounds from the SelleckChem Bioactive Compound-I library, a structurally diverse library of medicinally active, cell-permeable compounds, many of which are FDA-approved. Following two rounds of primary screening, 161 potential autophagy modulators were further tested in secondary assays of photoconverted Dendra2-LC3 half-life. In this way, we identified five autophagy inhibitors and 45 enhancers (Fig. 5*C*, Fig. 5-source data 2)—notably, 24 of the enhancers have been tested, or are currently being tested, in clinical trials. Of these, five have received regulatory approval for various indications and ten more have demonstrated safety in phase I clinical trials (Fig. 5-source data 2). Dual mTORC1/mTORC2 ATP-competitive antagonists consistently produced the greatest enhancement of flux. Several additional compounds exhibited dual inhibition of mTOR and PI3K including (PF-04691502, INK128, Vistusertib, and LY3023414). The pronounced effects of these compounds are in keeping with those elicited by NVP-BEZ235 in the Enzo library (Fig. 3). AKT inhibitors exhibited more modest effects on flux, as did mTOR allosteric modulators such as rapamycin, everolimus, and deforolimus. While drugs targeting these pathways were overrepresented among the Enzo, Prestwick, and Selleckchem libraries, many compounds identified in our screen affected noncanonical pathways involving Wnt, CHK1, CK2, ATM/ATR, and gp130 (Fig. 5*D*, Fig. 5 source data 2).

### Identification of autophagy enhancers in human motor neurons

**Figure 6:**
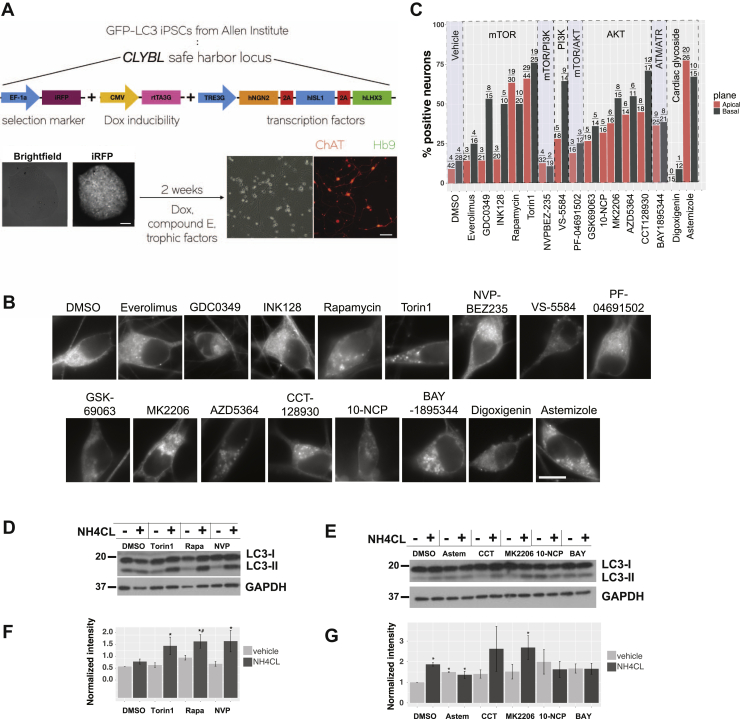
**Autophagy enhancers have mixed effects in human motor neurons.***A*, schematic describing the generation of human motor neurons differentiated from iPSCs with GFP knocked in to the *MAP1LC3B* locus. TALEN-mediated genome editing was used to introduce a DNA cassette containing the transcription factors Ngn2, Isl1, and Lhx3 downstream of a doxycycline responsive TRE3G regulatory element. Positive colonies were selected for using iRFP fluorescence. Two weeks of induction produce a homogeneous population of choline acetyl transferase (ChAT) and homeobox 9 (HB9) positive motor neurons (iMNs). Scale bars = 100 μm. *B*, day 14 iMNs imaged in the apical plane 5 h after treatment with the indicated compounds at 5 μM. Scale bar = 20 μm. *C*, percentage of neurons in the apical (*red*) and basal (*gray*) planes showing visible autophagosomes, as determined by a blinded observer. *Black numbers* above each column show the number of positive neurons over total neurons quantified, pooled from ≥3 replicates. *D* and *E*, representative western blots of Day 14 iMNs treated for 5h with 10 μM of each compound, with and without 10 mM ammonium chloride (NH4CL). *F* and *G*, quantification of western blots with intensity normalized to the mean of DMSO. Error bars represent standard error of the mean from at least three replicate experiments. ∗*p* < 0.0.05, Tukey’s posthoc test, when compared with DMSO. ^#^*p* < 0.0.05, Tukey’s posthoc test, when compared with DMSO/NH4CL.

We next measured the ability of select enhancers to modulate autophagy in human neurons. To do this, we obtained human induced pluripotent stem cells from the Allen Cell Collection in which the gene encoding GFP had been inserted immediately upstream of the *MAP1LC3B* ORF, such that endogenous LC3 is labeled in a seamless manner by GFP. Using transcription activator-like effector nucleases (TALENs) (60), we then inserted an inducible cassette into the *CLYBL* safe harbor locus of GFP-LC3 iPSCs, allowing for the regulated expression of a set of transcription factors (Lhx3, Isl1, and Ngn2) that are sufficient for the rapid and reproducible generation of ventral motor neurons from iPSCs (61) (iMNs; Fig. 6*A*). Upon imaging of EGFP-LC3 iMNs by live cell fluorescence microscopy, we observed diffuse GFP-LC3, as well as punctate structures that localized to both neuronal somata and axons (Fig. 6*B*). GFP-LC3 puncta within axons traveled in a bidirectional manner at varying speeds (Supplemental Movie 4), similar to what has been previously observed in dorsal root ganglion neurons from GFP-LC3 mice (48, 62). Since GFP-LC3 abundance and puncta formation in iMNs may indicate autophagy induction or inhibition, we tested the ability of candidate compounds to enhance flux by two complimentary metrics—the percentage of iMNs with visible autophagosomes (Fig. 6, *B* and *C*, Fig. S7*A*), and the relative abundance of LC3-II protein as measured by Western blotting, with and without lysosomal inhibition *via* ammonium chloride (Fig. 6, *D* and *E*). When quantifying autophagosomes, somata were imaged in both the apical and basal planes, as these compartments display distinct populations of autophagosomes that differ in origin, maturity, and motility (62).

Due to the limited nature of these assays, we selected a subset of compounds, each targeting distinct autophagy-related pathways, for testing in iMNs by fluorescence microscopy (Fig. 6, *B* and *C*) and Western blotting (Fig. 6, *D* and *E*). In these cells, mTOR inhibition with Torin1 and rapamycin, as well as AKT inhibition with CCT128930 and MK2206, increased the percentage of neurons with visible GFP-LC3 puncta and also increased flux as gauged by LC3-II Western blotting. Dual inhibition of mTOR and PI3K by NVP-BEZ235 likewise enhanced autophagy flux *via* LC3-II Western blotting, but had little effect on GFP-LC3 puncta in iMNs. Even so, we noted the formation of abundant, small GFP-LC3 puncta within iMN axons following NVP-BEZ235 treatment (Supplemental Movie 5), potentially indicative of a unique mechanism of autophagy activation with this compound. In contrast, the ATR inhibitor BAY1895344, the cardiac glycoside astemizole and the AKT inhibitor 10-NCP increased GFP-LC3 puncta number in treated neurons, but elicited no change in LC3-II levels after ammonium chloride application, suggesting that they block autophagy flux. The fact that both enhancers and inhibitors of autophagic flux produce an accumulation of GFP-LC3 positive autophagosomes is in keeping with our observations in Dendra2-LC3 HEK293T cells (Figs. 3*B*, 4, *D* and *E* and 5*A*).

### Enhancing autophagic flux produces distinct effects in ALS/FTD models

**Figure 7:**
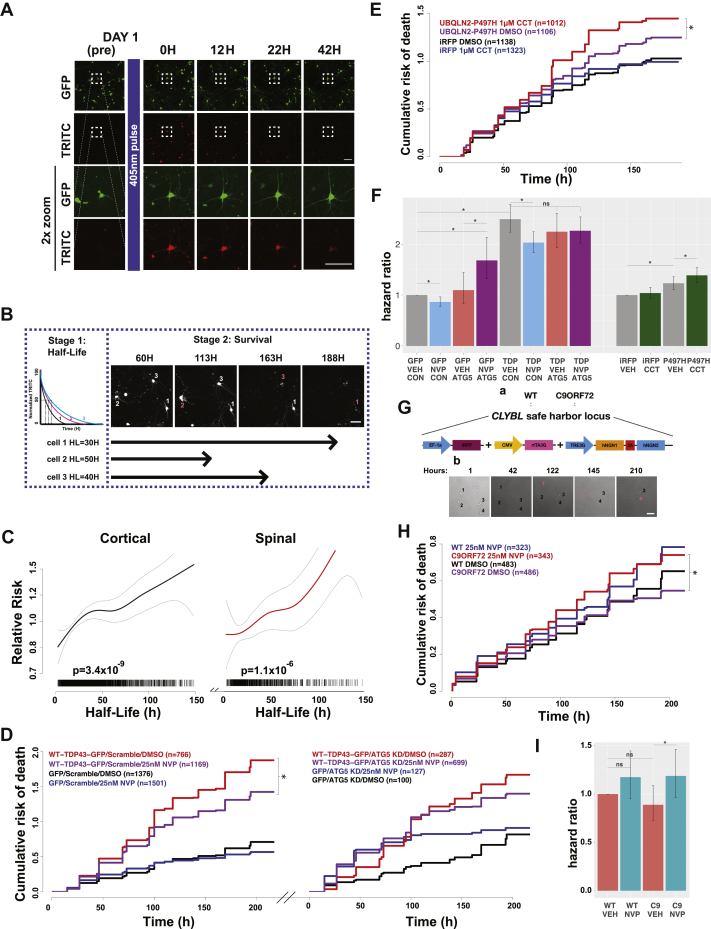
**Autophagy modulation has distinct effects in ALS/FTD disease models.***A*, mixed rodent spinal neurons were transfected on DIV 4 with Dendra2-LC3, imaged 24h later (Day 1 pre), then pulsed with 405 nm light to photoconvert Dendra2-LC3 before imaging repeatedly and longitudinally over several days to track the time-dependent loss of red fluorescence and neuronal survival. Scale bars = 100 μm in each panel. *B*, experimental outline for determining the relationship between Dendra2-LC3 half-life and neuronal survival. After calculating Dendra2-LC3 half-life for individual neurons (Stage 1), each cell is prospectively tracked using automated microscopy to determine its time of death (Stage 2; *red number* and corresponding arrow). Scale bar = 100 μm. *C*, penalized spline Cox proportional hazards model depicting Dendra2-LC3 half-life (x-axis) *versus* relative risk of death (y-axis) for primary cortical (*black*) and spinal (*red*) neurons, demonstrating a strong proportional relationship for both populations (cortical: *p* = 3.4 × 10^−9^; spinal *p* = 1.1 × 10^−6^, linear Cox proportional hazards). Each *hash mark* represents an individual neuron, collected from three biological and eight technical replicates each. *Gray dotted lines* mark 95% confidence intervals. *D*, NVP-BEZ235 (25 nM NVP) treatment suppresses toxicity in primary cortical neurons cotransfected with WT-TDP43-GFP and scramble siRNA, but not those transfected with WT-TDP43-GFP and ATG5 siRNA. Table S2 summarizes the hazard ratio and statistical significance of each comparison as determined by Cox proportional hazards analysis. N for each group represents total neurons pooled from three biological replicates. ∗*p* < 0.05, Cox proportional hazards analysis. *E*, CCT128930 (1 μM) treatment increased toxicity in primary cortical neurons overexpressing iRFP-P497H-UBQLN2. Table S3 summarizes hazard ratio and statistical significance of each comparison. *F*, hazard ratios calculated in *D* and *E*. ∗*p* < 0.05, Cox proportional hazards analysis. Error bars mark 95% confidence intervals. *G*, a, schematic depicting the generation of WT and mutant C9ORF72 iPSC-derived neurons. b, day 14 neurons were treated with the indicated compound, imaged for an additional 10 days, and time of death recorded for each neuron (*red numbers*). Scale bar = 100 μm. *H*, NVP-BEZ235 (25 nM NVP) treatment increased toxicity in mutant C9ORF72 neurons, but not WT controls. Table S5 summarizes the hazard ratios and statistical significance. N for each group represents total neurons pooled from three replicate experiments. ∗*p* < 0.05, Cox proportional hazards analysis. *I*, hazard ratios from the experiments in (*H*). Error bars denote 95% confidence intervals.

Diverging evidence suggests that autophagy induction may be protective or deleterious in ALS/FTD models (63, 64). We aimed to shed light on these conflicting reports using the autophagy-modulating compounds identified through screening in Dendra2-LC3 HEK293T cells and human iMNs. Before doing so, however, we first evaluated the relationship between basal autophagy rates and the survival of cultured primary neurons using automated microscopy (AM), a technology capable of individually tracking thousands of cells prospectively over time (65, 66). Primary rodent spinal and cortical neurons were transfected with a plasmid encoding Dendra2-LC3, photoconverted with a brief pulse of blue light, and imaged by AM (Fig. 7*A*). By measuring single-cell intensity within the TRITC channel over time, we are able to calculate a half-life for Dendra2-LC3 in individual neurons, corresponding to autophagic flux within each cell. Cortical neurons exhibited slightly higher basal rates of autophagy than spinal neurons, with a mean single-cell Dendra2-LC3 half-life of 33.2 h compared with 37.1 h seen in spinal neurons (Fig. S7, *B* and *C*, *p* = 7.1 × 10^−4^, Welch two-sample *t* test).

We also determined the life span of each neuron using custom scripts that assign a time of death for each cell (66, 67). To assess the relationship between basal rates of autophagy in neurons and their survival, we incorporated Dendra2-LC3 half-life as a continuous variable into a Cox proportional hazards model of neuronal survival (68) (Fig. 7*B*). For both cortical and spinal neurons, rapid turnover of Dendra2-LC3 was associated with extended life span (Fig. 7*C*, cortical: *p* = 3.4 × 10^−9^, spinal *p* = 1.1 × 10^−6^, Cox hazards analysis). These results indicate that higher rates of basal autophagic flux are associated with prolonged neuronal survival *in vitro*. Accordingly, we found that treating neurons with NVP-BEZ235 to be modestly neuroprotective in control neurons expressing EGFP alone (Fig. 7, *D* and *F*, Table S2, *p* = 8.4 × 10^−4^, Cox hazards analysis).

We then evaluated the effects of autophagy enhancing drugs in neuronal models of ALS/FTD involving TDP43, an RNA-binding protein whose accumulation is integrally connected with both ALS and FTD (69, 70). In prior studies, TDP43 overexpression reproduced characteristic features of disease, including the formation of ubiquitin- and TDP43-positive neuronal inclusions, cytoplasmic TDP43 mislocalization, and neurodegeneration (40, 71, 72). Supporting a beneficial role for autophagy in disease, induction of autophagy using a family of small molecules suppressed toxicity in primary neuron and human iPSC-derived neuron and astrocyte ALS/FTD models (40). NVP-BEZ235 likewise demonstrated protective effects in TDP43-overexpressing neurons (Fig. 7, *D* and *F*, Table S2; *p* = 3.39 × 10^−5^, Cox hazards analysis). Knockdown of the essential autophagy gene *ATG5* interfered with these effects, suggesting that NVP-BEZ235 rescued toxicity in an autophagy-dependent manner.

Mutations in *UBQLN2* cause X-linked ALS and ALS with dementia (37). We previously demonstrated that mutant UBQLN2 overexpression elicits dose-dependent toxicity in primary cortical neurons (73). In light of these and additional results suggesting that mutant UBQLN2 impairs the clearance of ubiquitinated proteins (74, 75, 76), we surmised that autophagy induction might rescue mutant UBQLN2-related toxicity. However, the instability of lysosomal V-ATPase subunits observed in cells expressing mutant UBQLN2 (76, 77) may create downstream blocks in autophagy flux that could potentially be exacerbated by autophagy induction, resulting in more severe toxicity. To discern between these possibilities, we monitored the survival of primary cortical neurons transfected with iRFP-tagged mutant UBQLN2 (iRFP-P497H-UBQLN2) following treatment with the autophagy inducers NVP-BEZ235 and CCT-128930. As expected, mutant UBQLN2 expression elevated risk of death relative to iRFP alone (Fig. 7, *E* and *F*, Table S3, *p* = 4.18 × 10^−5^, Cox hazards analysis). Treatment with CCT-128930 significantly increased the risk of death in mutant UBQLN2-expressing neurons (Fig. 7, *E* and *F*, Table S3, *p* = 1.5 × 10^−3^, Cox hazards analysis), and a similar trend was observed in cells treated with NVP-BEZ235 (Fig. S7, *C* and *F*, Table S3, *p* = 0.27). In contrast to data from the TDP43 ALS/FTD model (Fig. 7, *D* and *F*), these results suggest that autophagy induction in UBQLN2 ALS/FTD may enhance rather than reduce toxicity.

A hexanucleotide expansion in the first intron of the *C9ORF72* gene is the most common inherited cause of ALS and FTD (34, 35). Decreased *C9ORF72* RNA and protein is observed in tissue from C9ORF72-ALS/FTD patients (34, 78, 79, 80), and the C9ORF72 protein regulates autophagy induction (81, 82, 83), autophagosome maturation (82), and lysosomal biogenesis (83). Consequently, loss of function toxicity due to dysfunctional autophagic and endolysosomal degradation may promote neurodegeneration in *C9ORF72* mutation carriers (81, 84, 85). Although autophagy induction may be a promising therapeutic strategy (84, 85), opposing evidence suggests that *C9ORF72* mutations inhibit mTOR signaling (83, 86), increase basal autophagy rates (83), and disrupt lysosomal regulation (82, 87). To determine whether autophagy enhancement suppresses or accentuates mutant *C9ORF72*-mediated toxicity, we monitored survival of human neurons differentiated (61, 88) from *C9ORF72* mutant iPSCs (Fig. 7*G*) following treatment with NVP-BEZ235. In agreement with a previous study, we found no difference in survival between WT and mutant *C9ORF72* neurons in the absence of any additional stressors (84) (Fig. 7, *H* and *I*; Table S5). In WT neurons, treatment with NVP-BEZ235 mildly increased toxicity, but to a nonsignificant degree (Fig. 7, *H* and *I*, Table S5; *p* = 0.12), while it significantly increased the risk of death in C9ORF72 neurons (Fig. 7, *H* and *I*; Table S5; *p* = 0.01, Cox hazards analysis). These findings, together with those from UBQLN2-related ALS/FTD models (Fig. 7, *E* and *F*), indicate that the therapeutic benefit of autophagy induction is apparent in some, but not all disease contexts. Furthermore, enhancing autophagy flux may result in a counterproductive increase in toxicity in the presence of lysosomal dysfunction or other late-stage blocks in autophagosome maturation.

## Discussion

In this study we developed a unique human reporter cell line enabling the noninvasive measure of autophagic flux in living cells, without interference from pathway intermediates or potentially toxic pathway inhibitors. Building on previous efforts to isolate autophagic clearance from induction (40, 56, 89), here we created a system with the pivotal capacity to assess *native* autophagic flux, thereby avoiding several basic confounds associated with overexpression of autophagy reporters (90, 91). This is of particular relevance considering the intersection between autophagy and nutrient/energy sensing (42), the role of microtubule associated transport in autophagosome maturation (48), and the cross talk between autophagy and the ubiquitin proteasome system (44). Increasing protein dosage can also induce aberrant aggregation of misfolded proteins and influence the likelihood of protein phase separation (90, 92). Beyond the potential toxicity associated with these outcomes, overexpression of LC3 and LC3-based reporters is sufficient to produce visible puncta that could be mistaken for autophagosomes.

Through the targeted insertion of Dendra2 into the *MAP1LC3B* locus, we generated reporter cell lines in which Dendra2-LC3 expression is driven by the endogenous *MAP1LC3B* promoter. In these cells, the baseline fluorescence intensity of nonphotoconverted (green) Dendra2-LC3 reflects the steady-state balance between LC3 synthesis and degradation. We took advantage of this relationship to quickly and accurately gauge the effects of compounds that enhance or inhibit autophagy, without the need for photoconversion of Dendra2-LC3. Such an approach can be problematic in overexpression-based systems, but it provided a robust means of estimating autophagic flux on a high-throughput basis in Dendra2-LC3 HEK293T cells. Future studies employing a full bleach or photoconversion of Dendra2-LC3 could be useful for investigating autophagy regulation at the level of transcription or protein synthesis and for identifying genetic and/or pharmacologic approaches that induce autophagy at an early stage.

Previous high-throughput screens for autophagy modulators utilized the formation of LC3-positive puncta as the major criterion for autophagy induction (58, 59). Late-stage autophagy inhibition is equally effective as autophagy induction for triggering the accumulation of LC3 puncta however, complicating the discrimination between autophagy induction or inhibition using puncta formation as the sole outcome measure. For example, niclosamide was labeled as an inducer in prior studies because of its effect on LC3 puncta (59), but was in fact a strong inhibitor of flux in Dendra2-LC3 cells. Further supporting the apparent disconnect between puncta number and autophagic activity, we identified several compounds that increased Dendra2-LC3 puncta without markedly impacting Dendra2-LC3 levels (Fig. 3*F*). Reporters that judge the clearance of autophagy substrates are intrinsically more suited to gauging autophagic flux than are those that focus on pathway intermediates such as autophagosome number. Therefore we placed particular emphasis on enhancing *productive* autophagy and the measurement of autophagic flux through the use of noninvasive reporters.

In comparison to alternative methods for measuring autophagic flux, Dendra2-LC3 cells offer unique advantages. Not only do these cells afford the only means of estimating *native* autophagic flux in living cells, but they also preclude the need for overexpression of LC3 analogues, thereby avoiding many of the pitfalls that plague other approaches. For instance, while the GFP-LC3-RFP-LC3ΔG reporter (89) is likewise capable of discriminating LC3 synthesis from degradation, measurements of autophagic flux using this probe require relating the degradation rates of two proteins, one of which is an autophagy marker and substrate (GFP-LC3) and another that is untethered within the cytoplasm (RFP-LC3ΔG). Conditions that stabilize RFP-LC3ΔG without accelerating GFP-LC3 clearance could be misinterpreted as increasing flux. Supporting this notion, in a screen using the GFP-LC3-RFP-LC3ΔG reporter, MG132 was identified as an autophagy enhancer (89), while we found that MG132 instead stabilized Dendra2-LC3. Conversely, compounds such as loperamide that enhance proteolytic clearance *via* the ubiquitin-proteasome pathway (93) may be mislabeled as autophagy inhibitors because of their preferential effects on RFP-LC3ΔG.

Several drugs purported to stimulate autophagy—including trehalose (94, 95), metformin (96, 97, 98, 99), and carbamezapine (100, 101, 102)—failed to do so in our Dendra2-LC3 cell line. Despite promising results in mouse models of Huntington’s disease (94), ALS (103), and Parkinson’s disease (104), evidence that trehalose induces autophagy is variable, with some studies claiming that the drug actually inhibits flux (89, 105, 106). Likewise, the ability of carbamazepine to enhance autophagic flux and prevent neurodegeneration was based upon changes in steady-state levels of autophagy intermediates (101, 102). Our data suggest that the protective effects of these drugs may not be a result of autophagy stimulation. However, the discrepancy in findings may be due to the high concentration of these drugs used in previous studies (95, 97, 98, 99, 100, 107) relative to the 10uM dose used in our screens, as well as species and cell-type-specific differences.

Our screen of the Maybridge library suggests that uncovering novel autophagy enhancers may be considerably more challenging than inhibitors. Testing larger libraries or incorporating iterative chemical synthesis guided by structure–activity relationships (50) may be required to effectively identify new autophagy-inducing compounds. Even so, compounds 1 and 2 were repeatedly found to inhibit autophagy in Dendra2-LC3 cells, RFP-GFP-HeLa cells, and *via* immunoblotting. While these drugs hold potential for the treatment of neoplasms that rely on autophagy for survival, their potency, activity, and bioavailability could be improved through similar means.

The finding that autophagy induction suppressed TDP43 toxicity but was harmful in ALS/FTD models involving *UBQLN2* and *C9ORF72* mutations undermines the notion that autophagy enhancement is a strategy that can be broadly applied to ALS/FTD and related neurodegenerative disorders. Autophagy is a dynamic pathway that requires coordinated regulation of several critical steps; as such, increasing autophagy *induction* without an accompanying downstream increase in substrate clearance is likely to be of little therapeutic benefit and may even be maladaptive (108, 109). We found that increasing autophagy induction in primary neurons expressing a mutant form of UBQLN2 that impairs lysosomal acidification (76, 77) increased toxicity. We also observed a similar response to autophagy induction in human neurons carrying the *C9ORF72* mutation, which has previously been associated with dysfunctional lysosome/endosome maturation (82, 83, 86, 87). In addition, autophagy may play cell-type-specific roles that complicate the use of autophagy modulating compounds that act ubiquitously. For instance, loss of motor neuron autophagy accelerates disease onset in SOD1 (G93A) mutant mice, but also prolongs survival by preventing local inflammation (108).

Prior data suggest that neurons respond poorly to most autophagy-inducing stimuli, making them a particularly challenging cell type to target for therapy development (51, 62, 110). While some drugs enhanced flux in Dendra2-LC3 HEK293T cells but failed to do so in motor neurons, many compounds, including several that inhibited mTOR and AKT, enhanced flux in both cell types. Differences in the species or neuron subtype could potentially account for the discrepant findings. Consistent with this, and in contrast to observations in human iMNs, we noted a drastic reduction in the ability of mTOR inhibitors to activate autophagy in human iPSC-derived forebrain-like glutamatergic neurons (Chua *et al.*, unpublished results).

While measuring flux in Dendra2-LC3 HEK293T cells represents a considerable advancement, it is not without limitations. Chief among these is the reliance on reductions in Dendra2-LC3 signal intensity to indicate enhanced flux. For this reason, we developed automated programs to selectively remove drugs with toxic effects that might otherwise be misclassified as false-positives. Since HEK293T cells are actively dividing, compounds that merely inhibit growth rate may also be falsely identified as enhancers when measuring GFP fluorescence, necessitating the use of counterscreens involving the measurement of photoconverted (red) Dendra2-LC3 half-life to eliminate these false-positives from the final pool of candidate compounds. We imaged multiple frames/well and multiple wells to account for autofluorescence artifacts that are common in the red channel, but the use of brighter fluorophores or photoconvertible fluorescent proteins that emit in the far-red wavelengths may avoid these complications (111).

Due to the assay’s reliance on measuring fluorescence intensity, drugs that exhibit intrinsic fluorescence have the potential to confound flux measurements. In unlabeled HEK293T cells, compound 3 from the Maybridge library screen, and the drug Bim-1 accumulated within bright fluorescent perinuclear puncta with striking resemblance to puncta observed in bafilomycin-A1-treated Dendra2-LC3 HEK293T cells (Fig. S3). We therefore counterscreened all candidates in unlabeled HEK293T cells and confirmed autophagy inhibition using a nonimaging-based flux assay. Because of a similar dependence on measurements of fluorescence intensity, the RFP-GFP-LC3 flux assay suffers from the same problem in misidentifying intrinsically fluorescent drugs as inhibitors. Indeed, any fluorescence-based autophagy assay is likely to be impacted by intrinsic fluorescence, emphasizing the need to account for these effects in screening efforts.

Ideally, future studies will evaluate autophagic flux using complimentary reporters that provide valuable information on mechanism of action, in addition to magnitude and potency. Such an approach, coupled with a shift toward analyzing the productive autophagic clearance of substrates expressed at endogenous levels, promises to accelerate and facilitate the discovery of novel autophagy-modulating compounds with wide-ranging therapeutic potential.

## Experimental procedures

All reagents and equipment are listed in Table S8.

### Study approval

All rodent work was approved by the University of Michigan’s Committee on the Use and Care of Animals (UCUCA). Primary neurons were dissected from rats that were singly housed in chambers with environmental enrichment. Experiments were planned with the goal of minimizing the number of animals used. The Unit for Laboratory Animal Medicine at the University of Michigan performed all animal care and colony maintenance in accordance with the guidelines outlined in the NIH-supported Guide for the Care and Use of Laboratory Animals. Trained personnel performed euthanasia by following the Guidelines on Euthanasia of the American Veterinary Medical Association. Human neurons were differentiated from induced pluripotent stem cells, themselves reprogrammed from deidentified samples described in Tank and Figueroa-Romero *et al.*, 2018, and acquired through the University of Michigan ALS Repository. All individuals donating samples to the ALS Repository consented to deidentification and release of samples for subsequent research. Requests for tissue are evaluated on a case-by-case basis by the ALS Repository. Because there is no information pertinent to the individuals themselves and no way of tracing back samples to the patients, the use of these samples is considered to be exempt from institutional review board approval.

### HEK293T and HeLa cell culture

HEK293T Dendra2-LC3 and HeLa RFP-GFP-LC3 cells were cultured in media consisting of Dulbecco’s Modified Eagle Medium (DMEM) (GIBCO, cat #: 11995-065) supplemented with 20% fetal bovine serum, 1% GlutaMAX (GIBCO, cat #: 35050-061), and penicillin-streptomycin. For imaging experiments cells were placed in Neumo media (Cell Guidance Systems, cat #: M07-500) with SOS supplement (Cell Guidance Systems, cat #: M09-50).

### CRISPR editing

Single-guide RNA pairs (sgRNAs; Table S6) were selected using the CRISPR design tool available at http://crispr.mit.edu/. Sense and antisense oligonucleotides encoding each sgRNA (Table S6) were annealed and inserted into the BbsI site of the pX335 vector (Addgene, 42335, Depositing Lab: Dr Feng Zhang MIT), according to protocols available on the Addgene website. The HDR donor vector was synthesized in the pUCminusMCS backbone by Blue Heron Biotechnology.The donor sequence consisted of the Dendra2 ORF flanked by 400 bp of homologous sequence upstream and downstream of *MAP1LC3B* start codon. 1.25ug of pX335-Forward-sgRNA, 1.25ug of pX335-Reverse-sgRNA, and 2.5ug of the homology donor were transfected into HEK-293T cells using lipofectamine 2000 (Invitrogen, cat #: 11668019), according to the manufacturer’s suggested protocol. Cells were selected based on fluorescence and passaged to homogeneity.

### Western blotting and antibodies

HEK293T cells were lysed in RIPA buffer (Thermo, cat#: 89900) containing protease inhibitors (Millipore, cat #: 11836170001). iMNs were lysed in RIPA buffer, protease inhibitors, and phosphatase inhibitors (Millipore, cat #: 04906845001). To resolve LC3-I from LC3-II, lysates were loaded into 15% acrylamide gels and ran at 80V for at least 2 h. All other gels were 12% acrylamide. Proteins were transferred onto 0.45 μm PVDF membranes at 100 V for 2 h at 4C. Blots were blocked in Tris-buffered Saline with 0.1% Tween-20 (TBST) with 3% Bovine serum albumin (BSA). Antibodies used are summarized in Table S7. IR dye conjugated secondary antibodies from LICOR Biosciences were used and imaged on a LICOR-Odyssey.

### siRNA knockdown

HEK293T cells were plated at 60% confluency, then transfected the next day using DharmaFECT one Transfection reagent (Dharmacon, cat #: T-2001-02) and the following siRNAs from Dharmacon: ON-TARGETplus ATG5 Smartpool siRNA (cat #: L-004374-00-0005), ON-TARGETplus MAP1LC3B Smartpool siRNA (cat #: L-012846-00-0005), or nontargeting siRNA (cat #: D-001810-01-05), at a concentration of either 20 nM and 40 nM. Cells were imaged and lysates were collected 2 days after siRNA transfection.

### Primary screen

HEK293T Dendra2-LC3 cells were plated in HEK293T complete media at 1.1 × 10^5^ cells/ml on ViewPlate 384w plates (PerkinElmer, cat #: 6007460) using a Multidrop Combi (Thermo Scientific, cat #: 5840300), adding 50uL to each well. Approximately 48 h later, and immediately prior to imaging, HEK293T complete media was exchanged with Neumo+SOS media using a Biomek FX^P^ laboratory automation workstation (Beckman Coulter). To avoid dislodging cells during the media exchange, 35uL of the HEK293T media was removed and replaced with 45uL Neumo+SOS. Another 45uL was removed and replaced with Neumo+SOS, effectively diluting out the concentration of HEK293T complete media to 6.25%. Cells were imaged with an ImageXpress Micro (Molecular Devices) equipped with a 20× objective lens. After imaging in the GFP channel (Semrock, FITC-3540B-NTE-ZERO filter) to measure baseline fluorescence intensity, compounds were added using a BioMek FX pintool (Beckman Coulter) at a concentration of 10 μM, with the exception of the positive control Torin1 (Tocris, cat #: 4247), which was added at 1 μM. Plates were imaged again 15h after drug addition. One image was acquired per well. Images were background subtracted using FIJI (https://fiji.sc/) with a rolling ball radius of 150. Mean GFP fluorescence intensity was calculated on a whole well basis. Enhancers are defined as: [Sample_15H GFP/0H GFP_] < [DMSO_15H GFP/0H GFP_ - 3SD_DMSO_] and inhibitors as: [Sample_15H GFP/0H GFP_] > [DMSO_15H GFP/0H GFP_ + 3SD_DMSO_]. Using a custom FIJI script that measured the area of occupied by cells, toxic compounds were identified as those that elicited a reduction in cellular area ≥ 3SD, verified by eye.

### Secondary screen

Plating of HEK293T Dendra2-LC3 cells and media exchange were performed as in the primary screen, described above. Two sites per well were imaged in the Texas Red (RFP) (Semrock TxRed-4040C-NTE-ZERO filter) channel prior to photoconversion to establish background fluorescence levels. Photoconversion was accomplished using a 4s DAPI (Semrock Brightline DAPI-5060-NTE-ZERO filter) exposure, and afterward the cells were immediately imaged once more in the RFP channel. The plate was then removed from the ImageXpress stage and compounds were added to three replicate wells *via* the BioMekFX automation station at a working concentration of 10 μM. The plate was then returned to the ImageXpress and imaged every 1.5 h in a recurring loop for 16h, while maintaining 5% CO_2_, humidity, and a temperature of 37C. Initial optimization studies demonstrated a maximal Z’ = 0.79 within 9h of drug addition, and therefore this time was selected for measuring autophagic flux. We defined enhancers and inhibitors by the following criteria: enhancer, [Sample_9H RFP/(Postconversion RFP − background RFP)_] < [DMSO_9H RFP/(Postconversion RFP − background RFP)_ − 3SD_DMSO_]; inhibitor, [Sample_9H RFP/(Postconversion RFP − background RFP)_] > [DMSO_9H RFP/(Postconversion RFP − background RFP)_ + 3SD_DMSO_]. Images with autofluorescent artifacts were excluded and the remaining images were averaged on a per compound basis.

### Maybridge library screening

After exclusion of toxic compounds, all hits from the primary screen (utilizing time-dependent changes in the GFP intensity) were retested a second time using the same assay to reduce the number of potential false-positives. Hits were then confirmed by counterscreening twice with the lower-throughput secondary screen (involving clearance of photoconverted Dendra2-LC3), and promising candidates were filtered further based on their solubility and permeability. Finally, the intrinsic fluorescence of each compound was assessed in unmodified HEK239T cells, and all remaining hits were validated using orthogonal flux assays (Fig. 4*B*).

### RFP-GFP-LC3 assay

HeLa RFP-GFP-LC3 cells (56) were plated at 80% confluency in HEK293T complete media. Prior to imaging, cells were switched to Neumo+SOS media. Cells were imaged at baseline as well as 0, 4, 8, and 12h after drug addition. Images were background subtracted using the rolling ball background subtraction plugin in FIJI, with a radius = 150. LC3 puncta were identified and quantified using CellProfiler 3.0 (112) (https://cellprofiler.org/) utilizing a customized pipeline (https://github.com/BarmadaLab/LC3-puncta). Briefly, a series of image processing operations are performed to segment a cell into nuclear and cytoplasmic compartments. The contrast between puncta and background is further enhanced to emphasize LC3 puncta in both GFP and RFP images. Object-oriented colocalization then records the number of cytoplasmic red puncta that are also green, representing autophagosomes.

### Primary neuron survival assay

Primary neurons were dissected from the cortex of embryonic day 20 rat pups and plated at a density of 1 × 10^5^ cells/well on a laminin/poly-D-lysine coated 96 well plate (67, 72, 113) in Neumo complete media. Transfection and longitudinal microscopy were performed as previously described (66). Briefly, DIV4 neurons were transfected using lipofectamine 2000. Neurons were incubated with Lipofectamine-DNA mixtures for 20 min followed by washes with Neurobasal media containing 1 mM kynurenic acid to remove residual lipofectamine. Neurons were then placed in half-conditioned media and half-fresh Neumo+SOS media. An Eclipse Ti inverted microscope (Nikon) equipped with PerfectFocus, Semrock GFP, and TRITC filters, Lambda XL lamp (Sutter Instruments), and an Andor Zyla 4.2(+) sCMOS camera (Oxford Instruments) was used to acquire images at 20×. To maintain a temperature of 37C and 5% CO2 levels, the microscope was encased in a custom-built plexiglass environmental chamber. Automated stage movements, filter turret rotation, and image acquisition were controlled *via* μManager with original code written in BeanShell. In-house software was used to assign a barcode for each neuron, measure its fluorescent intensity, and register time of death as described previously (65, 66, 67, 73, 114). For optical pulse labeling experiments, a 1.5 s pulse of 405 nm light was used for photoconversion. For studies relating the rate of Dendra2-LC3 turnover to neuronal survival, only cells that lived the entire duration of OPL imaging were included in the survival analysis.

### Generation and maintenance of iPSCs

The reprogramming of iPSCs was performed as previously reported (115). iPSCs were maintained in TeSR-E8 (Stemcell Technologies, cat #: 05990) on vitronectin (Gibco, cat #: A14700) coated plates and passaged using 0.5 mM EDTA every 5 to 6 days. Lines were tested for *mycoplasma* on a monthly basis.

### Integration of transcription factors into iPSCs

Both the Ngn1/Ngn2 and Ngn2/Isl1/Lhx3 cassettes were integrated into the *CLYBL* safe harbor locus as previously described (115). Briefly, 2.5 μg of donor DNA (pUCM-CLYBL-Ngn1-Ngn2-RFP or pUCM-CLYBL-hNgn2,Il1,Lhx3-iRFP) and 1.25 μg of each targeting construct (pTLC13-L1 and pLTC13-R1) were transfected into iPSCs using Lipofectamine Stem (Invitrogen, cat #: STEM00003) according to the manufacturer’s instructions. Edited cells were iteratively screened for fluorescence and enriched to 100% fluorescent colonies, which were expanded and frozen down for future use. Targeting constructs pUCM-CLYBL-Ngn1-Ngn2-RFP and pUCM-CLYBL-hNgn2,Il1,Lhx3-RFP, from which pUCM-CLYBL-hNgn2,Il1,Lhx3-iRFP was derived, were gifts from Dr Michael Ward (National Institutes of Health).

### iNeuron differentiation and survival assay

To ensure consistency between experiments, iNeurons were generated by first freezing large batches of neural precursors (NPs) and later differentiating NPs into neurons. iNeuron IPSCs were converted into NPs by incubation with 2 μg/ml doxycycline (Sigma, cat #: D3447) in TeSR-E8 for 3 days. NPs were dissociated with Accutase (Sigma, cat#: A6964) for 5 min, pelleted by centrifugation at 200*g* for 5 min, resuspended in TeSR-E8+10% DMSO, and then frozen and stored in liquid nitrogen. Prior to thawing and plating NPs, each well of TPP-96 well plates (Midsci, cat #: TP92696) was coated overnight with 150 μl of 0.2% Polyethyleneimine (PEI) solution (Sigma, cat#: P3143) prepared in 0.1 M borate buffer (3.1 g boric acid (Fisher Chemical, cat #: A73-500) and 4.75 g sodium tetraborate (Sigma, cat #: 221732) per liter of water, pH = 8.4). PEI was removed and the plate was washed with sterile water 3× to remove residual PEI. Water was aspirated and the plate further air-dried for 1h. 150 μl of 10 μg/ml laminin (Sigma, cat#: L2020) in DMEM/F12 (GIBCO, cat#: 11320033) was added to each well and the plate was incubated at 37C for 2h, and the laminin was removed immediately before plating NPs. Frozen NPs were thawed and plated in TeSR-E8+ROCK inhibitor Y-27632 (Fisher, cat#: BDB562822) at a density of 4000 cells/well (20,000 cells/ml) and incubated overnight. Day 1-media was changed to N2 media (TeSR-E8+ 2 mg/ml doxycycline (Sigma D3447), 10 ng/ml BDNF (Peprotech, cat#: 450-02), 10 ng/ml NT3 (Peprotech, cat#: 450-03), 0.2 μg/ml laminin (Sigma, cat#: L2020), 1× N2 Supplement (Gibco, cat#: 17502-048), 1× NEAA Supplement (Gibco, cat#: 11140-050)). Day 2-media was exchanged for transition media (half TeSR-E8 and half DMEM/F12+ 2 mg/ml doxycycline, 10 ng/ml BDNF, 10 ng/ml NT3, 0.2 μg/ml laminin, 1× N2 Supplement, 1× NEAA Supplement). Day 3-media was exchanged with B27 media (Neurobasal-A (Gibco, cat#: 12349-015) + 2 mg/ml doxycycline, 10 ng/ml BDNF, 10 ng/ml NT3, 0.2 μg/ml laminin, 1× Glutamax Supplement, and 1× Culture One (Gibco, cat#: A33202-01)). Day 6-100 μl/well of B27 media without Culture One was added. Day 8-Cells were imaged in brightfield once prior to drug treatment to document any potential treatment-related toxicity. Half of B27 media was removed and replaced with Brainphys complete media (Brainphys, Stemcell Technologies, cat#: 05790, 1× SM1 supplement, cat# 05711, and 1× N2 supplement) with either DMSO or 25 nM NVP-BEZ235 (SelleckChem, cat#: S1009). iNeurons were imaged daily for 8 to 9 days *via* automated microscopy. Time of death of each neuron in at least six wells per experiment was manually recorded by an observer blinded to treatment condition.

### iMN differentiation

iMN precursors were generated, frozen, and thawed as described above for iNeuron precursors and plated on PEI/laminin coated plates. For iMN immunoblotting experiments, iMN precursors were plated at a density of 2.5 × 10^5^ cells/well in 6-well plates (1.25 × 10^5^ cells/ml). For imaging experiments iMN precursors were plated at a density of 7.5 × 10^3^ cells/well (2.14 × 10^4^ cells/ml) in 8-well hydrophobic polymer μ-slides (3, cat#: 80821). Day 1: Media change into Day 1 media (TeSR-E8, 1× N2 Supplement, 2 mg/ml doxycycline, 0.2 μg/ml Compound E (Millipore, cat#:565790)). Day 3: Media change into Day 3 media (DMEM/F12, 1× N2 Supplement, 1× nonessential amino acids, 1× Glutamax, 2 mg/ml doxycycline, 0.2 μg/ml Compound E). Day 6: Media change into B27 media (Indicator free Neurobasal-A + 2 mg/ml doxycycline, 10 ng/ml BDNF, 10 ng/ml NT3, 0.2 μg/ml laminin, 1× Glutamax Supplement, and 1× Culture One). Day 10: Addition of 1 ml of B27 media to each well of 6-well plates and 100 μl of B27 media to each well of 8-well chamber slides.

### Imaging GFP-LC3 autophagosomes in iMNs

Day 14: iMNs were imaged in brightfield to assess health prior to drug treatment. iMNs were treated with 5 μM of each compound (listed in Table S8) with the exception of Torin1, which was added at 250 nM. 5h later iMNs were imaged using an ONI Nanoimager (Oxford Nanoimaging), equipped with a 1.4NA 100× objective and sCMOS camera. Images were acquired in both the apical and basal planes for each neuron using a 75 ms exposure. For each treatment condition neurons were pooled between at least three replicate experiments.

### Statistical analyses

Statistical analyses were performed in an R and Prism 7 (Graphpad). Two-way ANOVA with Tukey’s post-test was used to identify significant changes between fluorescence intensity at multiple time points between treatment groups (Figs. 2 and 3, Figs. S3 and S4). One-way ANOVAs with Tukey’s post-hoc test was used to quantify significant differences in protein abundance on western blots (Figs. 5 and 6). The publicly available R package “survival” (https://cran.r-project.org/web/packages/survival/index.html) was used to calculate hazard ratios comparing the relative survival of neuronal populations *via* Cox proportional hazards analysis and to compare the relative risk of death for cortical and spinal neurons with varying half-lives with penalized spline regression analysis. Single-cell Dendra2-LC3 half-life was calculated by fitting the change in photoconverted Dendra2 fluorescence to a first-order exponential decay curve.

## Data availability

All data described are available in the article or its supplemental material.

## Supporting information

This article contains supporting information.

## Conflict of interest

Nathaniel Safren, Elizabeth M. Tank, and Sami J. Barmada are coapplicants on a patent application for an assay probing mammalian autophagy (US Pat Appl 16/288802).
