# Supplementary material for: Development of a specific live-cell assay for native autophagic flux
Source: J Biol Chem. 2021 Jul 23;297(3):101003. doi: 10.1016/j.jbc.2021.101003 (PMC8368035; doi:10.1016/j.jbc.2021.101003)
Supplement: Supplemental Figures S1–S7, Tables S1–S8, Fig. 3-source data and Fig. 5-source data 1 [file mmc1.pdf]

Supplemental material:

<u>Figure Captions:</u>

Figure S1: Validation of Dendra2-LC3 HEK293T cells.

(**A**) Schematic of the *MAP1LC3B (LC3)* locus, with untranslated regions (UTRs) in white, exons in blue, and introns as lines. The *Dendra2* open reading frame (ORF) was inserted just downstream of the *LC3* 5'UTR and start codon in exon 1, and sequenced verified in two separate HEK293T cell lines. (**B**) HEK293T cells were transfected with 20nM and 40nM ATG5 siRNA, or 40nM non-targeting siRNA. Lysates were collected 2d later and subject to Western blotting with anti-ATG5 and GAPDH antibodies. Relative to scramble siRNA, 20nM and 40nM ATG5 siRNA produced a 58% and 64% knockdown of ATG5 protein, respectively. ATG5 levels were normalized to GAPDH.

Figure S2: Aggregation of Dendra2 tagged proteins causes a supraphysiological increase in signal.

Primary cortical neurons were co-transfected on DIV4 with plasmids encoding EGFP (blue) and a Dendra2-tagged fragment of mutant huntingtin (HTT) carrying a pathologic expansion of 74 glutamine residues (Dendra2-HTT-exon1-Q74)[115]. 24h after transfection, Dendra2-HTT-exon1-Q74 was photoconverted with a 1s pulse of 405nm light and subsequently imaged via automated microscopy. The decay in TRITC (red) intensity was measured every hour as a metric for protein degradation. The TRITC intensity spikes upon aggregate formation, preventing accurate measurements of protein half-life. Individual lines correspond to specific cells, labeled in white in the image panels. Red cell labels indicate aggregate formation. Scale bar = 50μm

Figure S3: Off-target contributions by proteasomal inhibition and autofluorescence can compromise the specificity of autophagy flux estimates in Dendra2-LC3 cells.

(**A**) The extent of Dendra2-LC3 stabilization after treatment with DMSO, 20nM Bafilomycin-A1, 20μM MDL-28170, 100nM Bortezomib, 20nM Bafilomycin-A1 + 20μM MDL-28170, and 20nM Bafilomycin-A1 + 100nM Bortezomib was quantified in photoconverted Dendra2-LC3 HEK cells. Fluorescence is normalized to background-subtracted post-conversion intensity. Error bars signify SEM from three replicate

experiments. *=p<.01, Tukey's posthoc test relative to DMSO at 11H timepoint. (**B**) Unmodified HEK293T cells imaged in the RFP channel 9h after drug treatment. Scale bar = 50μm.

Figure S4: Proportional and bidirectional effects of autophagy modulators highlight assay sensitivity.

(**A**) Dendra2-LC3 HEK293T cells were treated with increasing concentrations of Torin1 and bafilomycin-A1. Representative images in the GFP (top) and RFP (bottom) channels, pseudocolored to accentuate intensity variations. Scale bar = 100μm. (**B**) Dendra2-LC3 clearance increased in a dose-dependent manner with Torin1 (**B**), while bafilomycin-A1 resulted in dose-dependent prolongation of Dendra2-LC3 half-life (**C**). These changes are even more apparent in the GFP channel for both Torin1 (**D**) and bafilomycin-A1 (**E**). Error bars represent SEM from 8 replicate wells. For (B-E), *$p<0.05$ using DMSO as reference group with Dunnett's multiple comparisons test. Superscript number indicates the first time point when significance ($p<0.05$) was achieved. (**F**) Dose-response curves for Torin1, NVP-BEZ235, rapamycin, and bafilomycin-A1. For autophagy enhancers, the minimal RFP intensity 7h after drug treatment relative to DMSO was set to 1, and the maximal value set to 0. For inhibitors, the maximum effect represents the maximal RFP intensity within 7h after drug treatment. Dose-response was determined similarly for the GFP channel, utilizing values 14h after drug treatment. Concentration is plotted in nM on a log(2) scale, with ≥ 3 replicate wells for each channel shown as colored dots. EC50 and IC50 values are reported along the x-axis for both RFP and GFP.

Figure S5: independent measures of autophagy confirm inhibition of autophagic flux by candidate compounds.

(**A**) HeLa cells expressing a tandem LC3 reporter (RFP-GFP-LC3) were treated with the indicated compounds and imaged 12h later in the RFP (middle panels) and GFP (bottom panels) channels. Composite images are displayed on the top row. Concentrations for each compound (3nM bafilomycin-A1; 1μM Quinacrine; 50μM 245536; and 100μM of compounds 1-5) correspond to the lowest dose resulting in the maximum degree of colocalization between GFP and RFP puncta as calculated in (C). Scale bar: 50μm (**B**) The percentage of RFP(+)/GFP(+) puncta was determined using CellProfiler[116]. Images from the GFP (1a) and RFP (1b) channels are uploaded into CellProfiler, nuclei

are identified using the GFP channel (2a-green outlines) and a nuclear mask (2b) is generated. Nuclei that do not pass size or intensity thresholds, or are on the edges of an image, are excluded (2a-purple outlines). Cellular boundaries are identified (3a-purple outlines), and the nuclear mask is subtracted from the newly created cellular mask to produce a cytoplasmic mask (3b). The intensity of cytoplasmic GFP (4a) and RFP (5a) puncta are enhanced, allowing the generation of masks corresponding to puncta in both channels (4b, 5b) and the identification of GFP(+)/RFP(+) autophagosomes (6a, b). Scale bar: 50μm. (**C**) Dose response curves showing the degree of autophagy inhibition with increasing drug concentrations, plotted on a log2 scale. The y-axis represents the proportion of RFP(+) puncta that are GFP(+), with the maximum and minimum set to 1.0 and 0, respectively. Dots represent individual technical replicates. (**D**) RFP-GFP-LC3 HeLa cells were imaged, treated with the lowest dose that produced the maximum response as calculated in (C), then imaged again 0, 4, 8 and 12h after drug treatment. (**E**) Normalized data from (D), depicting the time to maximal effect for each compound. Data in (D, E) represent mean ± SEM from 3 biological replicates, 8 technical replicates each.

Figure S6: Intrinsic fluorescence confounds flux estimates in a subset of Maybridge library hits

Unmodified HEK293T cells were treated with each compound that registered as an autophagy inhibitor in the screen of the Maybridge 24K library (Fig. 4) Images were acquired in the GFP and RFP channels 9h after treatment of each drug at either 10μM or 80μM. Images are psuedocolored to accentuate intensity differences. For compounds 1 and 5, the contrast settings for the 80μM images were optimized to emphasize low levels of intrinsic fluorescence. All other images are presented at equivalent brightness and contrast. Scale Bar=50μm (**B**) Dose response relationships for each drug in the Dendra-LC3 HEK293T (black), HeLa RFP-GFP-LC3 (blue) autophagic flux assays, as well as intrinsic fluorescence in the GFP (green) and RFP (red) channels in unmodified HEK293T cells. In Dendra2-LC3 HEK293T cells the maximum effect represents the greatest increase in RFP intensity 14h after drug treatment. In RFP-GFP-LC3 HeLa cells the effect represents the proportion RFP(+) puncta that are GFP(+), with the maximum and minimum set to 1.0 and 0, respectively. For unlabeled HEK293T cells the maximum fluorescence intensity observed 9h after drug treatment was set to 1 and the lowest to 0. Concentration is plotted in nM on a log(2) scale, with ≥3 replicate wells for each

concentration shown as colored dots. Dotted vertical lines correspond to 10μM, which is the concentration the compounds were initially screened at.

Figure S7: Autophagy modulating drugs elicit varied effects on GFP-LC3 puncta formation and primary cortical and spinal neurons display subtly different rates of autophagic flux.

(**A**) Further stratification of Fig. 6C, where LC3-GFP puncta were quantified in day 14 iMNs following 5 hours of treatment with the corresponding compounds. Those cells with more than 10 puncta are labeled in black and those with fewer than 10 puncta in red. (**B**) Histogram depicting single cell Dendra2-LC3 half-lives in cortical and spinal neurons. Relative to spinal neurons (n=1043), a leftward shift was observed in the Dendra2-LC3 half-life distribution of cortical neurons (n=4058) indicating a greater frequency of cortical neurons exhibiting high rates of autophagic flux compared to spinal neurons ($p=8.2x10^{-4}$, two-sample Kolmogorov-Smirnov (KS) test*).* (**C**) Consistent with this, the mean single-cell Dendra2-LC3 half-life was slightly reduced in cortical neurons (33.2h) compared to spinal neurons (37.1h) ($p=7.1x10^{-4}$, Welch two sample t-test), indicating higher rates of basal autophagy in cortical neurons. (**D**) NVP-BEZ235 (25nM) treatment produced a trend towards an improvement in the survival of primary cortical neurons expressing iRFP alone, and a trend towards increased toxicity in neurons expressing iRFP-P497H-UBQLN2. Neither comparison reached statistical significance. Table S4 summarizes hazard ratio and statistical significance of each comparison.

Supporting Movie Captions

Supplemental Movie 1: Dendra2-LC3 HEK293T cells treated with DMSO.

Dendra2-LC3 HEK293T cells imaged 6h after treatment with DMSO, 3x actual speed. Scale bar = 10μm

Supplemental Movie 2: Dendra2-LC3 HEK293T cells treated with Torin1.

Dendra2-LC3 HEK293T cells imaged 6h after treatment with 1μM Torin1, 3x actual speed. Scale bar = 10μm

Supplemental Movie 3: Dendra2-LC3 HEK293T cells treated with Bafilomycin-A1.

Dendra2-LC3 HEK293T cells imaged 6h after treatment with 20nM Bafilomycin-A1, 3x actual speed. Scale bar = 10μm

Supplemental Movie 4: Axonal transport of GFP-LC3 in iMNs. Day 14 GFP-LC3 iMNs imaged 5h after treatment with 5μM AZD5364, actual speed. Scale bar = 10μm

Supplemental Movie 5: Axonal transport of GFP-LC3 in iMNs. Day 14 GFP-LC3 iMNs imaged 5 h after treatment with 5μM NVP-BEZ235, 3x actual speed. Scale bar = 10μm

Table S1: Summary of Two-Way ANOVA analyses accompanying Figure 2

Figure	component	Significant	P-value
2B	Interaction	F (90,248) = 15.09	P<.0001
2B	Time	F (30,248) = 96.03	P<.0001
2B	Treatment	F (3,248) = 1753	P<.0001
2C	Interaction	F (140,348) = 24.92	P<.0001
2C	Time	F (28,348) = 2422	P<.0001
2C	Treatment	F (5,348)= 3828	P<.0001
2D	Interaction	F (90,248) = 67.45	P<.0001
2D	Time	F (30,248) = 74.01	P<.0001
2D	Treatment	F (3,248) = 5951	P<.0001
2E	Interaction	F (140,348) = 273	P<.0001
2E	Time	F (28,348) = 222.7	P<.0001
2E	Treatment	F (5,348)= 21726	P<.0001

Table S2: Cox proprotional hazards analysis comparing TDP43 expressing primary neurons treated with NVP-BEZ235 or vehicle

group	N	hazard ratio	lower 95%	upper 95%	p-value
GFP/Scramble/DMSO	1376	1	1	1	1
GFP/Scramble/25nM NVP	1501	0.8658	0.7779	0.9637	0.0083513
GFP/ATG5 KD/DMSO	100	1.0974	0.832	1.4475	0.5106564
GFP/ATG5 KD/25nM NVP	127	1.6811	1.3265	2.1303	1.72E-05
WT-TDP43-GFP/Scramble/DMSO	766	2.4925	2.2382	2.7758	4.12E-62
WT-TDP43-GFP/Scramble/25nM NVP	1169	2.0319	1.8391	2.2449	3.74E-44
WT-TDP43-GFP/ATG5 KD/DMSO	287	2.2457	1.9353	2.6058	1.55E-26
WT-TDP43-GFP/ATG5 KD/25nM NVP	699	2.2639	2.0209	2.5362	3.75E-45

group	N	hazard ratio	lower 95%	upper 95%	p-value
WT-TDP43-GFP/Scramble/DMSO	766	1	1	1	1
WT-TDP43-GFP/Scramble/25nM NVP	1169	0.8116	0.7333	0.8983	5.54E-05
WT-TDP43-GFP/ATG5 KD/DMSO	287	0.8963	0.7716	1.041	0.1516617
WT-TDP43-GFP/ATG5 KD/25nM NVP	699	0.9062	0.8076	1.0167	0.0934768

group	N	hazard ratio	lower 95%	upper 95%	p-value
GFP/ATG5 KD/DMSO	100	1	1	1	1
GFP/ATG5 KD/25nM NVP	127	1.5133	1.0676	2.1452	0.0199555
WT-TDP43-GFP/ATG5 KD/DMSO	287	1.9916	1.4804	2.6795	5.32E-06
WT-TDP43-GFP/ATG5 KD/25nM NVP	699	2.0121	1.5199	2.6636	1.03E-06

group	N	hazard ratio	lower 95%	upper 95%	p-value
WT-TDP43-GFP/Scramble/DMSO	766	1	1	1	1
WT-TDP43-GFP/Scramble/25nM NVP	1169	0.8068	0.7289	0.893	3.39E-05

group	N	hazard ratio	lower 95%	upper 95%	p-value
WT-TDP43-GFP/ATG5 KD/DMSO	287	1	1	1	1
WT-TDP43-GFP/ATG5 KD/25nM NVP	699	1.0115	0.8668	1.1803	0.8849334

Table S3: Cox proportional hazards analysis for iRFP-UBQLN2 expressing primary neurions treated with CCT128930 or vehicle

group	N	hazard ratio	lower 95%	upper 95%	p-value
iRFP DMSO	1138	1	1	1	1
iRFP 1uM CCT	1323	1.0393	0.9383	1.1511	0.4597729
UBQLN2-P497H DMSO	1106	1.2333	1.1156	1.3634	4.18E-05
UBQLN2-P497H 1uM CCT	1012	1.3877	1.2516	1.5385	4.88E-10

group	N	hazard ratio	lower 95%	upper 95%	p-value
UBQLN2-P497H DMSO	1106	1	1	1	1
UBQLN2-P497H 1uM CCT	1012	1.1839	1.0666	1.3142	0.0015221

Table S4: Cox proportional hazards analysis in iRFP-UBQLN2 expressing primary neurons treated with NVP-BEZ235 or vehicle

group	N	hazard ratio	lower 95%	upper 95%	p-value
iRFP DMSO	1138	1	1	1	1
iRFP 25nM NVP	713	0.9111	0.8106	1.0242	0.1189461
UBQLN2-P497H DMSO	1106	1.2238	1.1071	1.3528	7.83E-05
UBQLN2-P497H 25nM NVP	585	1.3077	1.1623	1.4713	8.18E-06

UBQLN2-P497H DMSO as reference

group	N	hazard ratio	lower 95%	upper 95%	p-value
UBQLN2-P497H DMSO	1106	1	1	1	1
UBQLN2-P497H 25nM NVP	585	1.0686	0.9498	1.2022	0.270139

Table S5: Cox proportional hazards analysis in WT and mutant C9ORF72 iNeurons treated with NVP-BEZ235 or vehicle.

group	N	hazard ratio	lower 95%	upper 95%	p-value
WT DMSO	483	1	1	1	1
WT 25nM NVP	323	1.1751	0.9546	1.4464	0.1280727
C9ORF72 DMSO	486	0.8888	0.7266	1.0872	0.2516026
C9ORF72 25nM NVP	343	1.1877	0.9654	1.4613	0.1038034

group	N	hazard ratio	lower 95%	upper 95%	p-value
C9ORF72 DMSO	486	1	1	1	1
C9ORF72 25nM NVP	343	1.3184	1.0676	1.6281	0.0102287

Table S6: Sense and antisense oligonucleotides cloned into pX335 plasmid

oligo	sequence
LC3bD2_Forward_Sense	CAC CGT TCG GTG AGT GTC GCC GCG A
LC3bD2_Forward_Antisense	AAA CTC GCG GCG ACA CTC ACC GAA C
LC3bD2_Reverse_Sense	CAC CGT TCT CCG ACG GCA TGG TGC A
LC3bD2_Reverse_Antisense	AAA CTG CAC CAT GCC GTC GGA GAA C

Table S7: Antibodies used in this study

Antibody	dilution	manufacturer	Cat #
Rabbit anti-LC3	1:1000	Cell Signaling	2775S
Rabbit anti-ATG5	1:1000	Cell Signaling	129945
Mouse anti-GAPDH	1:1000	Millipore	MAB374
Donkey anti-Mouse iRDye 680RD	1:10,000	LICOR	926-68072
Donkey anti-Rabbit iRDye 800CW	1:10,000	LICOR	926-32213

Table S8: List of Reagents and equipment used

Reagent	Catalog number	Manufacturer	Manufacturer location
**Compounds**			
Bafilomycin-A1	B1793	Sigma	St. Louis, MO
Rapamycin	13346	Cayman Chemical	Ann Arbor, MI
AKT Inhibitor X	14863	Cayman Chemical	Ann Arbor, MI
Astemizole	16967	Cayman Chemical	Ann Arbor, MI
LY294002	70920	Cayman Chemical	Ann Arbor, MI
Quinacrine (hydrocloride hydrate)	15041	Cayman Chemical	Ann Arbor, MI
M 344	13174	Cayman Chemical	Ann Arbor, MI
BAY-1895344	26536	Cayman	Ann Arbor, MI
MK2206 hydrochloride	11593	Cayman	Ann Arbor, MI
CCT128930	18194	Cayman	Ann Arbor, MI
NVP-BEZ235	S1009	SelleckChem	Houston, TX
**Media Components**			
DMEM	11995-065	Gibco	Waltham, MA
FBS	10438026	Fisher	Fair Lawn, NJ
GlutaMAX	35050-061	Gibco	Waltham, MA
penicillin-streptomycin			
Neumo media	M07-500	Cell Guidance Systems	Cambridge, UK
SOS supplement	M09-50	Cell Guidance Systems	Cambridge, UK
laminin	L2020	Sigma	St. Louis, MO
DMEM/F12	11320033	Gibco	Waltham, MA
Y-27632	BDB562822	Fisher	Fair Lawn, NJ
BDNF	450-02	Peprotech	Rocky Hill, NJ
NT3	450-03	Peprotech	Rocky Hill, NJ
N2 Supplement	17502-048	Gibco	Waltham, MA
NEAA Supplement	11140-050	Gibco	Waltham, MA
Neurobasal-A	12349-015	Gibco	Waltham, MA
CultureOne	A33202-01	Gibco	Waltham, MA
BrainPhys	5790	Stemcell Technologies	Cambridge MA
SM1 supplement	5711	Stemcell Technologies	Cambridge MA
TeSR-E8	5990	Stemcell Technologies	Cambridge, MA
doxycycline	D3447	Sigma	St. Louis, MO
Compound E	565790	Millipore	Waltham, MA
**Plasmids**			
pX335 vector	42335	Addgene	Watertown, MA
pUCminusMCS		BlueHeron Biotechnology	Bothell, WA
pUCM-CLYBL-Ngn1-Ngn2-RFP		Michael Ward	NIH
pUCM-CLYBL-hNgn2,Il1,Lhx3-RFP		Michael Ward	NIH
pTLC13-L1		Michael Ward	NIH
pLTC13-R1		Michael Ward	NIH
**siRNA**			
ON-TARGETplus ATG5 Smartpool siRNA	L-004374-00-0005	Dharmacon	LaFayette, CO
ON-TARGETplus MAP1LC3B Smartpool siRNA	D-001810-01-05	Dharmacon	LaFayette, CO
**Plate/Plate reagents**			
TPP 96w plates		Midsci	St. Louis, MO
Polyethyleneimine solution	P3143	Sigma	St. Louis, MO
Boric acid	A73-500	Fisher Chemical	Fair Lawn, NJ
sodium tetraborate	221732	Sigma	St. Louis, MO
ViewPlate 384w plates	6007460	Perkin Elmer	Waltham, MA
Vitronectin	A14700	Gibco	Waltham, MA
8-well hydrophobic polymer μ-slides	80821	Ibidi	Gräfelfing, Germany
**Miscellaneous**			
Lipofectamine 2000	11668019	Invitrogen	Waltham, MA
RIPA buffer	89900	Thermo	Waltham, MA
cOmplete, Mini, EDTA-free Protease Inhibitor cocktail	11836170001	Millipore	Waltham, MA
PhosSTOP phosophatase inhibitor tablets	4906845001	Millipore	Waltham, MA
DharmaFECT 1	T-2001-02	Dharmacon	LaFayette, CO
Lipofectamine Stem	STEM00003	Invitrogen	Waltham, MA
Accutase	A6964	Sigma	St. Louis, MO

Equipment	Manufacturer	Manufacturer location
Multidrop Combi	Thermo Scientific	Waltham, MA
Biomek FX^P laboratory automation workstation	Beckman Coulter	Brea, CA
ImageXpress Micro	Molecular Devices	San Jose, CA
FITC-3540B-NTE-ZERO filter	Semrock	Rochester, NY
TxRed-4040C-NTE-ZERO filter	Semrock	Rochester, NY
Brightline DAPI-5060-NTE-ZERO filter	Semrock	Rochester, NY
BioMek FX pintool	Beckman Coulter	Brea, CA
Lambda XL lamp	Sutter Instruments	Novata, CA
Eclipse Ti inverted microscope	Nikon	Tokyo, Japan
Andor Zyla, 4.2(+) sCMOS camera	Oxford Instruments	Abingdon, UK
ONI Nanoimager	Oxford Nanoimaging	Oxford, UK

Fig. 3 source data

Row Labels	RFP T9/T0	DMSO normalized	Torin1 equivalents	Z-score	status (T9/T0)	Half-Life	status (half-life)	9H RFP unlabelled	intrinsic fluorescence
Torin1	0.1845137	0.250268516	1	16.995801	enhancer	7.6705865	enhancer	#N/A	#N/A
Rottlerin	0.3103447	0.42094171	0.772354239	13.126779	enhancer	9.6050982	enhancer	0.797956473	NO
PI-103	0.323565	0.438873329	0.74843685	12.720284	enhancer	8.0504922	enhancer	0.716262471	NO
DISULFIRAM	0.4847135	0.657450091	0.456896791	7.7653271	enhancer	10.687805	enhancer	0.752091873	NO
GERI-BP002A	0.5231492	0.709583059	0.387361272	6.5835153	enhancer	11.471128	non-significant	0.677914507	NO
Penitrem A	0.5260775	0.713554907	0.382063577	6.4934767	enhancer	10.5485	enhancer	0.718935518	NO
CICLOPIROX OLAMINE	0.5327825	0.722649326	0.369933342	6.2873136	enhancer	9.7168813	enhancer	0.709758632	NO
Forskolin	0.5541604	0.751645695	0.33125767	5.6299896	enhancer	13.252945	non-significant	0.748086874	NO
BIX01294 (hydrochloride hydrate)	0.5576893	0.756432066	0.324873557	5.5214865	enhancer	10.726347	enhancer	1.171951492	YES
NVP-BEZ235	0.5599758	0.759533446	0.320736902	5.4511807	enhancer	10.538364	enhancer	0.697428956	NO
Akt Inhibitor X	0.5710251	0.774520418	0.300747116	5.1114383	enhancer	11.093564	enhancer	0.695030009	NO
Rapamycin	0.5740593	0.778635893	0.295257851	5.0181438	enhancer	11.295024	enhancer	0.701176479	NO
LOPERAMIDE HYDROCHLORIDE	0.5814055	0.788600034	0.281967571	4.7922648	enhancer	10.977134	enhancer	0.708235618	NO
3-AMINOBENZAMIDE	0.5816835	0.78897711	0.281464624	4.7837169	enhancer	11.449877	non-significant	0.760392802	NO
CAPTOPRIL	0.5860618	0.794915658	0.273543723	4.6490948	enhancer	13.49353	non-significant	0.736081605	NO
Salermide	0.596901	0.809617597	0.253934118	4.3158138	enhancer	12.906283	non-significant	0.829920965	NO
Etoposide	0.5982324	0.811423461	0.251525437	4.2748764	enhancer	11.392792	non-significant	0.822720117	NO
Rho Kinase Inhibitor III, Rockout	0.6028512	0.817688328	0.243169289	4.1328569	enhancer	12.460079	non-significant	0.719856483	NO
Tenovin-6	0.6149372	0.834081369	0.221304073	3.7612401	enhancer	10.974576	enhancer	0.739717126	NO
SP 600125	0.6156017	0.83498269	0.220101881	3.7408079	enhancer	13.931769	non-significant	0.648998264	NO
Cumene hydroperoxide	0.6167726	0.836570869	0.217983551	3.7048051	enhancer	11.240963	enhancer	0.749216947	NO
NSC 56817	0.6223491	0.844134667	0.207894874	3.53334	enhancer	11.849385	non-significant	0.741541212	NO
Piceatannol	0.6300319	0.854555326	0.193995686	3.2971122	enhancer	12.5709	non-significant	0.777472288	NO
Apicidin	0.6332537	0.858925335	0.188166922	3.1980476	enhancer	15.05751	non-significant	0.772912482	NO
D609	0.6351433	0.861488237	0.184748495	3.1399487	enhancer	12.741442	non-significant	0.869500013	NO
MCCSL-JR-1-012	0.6408857	0.869277029	0.17435972	2.9633832	non-significant	14.017406	non-significant	#N/A	#N/A
PromethazineA?HCl	0.6427614	0.871821268	0.170966185	2.9057073	non-significant	13.761132	non-significant	#N/A	#N/A
RESVERATROL	0.6434143	0.872706748	0.169785123	2.8856342	non-significant	12.922154	non-significant	#N/A	#N/A
TEMPO	0.6441342	0.873683309	0.168482574	2.8634964	non-significant	13.614936	non-significant	#N/A	#N/A
Tunicamycin	0.6456125	0.875688382	0.165808187	2.818043	non-significant	12.035867	non-significant	#N/A	#N/A
Carvedilol	0.6469153	0.87745541	0.163451305	2.7779859	non-significant	13.237755	non-significant	#N/A	#N/A
HC Toxin	0.6487061	0.879884452	0.160211423	2.7229215	non-significant	13.863155	non-significant	#N/A	#N/A
BAICALEIN	0.6513054	0.883410037	0.155508959	2.6429994	non-significant	12.401902	non-significant	#N/A	#N/A
ABC294640?HCl	0.6533247	0.886148939	0.151855782	2.5809107	non-significant	12.909977	non-significant	#N/A	#N/A
CAY10433	0.6538928	0.88691949	0.150828013	2.563443	non-significant	12.938385	non-significant	#N/A	#N/A
UNC0224	0.6559137	0.889660605	0.147171884	2.5013041	non-significant	14.557699	non-significant	#N/A	#N/A
UNC0638	0.656214	0.890067899	0.14662863	2.4920711	non-significant	12.827412	non-significant	#N/A	#N/A
ESCULETIN	0.6581382	0.892677894	0.14314739	2.4329046	non-significant	14.904711	non-significant	#N/A	#N/A
3230-2939	0.6609233	0.89645552	0.138108752	2.3472689	non-significant	13.121287	non-significant	#N/A	#N/A
6164173	0.6625553	0.89866912	0.135156228	2.2970884	non-significant	12.818855	non-significant	#N/A	#N/A
Ibuproxam	0.6647248	0.901611654	0.131231445	2.2303836	non-significant	12.70063	non-significant	#N/A	#N/A
17-AAG	0.6651939	0.90224801	0.130382666	2.2159579	non-significant	16.444797	non-significant	#N/A	#N/A
PROBUCOL	0.6652105	0.902270495	0.130352675	2.2154482	non-significant	13.569946	non-significant	#N/A	#N/A
NSC 326231	0.6672498	0.905036501	0.126663346	2.1527451	non-significant	12.550828	non-significant	#N/A	#N/A
2-PCPA (hydrochloride)	0.667518	0.905400239	0.12617918	2.1444995	non-significant	13.081273	non-significant	#N/A	#N/A
Garcinol	0.6677036	0.905652009	0.125842376	2.138792	non-significant	12.561633	non-significant	#N/A	#N/A
ROTENONE	0.6678778	0.905888368	0.125527117	2.1334339	non-significant	18.494689	inhibitor	#N/A	#N/A
PD-98059	0.6690633	0.907496353	0.123382369	2.0969823	non-significant	14.28599	non-significant	#N/A	#N/A
Selenomethionine	0.6690923	0.907535581	0.123330047	2.096093	non-significant	12.914913	non-significant	#N/A	#N/A
Rosmarinic acid	0.6692059	0.907689738	0.123124431	2.0925984	non-significant	14.665454	non-significant	#N/A	#N/A
Sorafenib tosylate	0.6696066	0.908233228	0.122399517	2.0802779	non-significant	21.413428	inhibitor	#N/A	#N/A
Imiquimod	0.6709463	0.910050353	0.119975817	2.0390852	non-significant	12.904711	non-significant	#N/A	#N/A
NSC 407286	0.6709971	0.910119266	0.1198839	2.037523	non-significant	13.288539	non-significant	#N/A	#N/A
TAMOXIFEN CITRATE	0.6732675	0.913185079	0.115794685	1.9680235	non-significant	12.253597	non-significant	#N/A	#N/A
C2-dihydroceramide	0.6736409	0.913705257	0.115100866	1.9562315	non-significant	13.330555	non-significant	#N/A	#N/A
PAEONOL	0.6739029	0.914060563	0.114626954	1.948177	non-significant	12.672234	non-significant	#N/A	#N/A
1,2-Dithiole-3-thione	0.6746211	0.915034783	0.113327529	1.9260922	non-significant	13.275427	non-significant	#N/A	#N/A
U83836EA?2HCl	0.6747259	0.915176821	0.113138078	1.9228723	non-significant	13.896601	non-significant	#N/A	#N/A
4112-3315	0.6772235	0.918564556	0.108619481	1.8460751	non-significant	13.294124	non-significant	#N/A	#N/A
FK-866	0.678911	0.92085347	0.105566501	1.7941873	non-significant	13.815413	non-significant	#N/A	#N/A
AMIODARONE HYDROCHLORIDE	0.6795477	0.921716957	0.104414773	1.7746127	non-significant	13.334482	non-significant	#N/A	#N/A
TRIFLUOPERAZINE HYDROCHLORID	0.6824609	0.92566842	0.099144268	1.6850363	non-significant	12.380494	non-significant	#N/A	#N/A
DCHA	0.6829555	0.926339302	0.098249439	1.669828	non-significant	14.983584	non-significant	#N/A	#N/A
7-Ketocholesterol	0.6836032	0.927217812	0.097077674	1.6499129	non-significant	13.527055	non-significant	#N/A	#N/A
Rolipram	0.6838534	0.927557076	0.096625159	1.642222	non-significant	12.541883	non-significant	#N/A	#N/A
TTFA	0.6843544	0.928236686	0.095718688	1.6268158	non-significant	13.861619	non-significant	#N/A	#N/A
Trehalose	0.6846515	0.928639656	0.095181202	1.6176808	non-significant	13.490402	non-significant	#N/A	#N/A
Thiourea	0.6856344	0.929972821	0.093403012	1.587459	non-significant	13.31797	non-significant	#N/A	#N/A
AICAR	0.6856387	0.929978654	0.093395232	1.5873268	non-significant	13.507984	non-significant	#N/A	#N/A
Sodium Butyrate	0.6868209	0.931582134	0.091256493	1.5509772	non-significant	12.965035	non-significant	#N/A	#N/A
Anacardic Acid	0.6870282	0.931863258	0.090881526	1.5446044	non-significant	13.086416	non-significant	#N/A	#N/A
AS-605240	0.6874543	0.93244129	0.090110542	1.5315009	non-significant	12.856711	non-significant	#N/A	#N/A
Z36	0.6880273	0.933218471	0.089073929	1.5138828	non-significant	12.054466	non-significant	#N/A	#N/A
DTT	0.6890869	0.934655626	0.087157036	1.4813037	non-significant	14.250724	non-significant	#N/A	#N/A
GLUTATHIONE	0.6891187	0.93469877	0.08709949	1.4803256	non-significant	13.854817	non-significant	#N/A	#N/A
Phenidone	0.6897575	0.935565309	0.08594369	1.4606819	non-significant	13.428445	non-significant	#N/A	#N/A
Tanshinone IIA	0.6904378	0.936488021	0.084712968	1.4397648	non-significant	17.414632	inhibitor	#N/A	#N/A
Ambroxol	0.6910091	0.937262825	0.083679525	1.4222006	non-significant	13.906999	non-significant	#N/A	#N/A
SU11652	0.6915105	0.937942945	0.082772374	1.4067828	non-significant	48.136378	inhibitor	#N/A	#N/A
Glucosamine HCl	0.6933736	0.940469964	0.079401809	1.3494974	non-significant	13.291728	non-significant	#N/A	#N/A
CAFFEIC ACID	0.6945561	0.942073893	0.07726247	1.3131376	non-significant	13.501902	non-significant	#N/A	#N/A
2',5'-Dideoxyadenosine	0.6945566	0.942074617	0.077261505	1.3131212	non-significant	13.13408	non-significant	#N/A	#N/A
PMSF	0.694896	0.942535005	0.076647435	1.3026846	non-significant	13.47156	non-significant	#N/A	#N/A
BHT	0.69497	0.94263538	0.076513553	1.3004092	non-significant	13.223421	non-significant	#N/A	#N/A
Quinine HCl?2H2O	0.6951295	0.9428517	0.076225024	1.2955054	non-significant	13.027703	non-significant	#N/A	#N/A
EX-527	0.695462	0.943302609	0.075623596	1.2852836	non-significant	14.076725	non-significant	#N/A	#N/A
MS-275	0.6965075	0.944720718	0.073732107	1.2531362	non-significant	13.713392	non-significant	#N/A	#N/A
Deoxycholate?Na	0.6977675	0.946429811	0.0714525	1.2143925	non-significant	13.377787	non-significant	#N/A	#N/A
UNC0321 (trifluoroacetate salt)	0.6981208	0.946908997	0.070813357	1.2035297	non-significant	12.70814	non-significant	#N/A	#N/A
1-Naphthoic Acid	0.6983388	0.947204633	0.070419035	1.1968279	non-significant	13.556277	non-significant	#N/A	#N/A
CPD000466364_IDEBENONE	0.6984884	0.947407546	0.070148386	1.192228	non-significant	15.711231	non-significant	#N/A	#N/A

6-Gingerol	0.6985388	0.947475958	0.070057137	1.1906772	non-significant	13.358118	non-significant	#N/A	#N/A
Sinefungin	0.6993167	0.948531109	0.068649766	1.1667578	non-significant	13.576786	non-significant	#N/A	#N/A
HNHA	0.7000541	0.949531265	0.067315747	1.1440851	non-significant	13.052494	non-significant	#N/A	#N/A
Ascorbic acid	0.700384	0.949978672	0.066718991	1.1339427	non-significant	13.848459	non-significant	#N/A	#N/A
JGB1741	0.7004477	0.950065049	0.06660378	1.1319846	non-significant	12.879091	non-significant	#N/A	#N/A
Protocatechuic acid	0.701093	0.950940384	0.065436249	1.1121415	non-significant	13.703954	non-significant	#N/A	#N/A
NSC 18804	0.7017944	0.95189175	0.064167306	1.0905748	non-significant	12.177925	non-significant	#N/A	#N/A
Diludin	0.7022459	0.952504193	0.063350424	1.0766912	non-significant	14.062481	non-significant	#N/A	#N/A
CPD000449326_4-Thiazolidinecarboxy	0.7036179	0.954365045	0.060868398	1.0345072	non-significant	13.533864	non-significant	#N/A	#N/A
CPD000466276_1H-Imidazol-2-amine,	0.7043738	0.955390382	0.059500793	1.0112637	non-significant	13.200455	non-significant	#N/A	#N/A
D-I?-Tocopherylquinone	0.7046104	0.955711287	0.059072766	1.003989	non-significant	14.383259	non-significant	#N/A	#N/A
MINOXIDIL	0.7047903	0.955955268	0.058747343	0.9984582	non-significant	13.890222	non-significant	#N/A	#N/A
NSC 170984	0.7056037	0.957058535	0.057275793	0.973448	non-significant	13.721542	non-significant	#N/A	#N/A
EHNA?HCl	0.705843	0.957383161	0.056842803	0.966089	non-significant	13.723097	non-significant	#N/A	#N/A
CPD000058212_NICOTINAMIDE	0.7062482	0.957932787	0.056109706	0.9536294	non-significant	13.310156	non-significant	#N/A	#N/A
Canthaxanthin	0.7067149	0.958565759	0.05526544	0.9392805	non-significant	12.625416	non-significant	#N/A	#N/A
L-Ergothioneine	0.7070655	0.959041276	0.054631191	0.9285009	non-significant	13.561904	non-significant	#N/A	#N/A
2-Deoxyglucose	0.7071187	0.959113397	0.054534996	0.926866	non-significant	13.80853	non-significant	#N/A	#N/A
NSC 4972	0.7077556	0.95997732	0.053382685	0.9072815	non-significant	14.692047	non-significant	#N/A	#N/A
Licochalcone A	0.7083841	0.960829732	0.052245729	0.887958	non-significant	15.427902	non-significant	#N/A	#N/A
Bakuchiol	0.7090854	0.961780983	0.05097694	0.8663939	non-significant	14.106679	non-significant	#N/A	#N/A
I-BET	0.7096266	0.962515062	0.049997817	0.849753	non-significant	14.530053	non-significant	#N/A	#N/A
PHENETHYL CAFFEATE (CAPE)	0.7100709	0.963117745	0.049193952	0.8360906	non-significant	13.344736	non-significant	#N/A	#N/A
Dibutyryl cAMP?Na	0.7104859	0.963680545	0.048443283	0.8233324	non-significant	15.123536	non-significant	#N/A	#N/A
Cl-Amidine	0.7121436	0.965929094	0.045444145	0.7723597	non-significant	13.327965	non-significant	#N/A	#N/A
EBSELEN	0.7125793	0.966519982	0.044656012	0.7589647	non-significant	14.191429	non-significant	#N/A	#N/A
RETINYL PALMITATE	0.7130041	0.967096228	0.043887408	0.7459017	non-significant	14.411791	non-significant	#N/A	#N/A
Tetramethylpyrazine	0.7141056	0.968590295	0.041894606	0.7120324	non-significant	13.692422	non-significant	#N/A	#N/A
DMOG	0.7145533	0.969197541	0.041084655	0.6982666	non-significant	13.807448	non-significant	#N/A	#N/A
EUGENOL	0.7145708	0.969221201	0.041053097	0.6977303	non-significant	14.460091	non-significant	#N/A	#N/A
Suramin (sodium salt)	0.7145882	0.969244774	0.041021655	0.6971959	non-significant	14.49206	non-significant	#N/A	#N/A
Pimelic Diphenylamide 106	0.7147738	0.969496613	0.040685749	0.6914869	non-significant	12.925041	non-significant	#N/A	#N/A
GENTISIC ACID	0.7149077	0.969678204	0.040443542	0.6873704	non-significant	12.976569	non-significant	#N/A	#N/A
CAPSAICIN	0.7151177	0.969963046	0.040063616	0.6809133	non-significant	15.802715	non-significant	#N/A	#N/A
F-Amidine (trifluoroacetate salt)	0.7170142	0.972535325	0.036632682	0.6226018	non-significant	14.358549	non-significant	#N/A	#N/A
MELATONIN	0.7189995	0.975228202	0.033040893	0.5615565	non-significant	13.296126	non-significant	#N/A	#N/A
Tenovin-1	0.7201312	0.976763166	0.030993542	0.5267601	non-significant	14.187049	non-significant	#N/A	#N/A
S-Adenosylhomocysteine	0.7208613	0.97775351	0.02967261	0.5043098	non-significant	13.492445	non-significant	#N/A	#N/A
D-I?-Tocopherol	0.7209514	0.977875719	0.029509606	0.5015394	non-significant	14.238019	non-significant	#N/A	#N/A
Anethole trithione	0.7213154	0.978369444	0.02885107	0.4903471	non-significant	13.27882	non-significant	#N/A	#N/A
Apigenin	0.7219271	0.979199125	0.027744434	0.4715389	non-significant	15.994701	non-significant	#N/A	#N/A
CPD000466338_TEMOZOLOMIDE	0.7225618	0.980059946	0.026596261	0.4520248	non-significant	13.740581	non-significant	#N/A	#N/A
Tocopherol succinate	0.7235018	0.981334968	0.024895623	0.4231211	non-significant	15.110349	non-significant	#N/A	#N/A
(?)-Neplanocin A	0.7236929	0.981594114	0.024549971	0.4172464	non-significant	15.092096	non-significant	#N/A	#N/A
8-CPT-cAMP?Na	0.7259161	0.984609572	0.02052792	0.3488885	non-significant	13.717633	non-significant	#N/A	#N/A
CARBAMAZEPINE	0.7266267	0.985573416	0.019242334	0.3270389	non-significant	15.432225	non-significant	#N/A	#N/A
CAY10603	0.7281017	0.987574069	0.016573841	0.2816857	non-significant	20.718845	inhibitor	#N/A	#N/A
NITRENDIPINE	0.7283571	0.987920558	0.016111691	0.2738311	non-significant	13.701487	non-significant	#N/A	#N/A
Niguldipine	0.7285959	0.988244441	0.015679693	0.2664889	non-significant	14.74542	non-significant	#N/A	#N/A
2',3',5'-triacetyl-5-Azacytidine	0.7294329	0.989379719	0.014165446	0.2407531	non-significant	13.275429	non-significant	#N/A	#N/A
TOLAZAMIDE	0.7300159	0.990170538	0.013110643	0.2228259	non-significant	13.586633	non-significant	#N/A	#N/A
Splitomicin	0.7317913	0.992578516	0.009898855	0.168239	non-significant	13.982223	non-significant	#N/A	#N/A
Rilmenidine	0.7329386	0.994134687	0.007823218	0.1329619	non-significant	14.145357	non-significant	#N/A	#N/A
ISOLIQUIRITIGENIN	0.7332201	0.994516591	0.00731383	0.1243044	non-significant	13.689336	non-significant	#N/A	#N/A
Ferulic acid ethylester	0.7337784	0.995273748	0.006303926	0.1071403	non-significant	14.160967	non-significant	#N/A	#N/A
NDGA	0.735594	0.997736439	0.003019162	0.0513131	non-significant	13.295177	non-significant	#N/A	#N/A
3-METHYL-1-PHENYL-2-PYRAZOLIN-	0.7362283	0.998596717	0.001871714	0.0318113	non-significant	13.110185	non-significant	#N/A	#N/A
SB-202190	0.7365781	0.999071204	0.001238839	0.0210551	non-significant	15.052851	non-significant	#N/A	#N/A
DMSO	0.7372628	1	0	NA	NA	14.11855	non-significant	0.763576208	NO
L-690,330	0.737532	1.000365131	-0.000487015	-0.008277	non-significant	16.199327	non-significant	#N/A	#N/A
Dacinostat	0.7384254	1.001576868	-0.002103244	-0.035746	non-significant	17.197464	inhibitor	#N/A	#N/A
Zebularine	0.7389284	1.002259108	-0.003013223	-0.051212	non-significant	14.284189	non-significant	#N/A	#N/A
CPD000469175_IMATINIB MESYLATE	0.7389481	1.002285817	-0.003048847	-0.051818	non-significant	17.394754	inhibitor	#N/A	#N/A
NSC 62794	0.7401878	1.003967255	-0.005291567	-0.089934	non-significant	14.604843	non-significant	#N/A	#N/A
Trolox	0.7401939	1.003975567	-0.005302655	-0.090123	non-significant	14.079224	non-significant	#N/A	#N/A
EPIGALLOCATECHIN-3-MONOGALLA	0.7419143	1.006309032	-0.008415056	-0.143021	non-significant	15.198126	non-significant	#N/A	#N/A
VERAPAMIL HYDROCHLORIDE	0.7421686	1.006654052	-0.008875247	-0.150842	non-significant	14.749147	non-significant	#N/A	#N/A
TEMPOL	0.742277	1.006801056	-0.009071323	-0.154174	non-significant	13.785905	non-significant	#N/A	#N/A
Suramin?6Na	0.7441885	1.009393755	-0.012529492	-0.212949	non-significant	15.017659	non-significant	#N/A	#N/A
HBEDA?HClA?H2O	0.7445128	1.009833578	-0.013116133	-0.222919	non-significant	13.811366	non-significant	#N/A	#N/A
PX105684	0.7457386	1.011496244	-0.015333815	-0.26061	non-significant	17.130965	inhibitor	#N/A	#N/A
2,4-DPD	0.7474539	1.013822872	-0.018437097	-0.313353	non-significant	13.670234	non-significant	#N/A	#N/A
VALPROATE SODIUM	0.7477982	1.014289829	-0.019059929	-0.323939	non-significant	13.161775	non-significant	#N/A	#N/A
5147472	0.7479042	1.014433594	-0.019251685	-0.327198	non-significant	14.755466	non-significant	#N/A	#N/A
Metformin?HCl	0.7481351	1.014746845	-0.019669502	-0.334299	non-significant	12.672044	non-significant	#N/A	#N/A
THIOCTIC ACID	0.7490502	1.015987973	-0.021324932	-0.362434	non-significant	15.066169	non-significant	#N/A	#N/A
Chidamide	0.7493478	1.016391668	-0.021863385	-0.371586	non-significant	16.529833	non-significant	#N/A	#N/A
CDC	0.7535984	1.022157015	-0.029553267	-0.502281	non-significant	14.959331	non-significant	#N/A	#N/A
3-Deazaneplanocin A	0.7538187	1.022455865	-0.029951876	-0.509056	non-significant	15.300576	non-significant	#N/A	#N/A
Scriptaid	0.7559996	1.025413962	-0.033897419	-0.576114	non-significant	20.230442	inhibitor	#N/A	#N/A
GENISTEIN	0.7570347	1.026817921	-0.035770034	-0.60794	non-significant	13.756717	non-significant	#N/A	#N/A
Seratrodast	0.7585029	1.028809278	-0.038426129	-0.653083	non-significant	12.779962	non-significant	#N/A	#N/A
4-iodo-SAHA	0.7600864	1.030957099	-0.041290916	-0.701772	non-significant	16.240431	non-significant	#N/A	#N/A
NSC 401077	0.760324	1.031279361	-0.041720752	-0.709078	non-significant	13.008035	non-significant	#N/A	#N/A
NVP-LBH589	0.761611	1.033025026	-0.044049139	-0.74865	non-significant	18.713396	inhibitor	#N/A	#N/A
CAFFEINE	0.7650943	1.037749664	-0.050350912	-0.855754	non-significant	14.199144	non-significant	#N/A	#N/A
Trichostatin A	0.7661821	1.039225222	-0.052319027	-0.889204	non-significant	18.536443	inhibitor	#N/A	#N/A
HYDROQUINONE	0.7669227	1.040229743	-0.053658868	-0.911975	non-significant	13.060518	non-significant	#N/A	#N/A
NIMODIPINE	0.7682313	1.042004648	-0.056026256	-0.952211	non-significant	14.066964	non-significant	#N/A	#N/A
LY 294002	0.7755873	1.051982032	-0.069334199	-1.17839	non-significant	13.702093	non-significant	#N/A	#N/A
Hydroxychloroquine	0.777176	1.054136897	-0.072208381	-1.227239	non-significant	14.045034	non-significant	#N/A	#N/A
ETYA	0.7796496	1.057492019	-0.076683479	-1.303297	non-significant	15.026039	non-significant	#N/A	#N/A
CBHA	0.7927508	1.075262178	-0.100385511	-1.706132	non-significant	20.865427	inhibitor	#N/A	#N/A
ETHOXYQUIN	0.7966318	1.080526141	-0.107406642	-1.825462	non-significant	14.50667	non-significant	#N/A	#N/A
NSC 26326	0.8029713	1.089124925	-0.118875794	-2.020389	non-significant	15.110592	non-significant	#N/A	#N/A

SAHA	0.8036859	1.090094133	-0.120168534	-2.042361	non-significant	20.38805	inhibitor	#N/A	#N/A
n-Octyl caffeate	0.8057866	1.092943473	-0.123969014	-2.106953	non-significant	16.966887	inhibitor	#N/A	#N/A
AGK2	0.8134501	1.103338048	-0.137833412	-2.342589	non-significant	16.32571	non-significant	#N/A	#N/A
CYCLOHEXIMIDE	0.8429947	1.143411287	-0.191283533	-3.251017	inhibitor	18.869563	inhibitor	0.826837469	NO
U74389G maleate	0.8635296	1.171264187	-0.228434033	-3.882419	inhibitor	16.401557	non-significant	0.926349295	YES
Tubastatin A (trifluoroacetate salt)	0.8732561	1.184456911	-0.246030633	-4.181488	inhibitor	20.39129	inhibitor	0.798336888	NO
ALLN	0.8844253	1.199606531	-0.26623736	-4.524917	inhibitor	28.37093	inhibitor	0.857586629	NO
MG132	0.9196539	1.247389418	-0.329970694	-5.608116	inhibitor	49.557645	inhibitor	0.789265	NO
Curcumin	0.9772698	1.32553788	-0.434205961	-7.379678	inhibitor	16.663749	non-significant	2.697478274	YES
NICLOSAMIDE	1.1013045	1.493774533	-0.658601837	-11.19347	inhibitor	-49.30223	inhibitor	0.675069842	NO
Bafilomycin A1	1.2150858	1.648103969	-0.864448116	-14.69199	inhibitor	106.21429	inhibitor	0.865698976	NO
CPD000469152_TERBINAFINE HCl	1.4206071	1.926866562	-1.236264691	-21.01131	inhibitor	-20.11502	inhibitor	0.759322914	NO
GF 109203X	3.4106494	4.62609697	-4.836527537	-82.20066	inhibitor	-40.01382	inhibitor	10.40788771	YES

Figure 5-source data

Compound	Primary GFP	Primary Group	Primary Z-score	RFP_9H	Secondary Z_score	Standard Error	Secondary Group	plate	rank	Label	RFP 9H unlabelled	intrinsic fluorescence
Torin1	#N/A	enhancer puncta	#N/A	0.16264	12.77375092	0.004073812	enhancer	plate 1	1	Torin1	#N/A	#N/A
Digoxigenin	0.25636422	enhancer	3.495227252	0.47716	10.46955572	0.010182837	enhancer	plate 2	2	Digoxigenin	#N/A	NO
CICLOPIROX OLAMINE	0.41788536	non-significant	2.96994629	0.53314	4.386733894	0.008868595	enhancer	plate 1	3	Ciclopirox Olamine	0.826837469	NO
Lanatoside C	0.41026219	enhancer	5.593446654	0.53402	7.475024566	0.006147924	enhancer	plate 2	4		0.62992903	NO
MITOXANTHRONE HYDROCHLORIDE	0.84579253	enhancer	6.011118446	0.54139	7.08712015	0.014294841	enhancer	plate 2	5		0.709758632	YES
Clofilium tosylate	0.62546862	enhancer	6.509612658	0.57243	3.497538865	0.028649703	enhancer	plate 1	6	Clofilium tosylate	#N/A	NO
Proscillaridin A	0.44034228	enhancer	7.245694819	0.57332	5.405245334	0.002805423	enhancer	plate 2	7		0.724563422	NO
ASTEMIZOLE	0.36700274	enhancer	3.81960912	0.57944	3.338753735	0.017360156	enhancer	plate 1	8	Astemizole	#N/A	NO
CLIOQUINOL	0.32256505	enhancer	4.397798441	0.5915	3.065859359	0.028559644	enhancer	plate 1	9		#N/A	NO
Irinotecan hydrochloride trihydrate	0.4780429	enhancer puncta	6.51755767	0.59903	2.89538224	0 n=1 due to arti	non-significant	plate 1	11		#N/A	#N/A
CAMPTOTHECIN	0.6053243	enhancer	6.299959084	0.61347	2.568519237	0.013899469	non-significant	plate 1	12		#N/A	#N/A
Benidipine hydrochloride	0.48895442	enhancer	8.045592404	0.61504	3.208069089	0.036972787	enhancer	plate 2	13		5.691125987	NO
PERHEXILINE MALEATE	0.47856857	enhancer	4.980739055	0.61811	2.463469972	0.03632359	non-significant	plate 1	14		#N/A	#N/A
Thiethylperazine dimalate	0.48550856	enhancer puncta	7.988891758	0.61837	3.033011481	0.003838942	enhancer	plate 2	15		0.703863488	NO
Hemicholinium bromide	-0.4531873	inhibitor	-3.220840366	0.62241	2.366101252	0.034648778	non-significant	plate 1	16		#N/A	#N/A
Pinaverium bromide	0.42260348	enhancer	5.761705784	0.62308	2.784758671	0.007543822	non-significant	plate 2	17		0.739777393	#N/A
DEQUALINIUM CHLORIDE	0.41039736	non-significant	2.916728428	0.62308	2.350888112	0.020482559	non-significant	plate 1	18		#N/A	#N/A
METHYLBENZETHONIUM CHLORIDE	0.91268008	enhancer	12.44332903	0.62409	2.731418006	0.009049988	non-significant	plate 2	19		0.783967327	#N/A
PAROXETINE HYDROCHLORIDE	0.34198959	enhancer	4.662629355	0.631	2.171653741	0.008318707	non-significant	plate 1	20		#N/A	#N/A
FLUPHENAZINE HYDROCHLORIDE	-0.2896035	inhibitor	-3.014070173	0.63136	2.163477921	0.007016677	non-significant	plate 1	21		#N/A	#N/A
MONOBENZONE	-0.2440303	inhibitor	-3.327068637	0.63386	2.106808577	0.005542692	non-significant	plate 1	22		#N/A	#N/A
Chlorhexidine	0.75528286	enhancer	7.860664256	0.63522	2.076073695	0.017631397	non-significant	plate 1	24		#N/A	#N/A
Mometasone furoate	0.49105052	enhancer puncta	3.489937207	0.63897	1.991259899	0.012581924	non-significant	plate 1	26		#N/A	#N/A
MEFLOQUINE	0.3319713	enhancer	3.455016753	0.6419	1.924889799	0.012698719	non-significant	plate 1	27		#N/A	#N/A
Melengestrol acetate	0.20525549	enhancer	3.377414955	0.64303	1.899280564	0.018564834	non-significant	plate 1	28		#N/A	#N/A
CPD000059165_BESTATIN	0.23670364	enhancer	3.894884553	0.64317	1.896118184	0.014925094	non-significant	plate 1	29		#N/A	#N/A
CLEMASTINE	0.75579044	enhancer	7.865946968	0.6433	1.893174968	0.006520221	non-significant	plate 1	30		#N/A	#N/A
S(-)Eticlopride hydrochloride	0.24723435	enhancer	3.37075221	0.64352	1.888199675	0 n=1 due to arti	non-significant	plate 1	31		#N/A	#N/A
DIRITHROMYCIN	-0.5164544	inhibitor	-3.670484376	0.64372	1.883691041	0.005478418	non-significant	plate 1	32		#N/A	#N/A
THIORIDAZINE HYDROCHLORIDE	0.92542359	enhancer	9.631416999	0.64833	1.779263613	0.00935512	non-significant	plate 1	33		#N/A	#N/A
Gefitinib	0.31400324	enhancer	4.281068174	0.64863	1.772553124	0.017491893	non-significant	plate 1	34		#N/A	#N/A
CPD000469186_NELFINAVIR MESYLA	0.60961928	enhancer	10.03109499	0.64872	1.434189959	0.005796963	non-significant	plate 2	35		0.845214443	#N/A
PromethazineA?HCl	0.33508446	enhancer	4.568486038	0.65235	1.688334175	0.008751662	non-significant	plate 1	36		#N/A	#N/A
Penbutolol sulfate	0.28373084	enhancer	4.668702478	0.6529	1.675885365	0.023065261	non-significant	plate 1	37		#N/A	#N/A
Ethynodiol diacetate	0.27073023	enhancer	3.691091086	0.65298	1.674142663	0.012057412	non-significant	plate 1	38		#N/A	#N/A
Cyclosporin A	0.53868835	enhancer	3.82850326	0.65708	0.994190768	0.002990588	non-significant	plate 2	39		#N/A	#N/A
GBR 12909 dihydrochloride	0.46558538	enhancer	3.308954362	0.65874	0.906674981	0.00830192	non-significant	plate 2	40		#N/A	#N/A
IDAZOXAN HYDROCHLORIDE	0.43701528	enhancer	5.958193892	0.65879	0.904110495	0.006644269	non-significant	plate 2	41		0.703511717	#N/A
AMINACRINE	0.58954185	enhancer puncta	9.700727096	0.65934	0.874869912	0.00767841	non-significant	plate 2	42		#N/A	#N/A
Halofantrine hydrochloride	0.23789425	enhancer puncta	3.914475583	0.65976	1.520618611	0.010880006	non-significant	plate 1	43		#N/A	#N/A
TENOXICAM	-0.4628417	inhibitor	-3.289454428	0.66007	0.83671378	0.007413766	non-significant	plate 2	44		#N/A	#N/A
FLUNARIZINE HYDROCHLORIDE	0.64299025	enhancer	6.691970378	0.6622	1.465464387	0.024622534	non-significant	plate 1	45		#N/A	#N/A
TOBRAMYCIN	-0.5303392	inhibitor	-3.769165056	0.66424	1.419107467	0 n=1 due to arti	non-significant	plate 1	46		#N/A	#N/A
Tetramisole hydrochloride	-0.5162222	inhibitor	-3.668834442	0.66447	1.414032045	0.008947285	non-significant	plate 1	47		#N/A	#N/A
BIFONAZOLE	0.21800455	enhancer	3.587196739	0.66468	1.409117127	0.025988512	non-significant	plate 1	48		#N/A	#N/A
FLUVOXAMINE MALEATE	0.32104921	enhancer	5.282764564	0.66519	0.566827818	0.005992063	non-significant	plate 2	49		0.793409498	#N/A
(R)-Duloxetine hydrochloride	0.57712202	enhancer puncta	9.496362611	0.66606	0.521208207	0.008358478	non-significant	plate 2	50		#N/A	#N/A
CHLOROQUINE DIPHOSPHATE	-0.5833479	inhibitor	-4.145902448	0.6677	1.340827563	0.00579674	non-significant	plate 1	51		#N/A	#N/A
CPD000469290_SAQUINAVIR MESYLA	0.21267557	enhancer	3.499510193	0.668	1.334098643	0.026679059	non-significant	plate 1	52		#N/A	#N/A
DONEPEZIL HYDROCHLORIDE	0.22495944	enhancer	3.701637312	0.66801	1.333920386	0.017939594	non-significant	plate 1	53		#N/A	#N/A
NSC 257473	0.44333951	enhancer	4.614089982	0.6688	1.316006399	0.008627397	non-significant	plate 1	54		#N/A	#N/A
Azacytidine-5	0.79363653	enhancer	10.82030898	0.67014	0.306090694	0.004012635	non-significant	plate 2	55		#N/A	#N/A
PROADIFEN HYDROCHLORIDE	0.26017043	enhancer	3.547120518	0.67015	1.285456158	0.025991715	non-significant	plate 1	56		#N/A	#N/A
Dipivefrin hydrochloride	-0.4963286	inhibitor	-3.527448987	0.67104	1.265283638	0.020205056	non-significant	plate 1	57		#N/A	#N/A
PERPHENAZINE	0.61889574	enhancer	6.441204919	0.67111	1.263577994	0.014966306	non-significant	plate 1	58		0.763576208	#N/A
Bisoprolol fumarate	-0.4232085	inhibitor	-3.007778244	0.67297	1.221470404	0.016814371	non-significant	plate 1	59		#N/A	#N/A
Quipazine dimaleate salt	-0.4779633	inhibitor	-3.396925118	0.673	1.22091623	0.018289153	non-significant	plate 1	60		#N/A	#N/A
CPD000466343_LETROZOL	0.5367188	enhancer puncta	8.831540259	0.67301	0.155220623	0.006300384	non-significant	plate 2	61		#N/A	#N/A
ASPIRIN	0.21685811	enhancer	3.56833246	0.67357	1.207939524	0.007620018	non-significant	plate 1	62		#N/A	#N/A
CPD000466368_GLIMEPIRIDE	0.41346808	enhancer	5.63715525	0.67404	1.19743859	0.025925192	non-significant	plate 1	63		#N/A	#N/A
CPD000466395_RITONAVIR	0.24017315	enhancer	3.951974192	0.6742	1.193731544	0.012312513	non-significant	plate 1	64		#N/A	#N/A
Carvedilol	0.26235371	enhancer	4.316948325	0.67424	1.192778327	0.02024943	non-significant	plate 1	65		#N/A	#N/A
CHLOROPYRAMINE HYDROCHLORIDE	0.42076681	enhancer puncta	5.736664948	0.67506	1.174276758	0.028189188	non-significant	plate 1	66		#N/A	#N/A
Tegaserod maleate	0.87268382	enhancer	14.35973987	0.67541	0.028516983	0.00809559	non-significant	plate 2	67		#N/A	#N/A
DMSO	#N/A	non-significant	#N/A	0.67549	0.024568329	0.002384526	non-significant	plate 2	68		1.179184516	NO
Hesperidine	-0.5090356	inhibitor	-3.617758548	0.67568	1.160131989	0.01214672	non-significant	plate 1	69		#N/A	#N/A
MITOTANE	0.26411353	enhancer	4.34590566	0.6759	1.155127465	0.012310531	non-significant	plate 1	70		#N/A	#N/A
Mirtazapine	-0.463698	inhibitor	-3.295540233	0.67657	-0.032160421	0.007560238	non-significant	plate 2	71		#N/A	#N/A
RACECADOTRIL	-0.4590654	inhibitor	-3.262616133	0.67675	-0.041885034	0.010522678	non-significant	plate 2	72		#N/A	#N/A
Amikacin hydrate	-0.4277658	inhibitor	-3.04016759	0.6768	-0.044592514	0.006383934	non-significant	plate 2	73		#N/A	#N/A
Kanamycin A sulfate	-0.4872137	inhibitor	-3.462668889	0.6775	-0.081286417	0.011569435	non-significant	plate 2	74		#N/A	#N/A
CPD000466360_FLUBENDAZOLE	0.63493224	enhancer	8.6565609	0.6785	-0.134107445	0.007243105	non-significant	plate 2	75		#N/A	#N/A
NSC 170984	0.44019962	enhancer puncta	4.916136748	0.6795	1.073749974	0.013548053	non-significant	plate 1	76		#N/A	#N/A
Topotecan	0.31810767	enhancer	5.234362465	0.67983	-0.204124039	0.005738607	non-significant	plate 2	77		#N/A	#N/A
Dexfenfluramine hydrochloride	-0.5762799	inhibitor puncta	-4.095669882	0.68013	1.059391628	0.01818349	non-significant	plate 1	78		0.774482364	#N/A
Terazosin hydrochloride	-0.295138	inhibitor	-4.023862949	0.68109	-0.270595863	0.003097366	non-significant	plate 2	79		#N/A	#N/A
GLICLAZIDE	-0.4935736	inhibitor	-3.507868763	0.68122	-0.277264189	0.004194259	non-significant	plate 2	80		#N/A	#N/A
Anethole trithione	0.24132769	enhancer	3.970971859	0.68136	-0.284348293	0.00563113	non-significant	plate 2	81		#N/A	#N/A
ACAMPROSATE CALCIUM	-0.4614512	inhibitor	-3.279572315	0.68141	1.030555459	0.015448197	non-significant	plate 1	82		#N/A	#N/A
ETHOPROPAZINE HYDROCHLORIDE	0.3289878	enhancer	4.485365192	0.68222	-0.329856087	0.00872658	non-significant	plate 2	83		#N/A	#N/A
CPD000466303_LEVETIRACETAM	-0.476691	inhibitor	-3.387883008	0.68294	0.995921752	0.01276144	non-significant	plate 1	84		#N/A	#N/A
PIRACETAM	-0.4740587	inhibitor	-3.3691748	0.68303	-0.37254526	0.006429157	non-significant	plate 2	85		#N/A	#N/A
Ribostamycin sulfate salt	0.27566664	enhancer puncta	3.758393291	0.68415	0.968470696	0.003258565	non-significant	plate 1	86		#N/A	#N/A
BEZAFIBRATE	-0.9902702	inhibitor	-7.03793361	0.68429	-0.438727938	0.006144875	non-significant	plate 2	87		#N/A	#N/A
Sertaconazole nitrate	0.4769561	enhancer	7.848163822	0.68667	-0.564111372	0.008085587	non-significant	plate 2	89		#N/A	#N/A
Algestone acetophenide	0.55067742	enhancer	9.061224991	0.68759	-0.612455973	0.006903801	non-significant	plate 2	90		#N/A	#N/A
CPD000059060_IPRIFLAVONE	0.2104179	enhancer	3.462360778	0.68774	0.887192455	0.01900195	non-significant	plate 1	91		#N/A	#N/A
NSC 697855	0.5255255	enhancer	8.647358141	0.68794	-0.631052253	0.046594802	non-significant	plate 2	92		#N/A	#N/A
FEXOFENADINE HYDROCHLORIDE	0.22522741	enhancer	3.706046723	0.6883	-0.649971618	0.008461063	non-significant	plate 2	93		#N/A	#N/A
Aprepitant	0.37969548	enhancer	5.176705291	0.69092	0.815242056	0.017123455	non-significant	plate 1	94		#N/A	#N/A
BENAZEPRIL HYDROCHLORIDE	-0.4381748	inhibitor	-3.114144737	0.69165	-0.826719111	0.007876651	non-significant	plate 2	95		#N/A	#N/A
SPIPERONE	-0.3090575	inhibitor	-3.216540105	0.69301	0.767893721	0.011783159	non-significant	plate 1	96		#N/A	#N/A
Oxantel pamoate	-0.4519617	inhibitor	-3.212129698	0.69453	-0.978171916	0.010205471	non-significant	plate 2	97		#N/A	#N/A
Tosufloxacin hydrochloride	-0.5281488	inhibitor	-3.753597514	0.69575	0.705990376	0.00939747	non-significant	plate 1	98		#N/A	#N/A
CPD000058319_ETHYNYLESTRADIOL	0.26276319	enhancer	4.323686214	0.69641	-1.077417015	0.000840547	non-significant	plate 2	99		#N/A	#N/A
CPD000112594_Prostaglandin E1	-0.3664235	inhibitor	-6.029384142	0.69653	-1.083550829	0.004553861	non-significant	plate 2	100		#N/A	#N/A
CARBAMAZEPINE	-0.2954291	inhibitor	-3.29934328	0.69679	0.682261081	0.028044619	non-significant	plate 1	101		#N/A	#N/A
CPD000466276_1H-Imidazol-2-amine	-0.2788275	inhibitor	-3.113937769	0.69898	0.632892923	0.009852429	non-significant	plate 1	102		0.763576208	#N/A

LOVASTATIN	-0.5262054	inhibitor	-3.739785549	0.69939	-1.234096598	0.009119661	non-significant	plate 2	103		#N/A	#N/A
Dinoprost trometamol	-0.4602376	inhibitor	-3.270947516	0.6996	0.618663797	0.008638988	non-significant	plate 1	104		#N/A	#N/A
Atorvastatin	-0.2042638	inhibitor	-3.361097864	0.70119	-1.329020137	0.007061163	non-significant	plate 2	105		#N/A	#N/A
SULCONAZOLE NITRATE	0.5005188	enhancer	6.823990351	0.7015	-1.345210792	0.005644672	non-significant	plate 2	106		#N/A	#N/A
SULFAMETER	0.40672644	enhancer	5.54524086	0.70257	0.551422953	0.008216233	non-significant	plate 1	107		#N/A	#N/A
DO 897/99	-0.4865664	inhibitor	-3.458068531	0.7039	-1.471513704	0.006413292	non-significant	plate 2	108		#N/A	#N/A
OXICONAZOLE NITRATE	0.51386189	enhancer	7.00590784	0.70466	-1.511580608	0.008578999	non-significant	plate 2	109		#N/A	#N/A
Etoricoxib	-0.5956954	inhibitor	-4.233657404	0.70469	0.503599328	0.020796188	non-significant	plate 1	110		0.726130427	#N/A
PROPARACAINE HYDROCHLORIDE	0.46583228	enhancer	6.351080169	0.70655	0.461385483	0.010145724	non-significant	plate 1	111		#N/A	#N/A
Estramustine	0.45171892	enhancer	7.432893826	0.70711	-1.6407886	0.009831745	non-significant	plate 2	112		#N/A	#N/A
NAFTIFINE HYDROCHLORIDE	0.21016172	enhancer	3.458145536	0.70755	0.438886633	0.043722179	non-significant	plate 1	113		#N/A	#N/A
Gestrinone	-0.2598281	inhibitor	-4.275390281	0.70759	-1.665919394	0.01395934	non-significant	plate 2	114		#N/A	#N/A
CPD000336944_mevastatin	-0.4510806	inhibitor	-3.205867895	0.71014	-1.800045968	0.004180073	non-significant	plate 2	115		#N/A	#N/A
Tyloxapol	0.42839157	enhancer	5.840619681	0.71024	-1.805697914	0.007311768	non-significant	plate 2	116		#N/A	#N/A
HEXACHLOROPHENE	0.67843993	enhancer	11.16351732	0.71162	-1.878132333	0.009325885	non-significant	plate 2	118		#N/A	#N/A
Amphotericin B	-0.5038243	inhibitor	-3.580721237	0.71165	0.345873361	0.012610234	non-significant	plate 1	119		0.706443472	#N/A
MEBENDAZOLE	0.37593186	enhancer	3.912539619	0.71488	-2.049803369	0.004454286	non-significant	plate 2	120		#N/A	#N/A
DILOXANIDE FUROATE	0.23836408	enhancer	3.249816349	0.71754	0.212573974	0.022902366	non-significant	plate 1	121		#N/A	#N/A
CHLORTETRACYCLINE HYDROCHLORII	0.46579662	enhancer	4.847814096	0.71768	0.209452285	0.019309703	non-significant	plate 1	122		#N/A	#N/A
PRAZOSIN HYDROCHLORIDE	-0.2837255	inhibitor	-3.868266743	0.71855	-2.243398148	0.008318949	non-significant	plate 2	123		#N/A	#N/A
TRICLOSAN	0.57623554	enhancer	9.481775926	0.7209	-2.366996431	0.01784983	non-significant	plate 2	124		#N/A	#N/A
AMOROLFINE HYDROCHLORIDE	0.45026197	enhancer	7.408920167	0.72105	-2.374978195	0.026561846	non-significant	plate 2	125		#N/A	#N/A
Bromocryptine mesylate	0.41298894	enhancer	4.298214103	0.72336	0.080958069	0.024958997	non-significant	plate 1	126		#N/A	#N/A
Avermectin B1a	0.36715902	enhancer	5.00578527	0.72552	-2.610317469	0.01880213	non-significant	plate 2	127		#N/A	#N/A
DMSO	#N/A	non-significant	#N/A	0.72569	0.028082996	0.004960111	non-significant	plate 1	128		0.675069842	NO
DILAZEP DIHYDROCHLORIDE	-0.5391178	inhibitor	-5.610909504	0.73351	-0.14892721	0.01791535	non-significant	plate 1	129		#N/A	#N/A
Ivermectin	0.31826256	enhancer	3.31234195	0.7349	-3.104485911	0.008257464	inhibitor	plate 2	130		#N/A	NO
Haloprogin	0.55120193	enhancer	3.917438312	0.73786	-3.260059023	0.01481143	inhibitor	plate 2	131		#N/A	NO
Estropipate	0.29819842	enhancer	4.06558783	0.73851	-0.262091035	0.020159141	non-significant	plate 1	132		#N/A	#N/A
Alfacalcidol	0.44851495	enhancer	3.187633392	0.75281	-4.047774907	0.009385267	inhibitor	plate 2	133		#N/A	NO
Cilnidipine	0.28226936	enhancer	4.644654177	0.7576	-4.299936095	0.011337818	inhibitor	plate 2	134		#N/A	NO
CYCLOHEXIMIDE	0.63325095	enhancer	8.633638512	0.84598	-2.694714613	0.028425129	non-significant	plate 1	135	Cyclohexamide	#N/A	NO
Quinacrine dihydrochloride hydrate	-2.1091858	inhibitor puncta	-21.95151267	0.85718	-2.948365052	0 n=1 due to arti	non-significant	plate 1	136	Quinacrine	0.723005469	#N/A
DIACERIN	0.29801344	enhancer	4.063065895	0.92739	-4.537680299	0.074528679	inhibitor	plate 1		Diacerin	#N/A	YES
Niclosamide	0.27756754	enhancer	3.099866314	1.10624	-22.66078712	0.026593144	inhibitor	plate 2	137	Niclosamide	#N/A	NO

Fig. 5-source data 2

Row Labels	targets	mode	clinical tria	clinical tria	phase	RFP intens	is_enhance	is_inhibito	z_score_er	z_score_in	Plate	label	STDEV	compound	sterr
PF-04691502	mTOR/PI3K	ATP compe	Breast canc	https://clinic	2	0.013832	1	0	1	0	plate 1	enhancer	0	1	0
GDC-0349	PIK3CA		non-Hodgki	https://clinic	1	0.0636265	1	0	1	0	plate 1	enhancer	0.0363878	2	0.0148553
INK 128 (MLN0128)	mTOR/PI3K	ATP compe	Metastatic (	https://clinic	2	0.0684577	1	0	1	0	plate 1	enhancer	0.0139815	3	0.0057079
BAY 1895344	ATR		Advanced S	https://clinic	1	0.0686379	1	0	1	0	plate 1	enhancer	0.1676537	4	0.0684443
AZD8055	mTOR	ATP compe	Advanced T	https://clinic	1	0.0891148	1	0	1	0	plate 1	enhancer	0.0073983	5	0.0030203
Torkinib (PP242)	mTOR	ATP competitive			preclinical	0.0896238	1	0	1	0	plate 1	enhancer	0.0168801	6	0.007549
WYE-125132	mTOR	ATP competitive			preclinical	0.0940268	1	0	1	0	plate 1	enhancer	0.0114862	7	0.0046892
Vistusertib (AZD2014)	mTOR/PI3K	ATP compe	Metastatic F	https://clinicaltrials.gov/c		0.0968389	1	0	1	0	plate 1	enhancer	0.0143359	8	0.0058526
OSI-027	mTOR	ATP compe	Solid tumors	https://clinic	1	0.0974829	1	0	1	0	plate 1	enhancer	0.0178593	9	0.0079869
CZ415	mTOR	ATP competitive			preclinical	0.0984994	1	0	1	0	plate 1	enhancer	0.0246067	10	0.0100456
Torin1	mTOR	ATP competitive			preclinical	0.1027453	1	0	1	0	plate 1	Plate 1 Torin	0.095111	11	0.0120791
VS-5584 (SB2343)						0.1096953	1	0	1	0	plate 1	enhancer	0.01063	12	0.005315
LY3023414	mTOR/PI3K	ATP compe	Endometria	https://clinic	2	0.1274994	1	0	1	0	plate 1	enhancer	0.0105597	13	0.004311
WAY-600	mTOR	ATP competitive			preclinical	0.1295706	1	0	1	0	plate 1	enhancer	0.2474581	14	0.123729
PP-121	tyrosine kinase/PI3Ks				preclinical	0.1301353	1	0	1	0	plate 1	enhancer	0.0136546	15	0.0061065
GSK690693	AKT		Solid Tumor	https://clinic	1	0.1349134	1	0	1	0	plate 1	enhancer	0.373052	16	0.1668339
Torin1	mTOR	ATP competitive			preclinical	0.1463757	1	0	1	0	Plate 2	Plate 2 Torin	0.0246257	17	0.0032907
AZ20	AKT		Solid Tumor	https://clinic	1	0.1998813	1	0	1	0	plate 1	enhancer	0.0678195	18	0.0276872
MK-2206 2HCl	AKT		Breast canc	https://clinic	2	0.2188268	1	0	1	0	plate 1	enhancer	0.0253739	19	0.0113476
Ipatasertib (GDC-0068)	AKT		Breast canc	https://clinic	2	0.2264934	1	0	1	0	plate 1	enhancer	0.0167548	20	0.0068401
WNK463	pan-WNK kinase				preclinical	0.2322077	1	0	1	0	plate 1	enhancer	0.0084485	21	0.0034491
Miransertib (ARQ 092)HCl	AKT		Breast canc	https://clinic	2	0.2323337	1	0	1	0	plate 1	enhancer	0.0284152	22	0.0127077
PRI-724	Wnt		Advanced S	https://clinic	1	0.2379219	1	0	1	0	plate 1	enhancer	0.0295334	23	0.0132078
ICG-001	Beta-catenin				preclinical	0.2382937	1	0	1	0	plate 1	enhancer	0.0348374	24	0.0142223
Apitolisib (GDC-0980, RG7422)	mTOR/PI3K	(CA,CB,CD,	Metastatic F	https://clinic	2	0.2386906	1	0	1	0	plate 1	enhancer	0.0115423	25	0.0051619
CHIR-124	CHK1				preclinical	0.2493176	1	0	1	0	plate 1	enhancer	0.1599948	26	0.0715519
AZD5363	AKT		Triple Nega	https://clinic	3	0.2497076	1	0	1	0	plate 1	enhancer	0.0198047	27	0.0080852
XL388	mTOR	ATP competitive			preclinical	0.261893	1	0	1	0	plate 1	enhancer	0.0200879	28	0.0089836
KU-0063794	mTOR	ATP competitive			preclinical	0.3152943	1	0	1	0	plate 1	enhancer	0.0297506	29	0.0121457
CCT128930	AKT				preclinical	0.3249396	1	0	1	0	plate 1	enhancer	0.0536815	30	0.0219154
WYE-354	mTOR	ATP competitive			preclinical	0.3259717	1	0	1	0	plate 1	enhancer	0.0304581	31	0.0136213
Pelitinib (EKB-569)	EGFR		Lung Cance	https://clinic	2	0.3574427	1	0	1	0	plate 1	enhancer	0.0209736	32	0.0093797
SC144	gp130				preclinical	0.3590762	1	0	1	0	plate 1	enhancer	0.0112468	33	0.0045915
CX-4945 (Silmitasertib)	CK2		Medulloblas	https://clinic	2	0.3607137	1	0	1	0	plate 1	enhancer	0.0136788	34	0.0068394
kira6	IRE1a				preclinical	0.3625398	1	0	1	0	plate 1	enhancer	0.02606	35	0.0106389
AT7867	AKT				preclinical	0.363375	1	0	1	0	plate 1	enhancer	0.0542424	36	0.0242579
Antimonyl (potassium tartrate trihydrate	EGFR				1	0.3698902	1	0	1	0	plate 1	enhancer	0.0058257	37	0.0023783
ETP-46464	ATR				preclinical	0.3709038	1	0	1	0	plate 1	enhancer	0.0299645	38	0.0122329
Quinacrine dihydrochloride hydrate	Moa/NFkB				approved fo	0.3856344	1	0	1	0	Plate 2	enhancer	0.0412447	39	0.0168381
Halofuginone						0.3875036	0	0	0	0	plate 1	non-significa	0.0227048	40	0.0092692
LY2874455						0.3893479	1	0	0	0	plate 1	non-significa	0.065983	41	0.0380953
CHLORHEXIDINE						0.3918598	0	0	0	0	plate 1	non-significa	0.0036366	42	0.0018183
AS1517499						0.3939989	0	0	0	0	plate 1	non-significa	0.0310537	43	0.0138876
CYC116	Aurora kinase		Advanced S	https://clinic	1	0.3965008	0	0	1	0	Plate 2	enhancer	0.031598	44	0.0141311
Resorantel						0.4009715	0	0	0	0	plate 1	non-significa	0.0384684	45	0.0172036
Rapamycin	mTOR	allosteric			approved fo	0.4076114	1	0	1	0	Plate 2	enhancer	0.0240315	46	0.0107472
Deforolimus (MK-8669)	mTOR	allosteric				0.4099051	0	0	0	0	plate 1	non-significa	0.0343694	47	0.0140313
Everolimus (RAD001)	mTOR	allosteric			approved fo	0.4129503	1	0	1	0	Plate 2	enhancer	0.0565811	48	0.0253038
Go 6976	PKC				preclinical	0.4144386	0	0	0	0	plate 1	non-significa	0.0526086	49	0.0235273
Dovitinib Dilactic acid (TKI258 Dilactic aci						0.4154465	0	0	0	0	plate 1	non-significa	0.0151735	50	0.0061945
JIB04 (NSC693627)						0.4177479	1	0	0	0	plate 1	non-significa	0.2077286	51	0.1038643
Sotrastaurin						0.4185701	0	0	0	0	plate 1	non-significa	0.0158173	52	0.0064574
MRT68921 HCl	ULK1/2				preclinical	0.4205838	0	1	1	0	Plate 2	enhancer	0.0641173	53	0.0261758
Diphenyleneiodonium chloride						0.4242223	0	0	0	0	plate 1	non-significa	0.0252451	54	0.0103063
Pevonedistat (MLN4924)						0.4334603	0	0	0	0	plate 1	non-significa	0.0648378	55	0.0289964
EAD1					preclinical	0.4353125	1	0	1	0	Plate 2	enhancer	0.0207367	56	0.0084657
PI-3065	p110δ				preclinical	0.4362665	0	0	1	0	Plate 2	enhancer	0.3276161	57	0.1337487
2-{2-[4-(trifluoromethoxy)phenyl]hydrazono}malononitrile						0.4447991	0	0	0	0	plate 1	non-significa	0.0328656	58	0.0134173
MITOXANTHRONE HYDROCHLORIDE						0.4509557	0	0	0	0	plate 1	non-significa	0.5044762	59	0.2256086
Nintedanib Ethanesulfonate Salt	FGFR				approved fo	0.4529669	0	0	1	0	Plate 2	enhancer	0.0076541	60	0.0038271
NSC 147340						0.4533718	0	0	0	0	plate 1	non-significa	0.0260702	61	0.0106431
OSI-420 (Desmethyl Erlotinib)						0.453973	0	0	0	0	plate 1	non-significa	0.0236376	62	0.00965
Ponatinib (AP24534)						0.454303	0	0	0	0	plate 1	non-significa	0.0193556	63	0.0079019
Tizanidine	adrenergic receptor				approved fo	0.4569585	0	0	1	0	Plate 2	enhancer	0.2011716	64	0.0899667
Gilteritinib (ASP2215)						0.457653	0	0	0	0	plate 1	non-significa	0.0382117	65	0.0155999
TAI-1						0.4609075	0	0	0	0	plate 1	non-significa	0.0298175	66	0.0121729
GNE-317						0.4623834	0	0	0	0	plate 1	non-significa	0.031395	67	0.0140403
4SC-202						0.4632391	0	0	0	0	plate 1	non-significa	0.0493538	68	0.0220717
5-AZACYTIDINE						0.4643668	0	0	0	0	plate 1	non-significa	0.0416008	69	0.0169834
PTC-209 HBr						0.4656881	0	0	0	0	plate 1	non-significa	0.0154361	70	0.0063018
Teniposide (Vumon)						0.4667568	0	0	0	0	plate 1	non-significa	0.0314718	71	0.0128483
NSC 697855						0.4689	0	0	0	0	plate 1	non-significa	0.0451103	72	0.020174
QUINACRINE HYDROCHLORIDE						0.4723758	0	0	0	0	plate 1	non-significa	0.0784013	73	0.0320072
NH125						0.4732728	0	0	0	0	plate 1	non-significa	0.0123522	74	0.0061761
CAY10603						0.4753053	0	0	0	0	plate 1	non-significa	0.0292575	75	0.0119443
YM155 (Sepantronium Bromide)						0.4757062	0	0	0	0	plate 1	non-significa	0.0422136	76	0.0188785
LDN-214117						0.4762989	0	0	0	0	plate 1	non-significa	0.0289947	77	0.0118371
FH-535						0.4776233	0	0	0	0	plate 1	non-significa	0.0293052	78	0.0119638
Ifenprodil Tartrate						0.4776972	0	0	0	0	Plate 2	non-significa	0.1951095	79	0.0796531
Alpelisib (BYL719)						0.479081	0	0	0	0	Plate 2	non-significa	0.0152577	80	0.0076289
Tyrphostin 9						0.479205	0	0	0	0	plate 1	non-significa	0.0079385	81	0.0035502
Encorafenib (LGX818)						0.4808861	0	0	0	0	plate 1	non-significa	0.023161	82	0.0103579
7-Ethylcamptothecin						0.4816454	0	0	0	0	plate 1	non-significa	0.0225692	83	0.0092138
HEXACHLOROPHENE						0.4825961	0	0	0	0	plate 1	non-significa	0.0345851	84	0.0141193
Temsirolimus (CCI-779, NSC 683864)						0.4833839	0	0	0	0	Plate 2	non-significa	0.0157544	85	0.0070456
GSK J4 HCl						0.4873003	0	0	0	0	plate 1	non-significa	0.0395598	86	0.0161502
Bevantolol hydrochloride						0.4875861	0	0	0	0	Plate 2	non-significa	0.0984414	87	0.0440243
AZD8835						0.4879895	0	0	0	0	Plate 2	non-significa	0.0318057	88	0.0129846
Digoxin						0.4898499	0	0	0	0	plate 1	non-significa	0.0435037	89	0.0194555
10-HYDROXYCAMTOTHECIN						0.4908967	0	0	0	0	plate 1	non-significa	0.0448434	90	0.0200546
ITF2357 (Givinostat)						0.4945143	0	1	0	0	Plate 2	non-significa	0.2922704	91	0.1193189
DICYCLOMINE HYDROCHLORIDE						0.4954352	0	0	0	0	Plate 2	non-significa	0.0786005	92	0.0320885
Marinopyrrole A (Maritoclax)						0.4957121	0	0	0	0	plate 1	non-significa	0.0406252	93	0.0165852
HARMINE						0.4966139	0	0	0	0	Plate 2	non-significa	0.0081899	94	0.0047285
Sorafenib						0.5017502	0	0	0	0	plate 1	non-significa	0.0282765	95	0.0126456
WS3						0.5018778	0	0	0	0	plate 1	non-significa	0.0211749	96	0.0094697
MONOCROTALINE						0.5044845	0	0	0	0	plate 1	non-significa	0.0264087	97	0.0107813
Afuresertib (GSK2110183)						0.5051321	0	0	0	0	plate 1	non-significa	0.0322863	98	0.0131808

Uprosertib (GSK2141795)						0.5074734	0	0	0	0	plate 1	non-significa	0.0208152	99	0.0084978
ATM/ATR Kinase Inhibitor						0.5085574	0	0	0	0	Plate 2	non-significa	0.0693716	100	0.0346858
CLOMIPRAMINE HYDROCHLORIDE						0.5111118	0	0	0	0	Plate 2	non-significa	0.0245171	101	0.0109644
PF-562271 HCl						0.5131386	0	0	0	0	plate 1	non-significa	0.0424039	102	0.0189636
S 38093						0.5163024	0	0	0	0	Plate 2	non-significa	0.0369114	103	0.015069
Voxtalisib (XL765, SAR245409)						0.5172421	0	0	0	0	plate 1	non-significa	0.0128684	104	0.0074296
A66						0.5172466	0	0	0	0	Plate 2	non-significa	0.0183728	105	0.0091864
Umeclidinium bromide						0.5190865	0	0	0	0	Plate 2	non-significa	0.0168364	106	0.0068734
BRL-15572						0.520273	0	0	0	0	Plate 2	non-significa	0.0369996	107	0.015105
Ivabradine HCl (Procoralan)						0.5228112	0	0	0	0	Plate 2	non-significa	0.0196975	108	0.0080415
3BDO						0.5230816	0	0	0	0	Plate 2	non-significa	0.0890363	109	0.0363489
Tiagabine						0.523709	0	0	0	0	Plate 2	non-significa	0.0355613	110	0.0177807
HarmineA?HCl						0.5273957	0	0	0	0	Plate 2	non-significa	0.0399996	111	0.0163297
Diclofenac Diethylamine						0.5286397	0	0	0	0	Plate 2	non-significa	0.0411287	112	0.0167907
LY2886721						0.5305237	0	0	0	0	Plate 2	non-significa	0.0306629	113	0.0125181
DOPAMINE HYDROCHLORIDE						0.5306352	0	0	0	0	Plate 2	non-significa	0.0239662	114	0.010718
Benztropine mesylate						0.5308342	0	0	0	0	Plate 2	non-significa	0.0190298	115	0.0077689
KN-93 Phosphate						0.5310959	0	0	0	0	Plate 2	non-significa	0.0304876	116	0.0136345
Tiotropium Bromide hydrate						0.5328715	0	0	0	0	Plate 2	non-significa	0.0328545	117	0.0134128
CPD000466354_LATANOPROST						0.5338674	0	0	0	0	Plate 2	non-significa	0.0219105	118	0.0097987
(+)-JQ1						0.5356412	0	0	0	0	Plate 2	non-significa	0.0335491	119	0.0136963
AZD5153						0.5358558	0	0	0	0	Plate 2	non-significa	0.0244622	120	0.0099866
Parecoxib						0.5387881	0	0	0	0	Plate 2	non-significa	0.0265972	121	0.0108583
A-317491						0.5396471	0	0	0	0	Plate 2	non-significa	0.0961607	122	0.0430044
VPS34-IN1						0.5437231	0	1	0	0	Plate 2	non-significa	0.0580372	123	0.025955
AG-879						0.5442517	0	0	0	0	plate 1	non-significa	0.0253345	124	0.0113299
OTX015						0.5445233	0	0	0	0	Plate 2	non-significa	0.1059149	125	0.0529574
GSK1324726A (I-BET726)						0.5449315	0	0	0	0	Plate 2	non-significa	0.0016388	126	0.0009461
DMSO						0.5464661	0	0	0	0	plate 1	Plate 1 DMS	0.0531326	128	0.0068029
PROPANTHELINE BROMIDE						0.5466069	0	0	0	0	Plate 2	non-significa	0.053855	129	0.0219862
Oxidopamine (hydrobromide)						0.5469577	0	0	0	0	Plate 2	non-significa	0.0638848	130	0.0260809
DMSO						0.5472519	0	0	0	0	Plate 2	Plate 2 DMS	0.0342745	131	0.0045801
MS436						0.5507526	0	0	0	0	Plate 2	non-significa	0.0714562	132	0.0291719
Methscopolamine (Pamine)						0.5510995	0	0	0	0	Plate 2	non-significa	0.0233401	133	0.010438
XL765						0.5515507	0	0	0	0	Plate 2	non-significa	0.0411126	134	0.0167841
SAHA						0.5528182	0	0	0	0	Plate 2	non-significa	0.0235614	135	0.0117807
ERK5-IN-1						0.5533157	0	0	0	0	Plate 2	non-significa	0.0147428	136	0.0060187
M 344						0.5564232	0	0	0	0	Plate 2	non-significa	0.0396452	137	0.0177299
LRRK2-IN-1						0.557018	0	0	0	0	Plate 2	non-significa	0.0181447	138	0.0090723
ABBV-075 (Mivebresib)						0.5590692	0	0	0	0	Plate 2	non-significa	0.0445941	139	0.0199431
Bortezomib (Velcade)						0.5620114	0	0	0	0	plate 1	non-significa	0.0110632	140	0.0045165
BMS-708163 (Avagacestat)						0.5624521	0	0	0	0	Plate 2	non-significa	0.0763341	141	0.0341376
Resminostat (RAS2410)						0.5631163	0	0	0	0	Plate 2	non-significa	0.0185052	142	0.0075547
ACY-1215						0.5633997	0	0	0	0	Plate 2	non-significa	0.0595684	143	0.0243187
AZD3514						0.5640319	0	0	0	0	Plate 2	non-significa	0.0323208	144	0.0131949
PF-4989216						0.5669957	0	0	0	0	Plate 2	non-significa	0.0728669	145	0.0420697
Pracinostat (SB939)						0.5679025	0	1	0	0	Plate 2	non-significa	0.0276636	146	0.0138318
Ramosetron Hydrochloride						0.5712824	0	0	0	0	Plate 2	non-significa	0.0596463	147	0.0243505
SNS-314 Mesylate						0.5738762	0	0	0	0	plate 1	non-significa	0.0220959	148	0.0090206
PHENYLBUTAZONE						0.5758878	0	0	0	0	plate 1	non-significa	0.0425207	149	0.017359
Torin 2						0.5772246	0	0	0	0	plate 1	non-significa	0.0667603	150	0.0272548
Palomid 529						0.5784134	0	0	0	0	Plate 2	non-significa	0.0750008	151	0.0335414
PFI-1(PF-6405761)						0.5794537	0	0	0	0	Plate 2	non-significa	0.0157621	152	0.0064348
PX105684						0.5876016	0	1	0	0	Plate 2	non-significa	0.038139	153	0.0170563
I-BET						0.5970632	0	0	0	0	Plate 2	non-significa	0.0442667	154	0.0221333
NVP-LBH589						0.5981574	0	1	0	0	Plate 2	non-significa	0.0332703	155	0.0148789
PHA-665752						0.5991579	0	0	0	0	Plate 2	non-significa	0.0765795	156	0.0342474
MCCSL-MWW-2-019						0.6109399	0	1	0	0	Plate 2	non-significa	0.0172352	157	0.0070362
Dacinostat						0.6113578	0	1	0	0	Plate 2	non-significa	0.0471719	158	0.0192578
IMD0354						0.6127914	0	0	0	0	plate 1	non-significa	0.0936296	159	0.0418724
Sabutoclax						0.6591429	0	0	0	0	plate 1	non-significa	0.0303162	160	0.0123765
Citarinostat (ACY-241)						0.676874	0	0	0	0	plate 1	non-significa	0.0202416	161	0.0090523
Toosendanin	neurotrasmission blocker				preclinical	0.87035	0	1	0	1	Plate 2	inhibitor	0.0746771	162	0.0333966
CC-115	DNA-PK, mTOR		glioblastoma	https://clinic	2	3.7413283	0	1	0	1	plate 1	inhibitor	0	164	0
Rocuronium bromide	acetylcholine receptor				approved as	3.2967386	0	0	0	1	Plate 2	inhibitor	0	163	0
DAUNOMYCIN HYDROCHLORIDE	DNA synthesis		multiple car	https://www.cancer.gov/		-0.296564	0	1	1	1	Plate 2	inhibitor	0	165	0
Obatoclax Mesylate (GX15-070)	BCL2		Lung Cance	https://clinic	3	-0.171788	0	1	1	1	Plate 2	inhibitor	0	166	0

Fig. S1

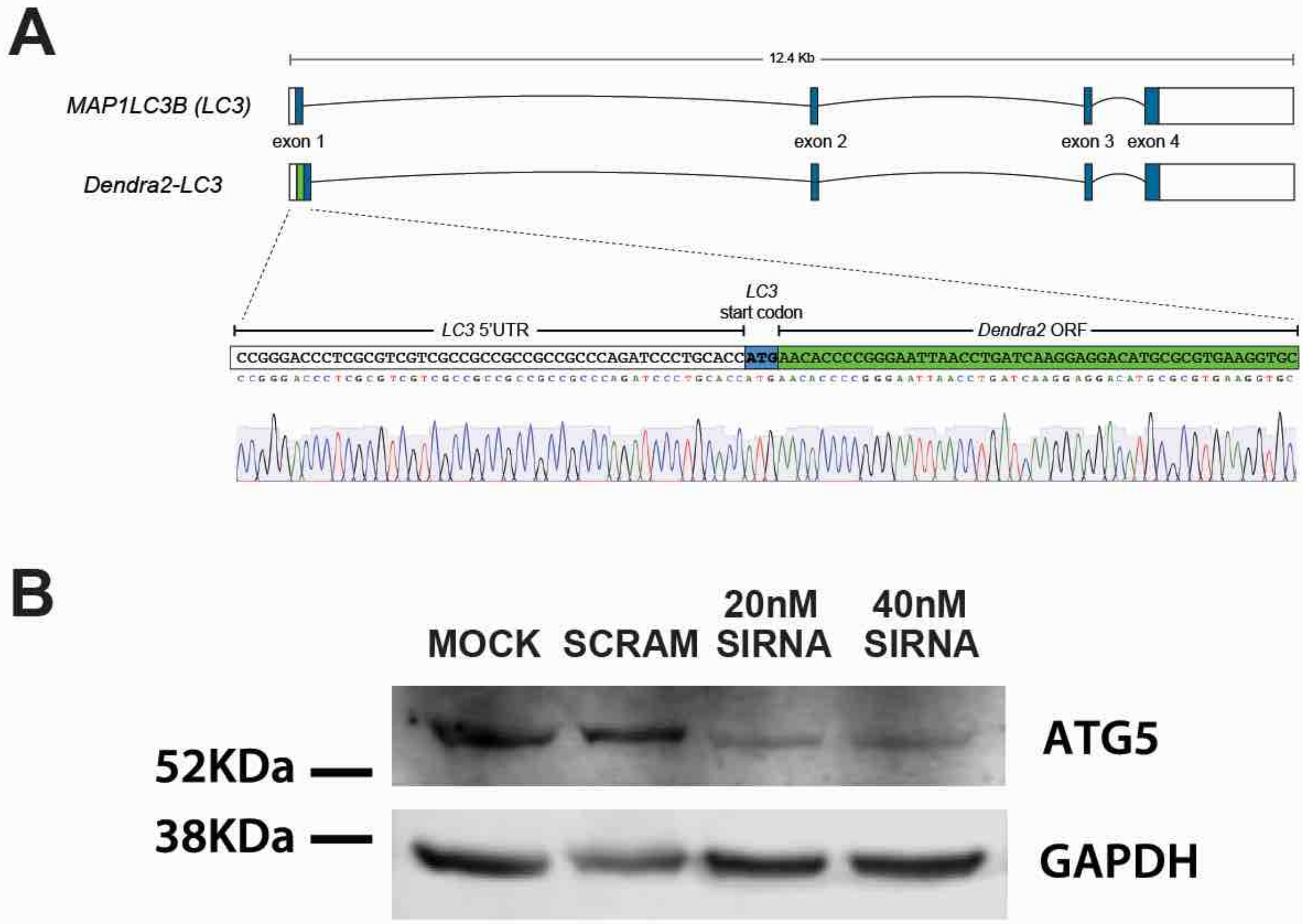
A
12.4 Kb
MAP1LC3B (LC3)
exon 1
exon 2
exon 3
exon 4
Dendra2-LC3
LC3 start codon
LC3 5'UTR
Dendra2 ORF
CCGGGACCCTCGCGTCGTCGCCGCCGCCGCCGCCCAGATCCCTGCACCATGAACACCCCGGGAATTAACCTGATCAAGGAGGACATGCGCGTGAAGGTGC
B
MOCK
SCRAM
20nM SIRNA
40nM SIRNA
52KDa
38KDa
ATG5
GAPDH

Fig. S2

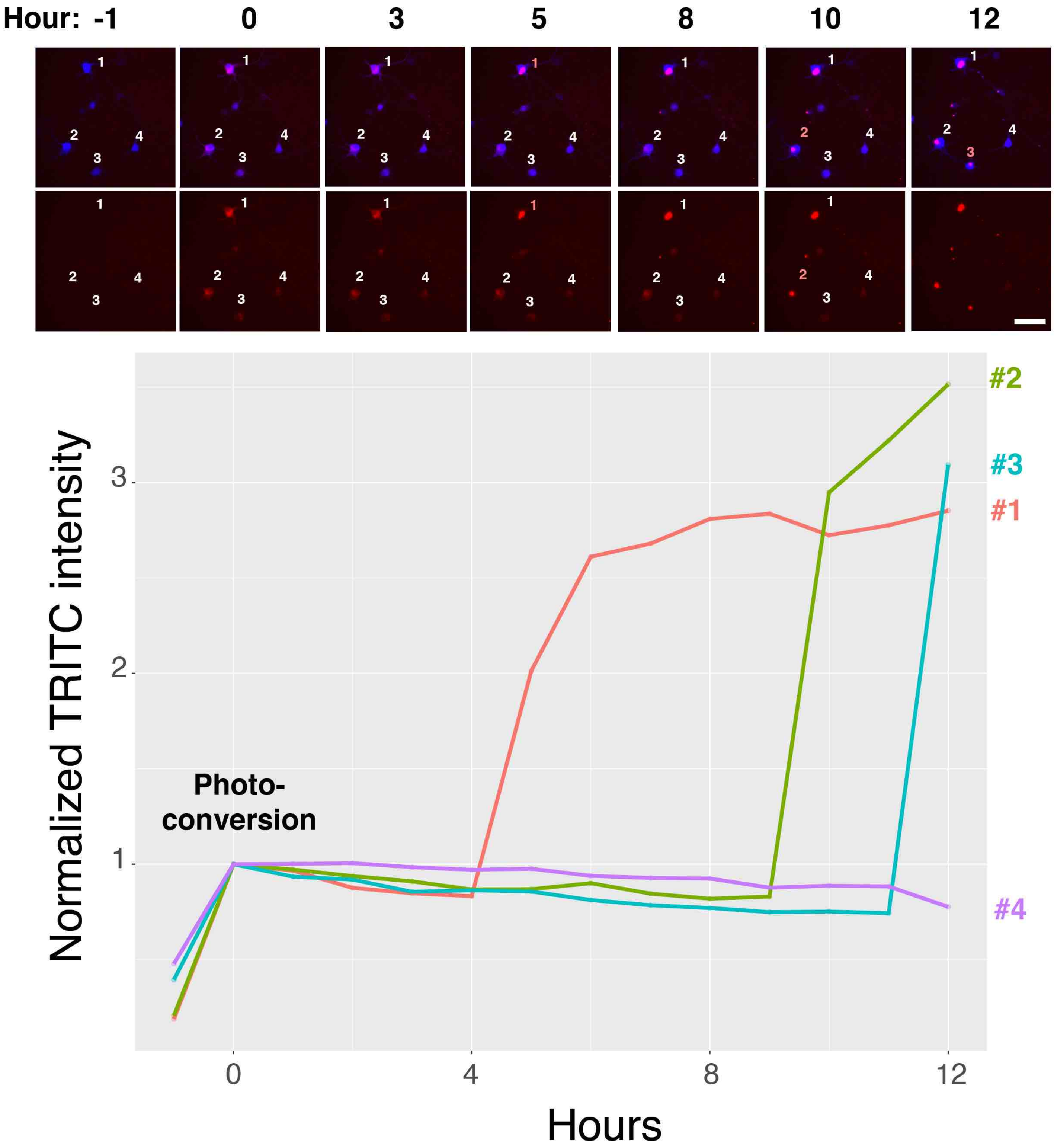
Hour: -1
0
3
5
8
10
12
1
2
3
4
Normalized TRITC intensity
Hours
Photo-
conversion
#2
#3
#1
#4
1
2
3
0
4
8
12

Fig. S3

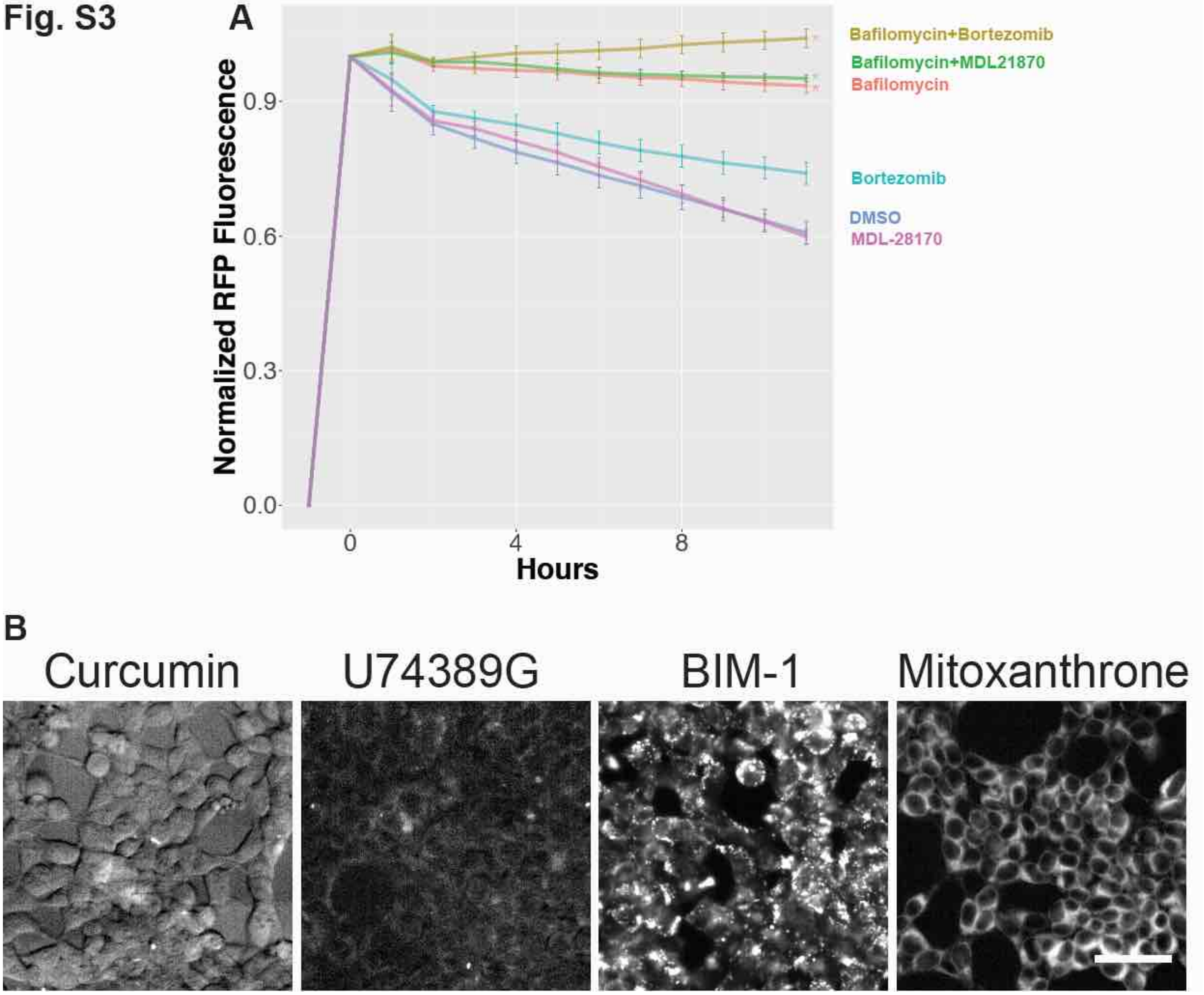

A
Normalized RFP Fluorescence
0.0
0.3
0.6
0.9
0
4
8
Hours
Bafilomycin+Bortezomib
Bafilomycin+MDL21870
Bafilomycin
Bortezomib
DMSO
MDL-28170
B
Curcumin
U74389G
BIM-1
Mitoxanthrone

Fig. S4

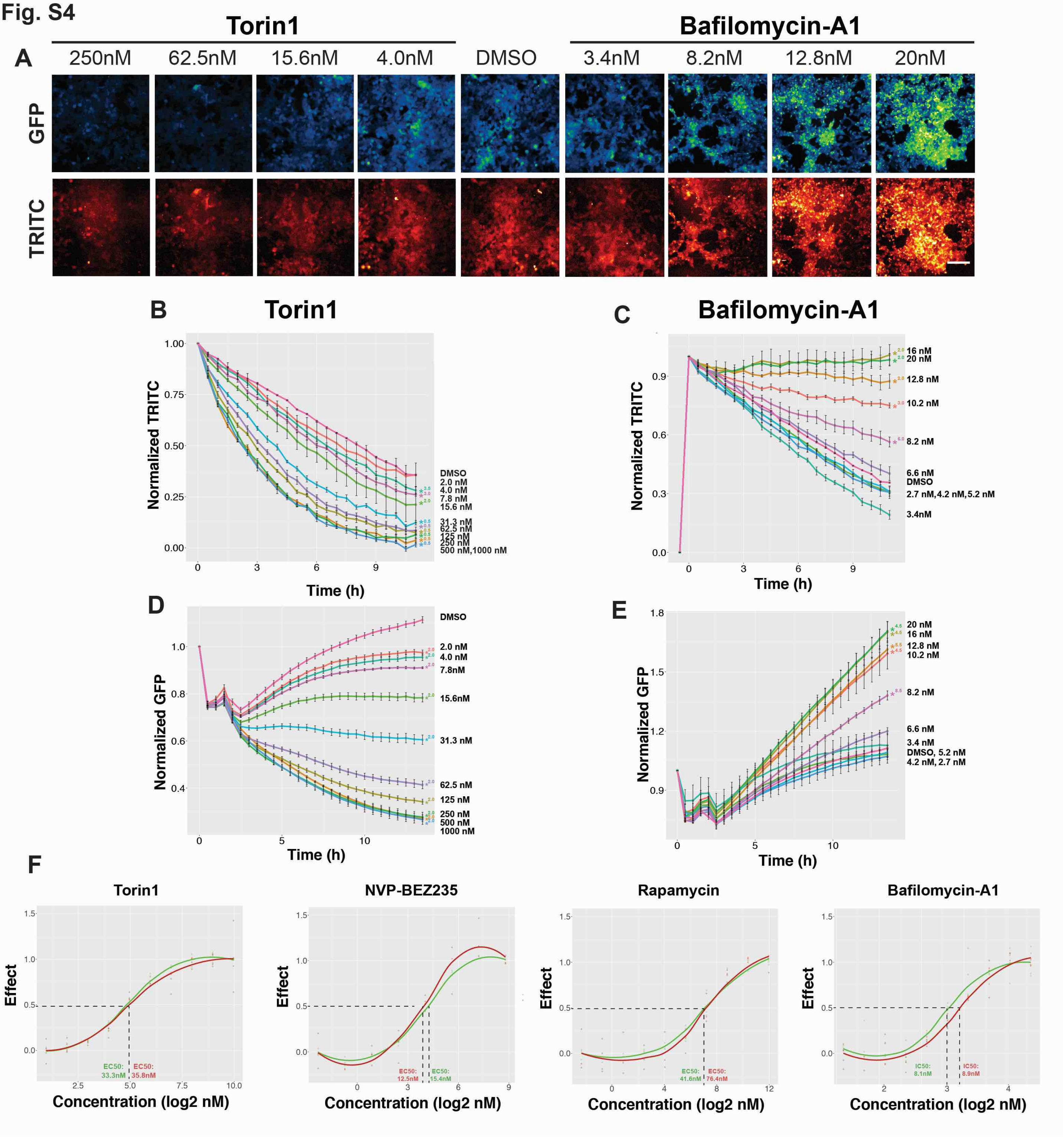
A
Torin1
Bafilomycin-A1
250nM
62.5nM
15.6nM
4.0nM
DMSO
3.4nM
8.2nM
12.8nM
20nM
GFP
TRITC
B
Torin1
Normalized TRITC
Time (h)
DMSO
2.0 nM
4.0 nM
7.8 nM
15.6 nM
31.3 nM
62.5 nM
125 nM
250 nM
500 nM,1000 nM
C
Bafilomycin-A1
Normalized TRITC
Time (h)
16 nM
20 nM
12.8 nM
10.2 nM
8.2 nM
6.6 nM
DMSO
2.7 nM,4.2 nM,5.2 nM
3.4nM
D
Normalized GFP
Time (h)
DMSO
2.0 nM
4.0 nM
7.8nM
15.6nM
31.3 nM
62.5 nM
125 nM
250 nM
500 nM
1000 nM
E
Normalized GFP
Time (h)
20 nM
16 nM
12.8 nM
10.2 nM
8.2 nM
6.6 nM
3.4 nM
DMSO, 5.2 nM
4.2 nM, 2.7 nM
F
Torin1
NVP-BEZ235
Rapamycin
Bafilomycin-A1
Effect
EC50: 33.3nM
EC50: 35.8nM
EC50: 12.5nM
EC50: 15.4nM
EC50: 41.6nM
EC50: 76.4nM
IC50: 8.1nM
IC50: 8.9nM
Concentration (log2 nM)

Fig. S5

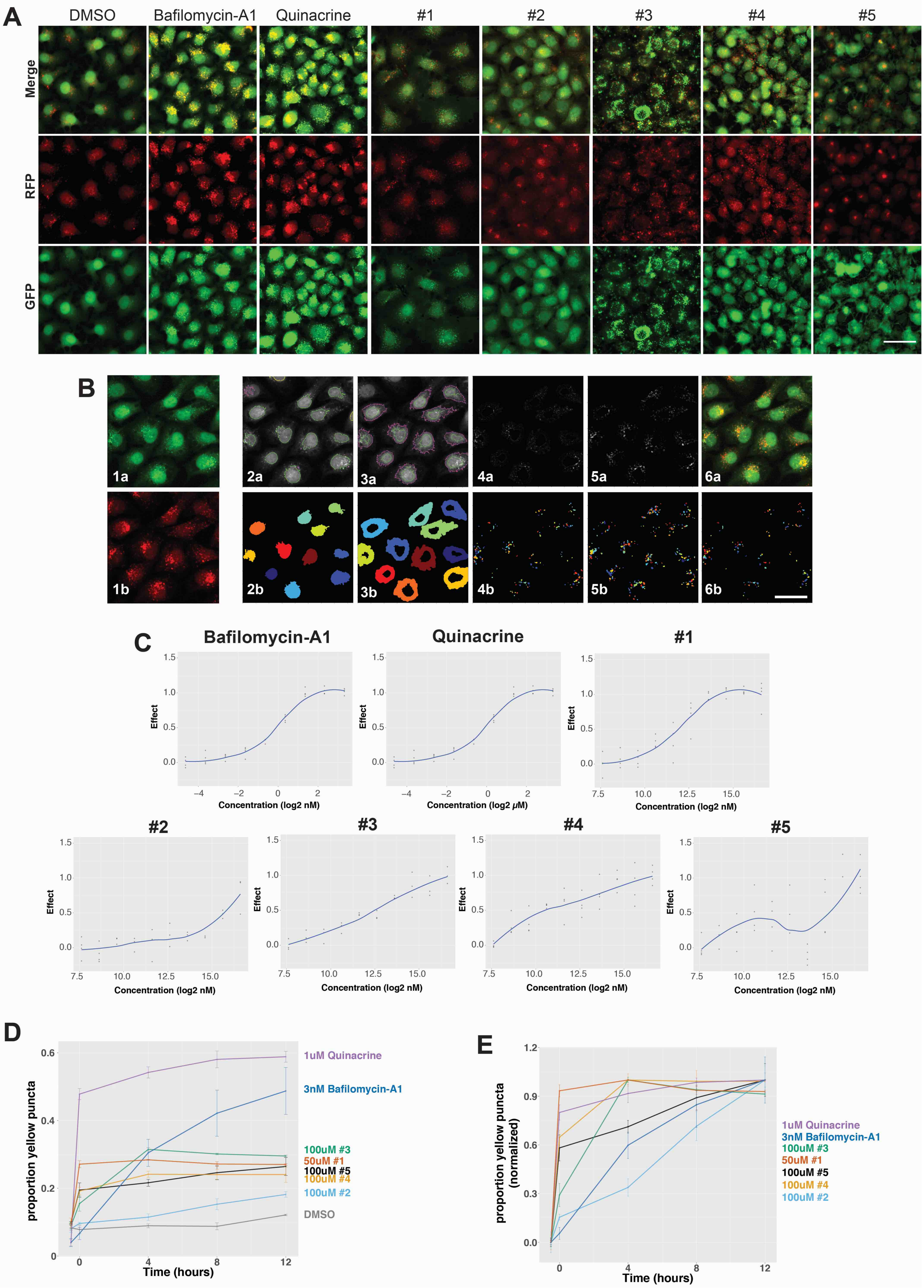
A
DMSO
Bafilomycin-A1
Quinacrine
#1
#2
#3
#4
#5
Merge
RFP
GFP
B
1a
1b
2a
2b
3a
3b
4a
4b
5a
5b
6a
6b
C
Bafilomycin-A1
Quinacrine
#1
#2
#3
#4
#5
Effect
Concentration (log2 nM)
Concentration (log2 μM)
D
proportion yellow puncta
Time (hours)
1uM Quinacrine
3nM Bafilomycin-A1
100uM #3
50uM #1
100uM #5
100uM #4
100uM #2
DMSO
E
proportion yellow puncta (normalized)
Time (hours)
1uM Quinacrine
3nM Bafilomycin-A1
100uM #3
50uM #1
100uM #5
100uM #4
100uM #2

Fig. S6

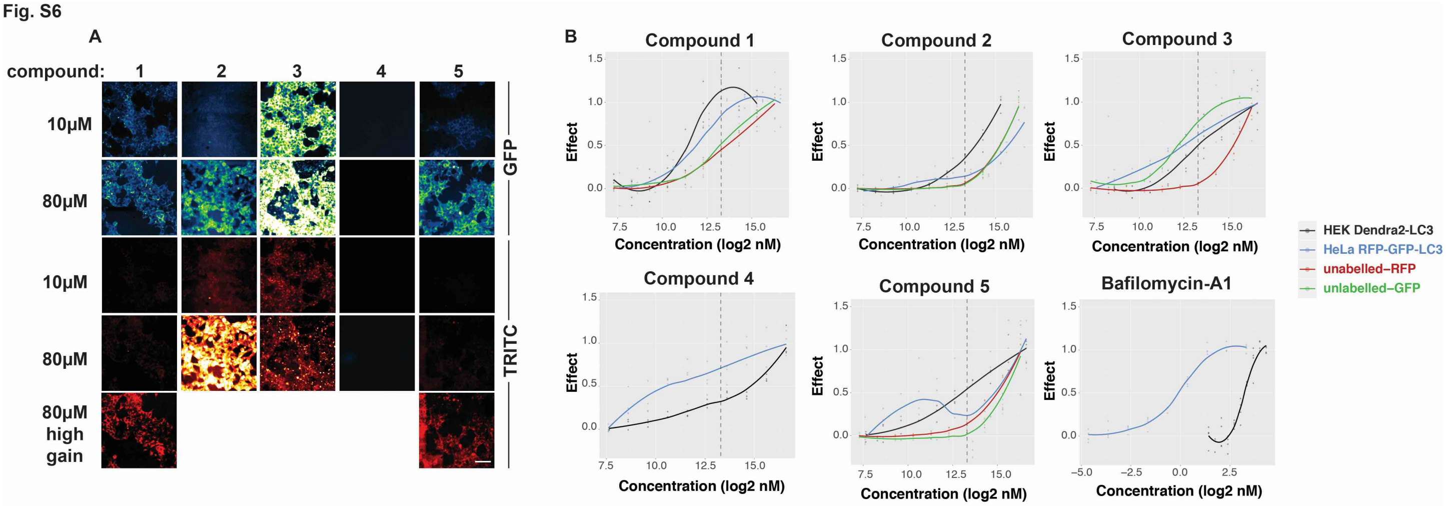

A
compound:
1
2
3
4
5
10µM
80µM
10µM
80µM
80µM high gain
GFP
TRITC
B
Compound 1
Compound 2
Compound 3
Compound 4
Compound 5
Bafilomycin-A1
Effect
Concentration (log2 nM)
HEK Dendra2-LC3
HeLa RFP-GFP-LC3
unabelled–RFP
unlabelled–GFP

Fig. S7

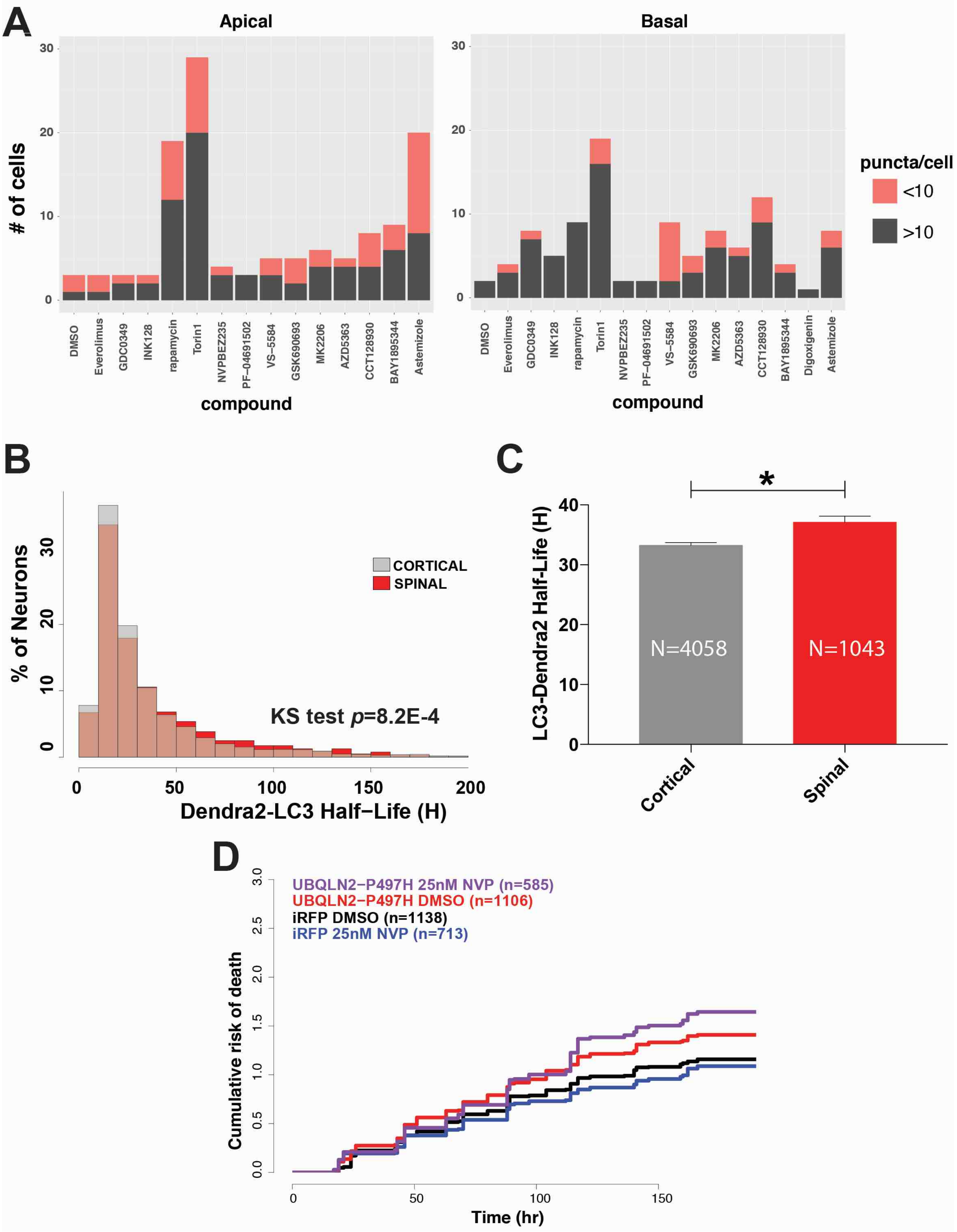
A
Apical
Basal
# of cells
puncta/cell
<10
>10
DMSO
Everolimus
GDC0349
INK128
rapamycin
Torin1
NVPBEZ235
PF−04691502
VS−5584
GSK690693
MK2206
AZD5363
CCT128930
BAY1895344
Digoxigenin
Astemizole
compound
B
% of Neurons
CORTICAL
SPINAL
KS test p=8.2E-4
Dendra2-LC3 Half−Life (H)
C
LC3-Dendra2 Half-Life (H)
N=4058
N=1043
Cortical
Spinal
D
UBQLN2−P497H 25nM NVP (n=585)
UBQLN2−P497H DMSO (n=1106)
iRFP DMSO (n=1138)
iRFP 25nM NVP (n=713)
Cumulative risk of death
Time (hr)
